# Demographic Factors Associated with Postoperative Complications in Primary Bariatric Surgery: A Rapid Review

**DOI:** 10.1007/s11695-025-07784-x

**Published:** 2025-03-13

**Authors:** Jocelin Hon, Paul Fahey, Mohammad Ariya, Milan Piya, Alex Craven, Evan Atlantis

**Affiliations:** 1 School of Medicine, Western Sydney University, Campbelltown, Australia; 2 School of Health Sciences, Western Sydney University, Campbelltown, Australia; 3 South Western Sydney Metabolic Rehabilitation and Bariatric Program, Camden and Campbelltown Hospitals, Campbelltown, Australia; 4 Department of Surgery, Austin Health, Melbourne, Australia

**Keywords:** Obesity, Bariatric, Bariatric surgery, Postoperative complications, Demographic factors

## Abstract

**Background:**

Bariatric surgery is highly effective for the management of severe obesity, but its safety profile is not completely understood. This review aimed to synthesise evidence linking demographic factors to postoperative complications and mortality following primary bariatric surgery.

**Methods:**

We searched Medline for observational studies of adult patients linking demographic factors to postoperative complications of primary bariatric surgery published from 2017 to 2022. Risk ratios (RR) with 95% confidence intervals (95% CI) were calculated and pooled using random effect meta-analysis. Heterogeneity was quantified using the *I*^2^ statistic and tested for statistical significance using the Q-statistic. Sensitivity analyses were used to explore potential sources of heterogeneity.

**Results:**

A total of 71 observational studies (69 cohort, 2 case–control) were reviewed and appraised. Older age was consistently associated with increased risks of postoperative mortality (RR = 2.62, 95% CI 1.63–4.23, *I*^2^ = 42.04%), serious complications (RR = 1.76, 95% CI 1.09–2.82, *I*^2^ = 93.24%), anastomotic leak (RR = 1.64, 95% CI 1.04–2.58, *I*^2^ = 61.09%), and haemorrhage (RR = 1.44, 95% CI 1.07–1.94, *I*^2^ = 45.25%). Male sex was associated with increased anastomotic leak (RR = 1.39, 95% CI 1.04–1.87, *I*^2^ = 72.36%). Sensitivity analyses did not identify sources of bias. Socioeconomic factors, including Black/African American race, low financial status, and marital status (mixed results), were linked to higher complication risks in some studies.

**Conclusions:**

Older age and certain demographic factors (male sex, Black/African American race, low financial status, marital status) were associated with increased risks of postoperative complications following primary bariatric surgery.

**Supplementary Information:**

The online version contains supplementary material available at 10.1007/s11695-025-07784-x.

## Introduction

Bariatric surgery is the most effective treatment for obesity and associated complications compared to non-surgical management options [1–3]. This procedure has had a surge in popularity worldwide where the total number of surgical and endoluminal procedures performed increased substantially in recent years [4]. Current international clinical guidelines recommend bariatric surgery for adults with body mass index (BMI) greater than 35 kg/m^2^, regardless of comorbidities, and consideration for those with a BMI of 30–34.9 kg/m^2^ and metabolic disease who have had unsuccessful non-surgical treatment attempts to lose weight or improve their comorbidities [5].

Current understanding about bariatric surgery focuses on its benefits in terms of weight loss and improvements in weight-related complications [6, 7]. When compared to non-surgical treatment options, bariatric surgery is more effective in achieving weight loss after 12 and 24 months, including a recent systematic review reporting a mean weight loss of 30 kg at 20 years [1–3, 8]. Some studies even reported net weight gain 2 and 10 years after conventional treatment while surgical patients consistently lost weight [1, 2]. Bariatric surgery has been shown to provide further health benefits for individuals with weight-related complications, including type 2 diabetes mellitus. Research has demonstrated improvements in T2DM following substantial weight loss of ≥ 10%, with bariatric surgery being the most cost-effective intervention [9]. Furthermore, observational evidence suggests a direct linear relationship between weight loss beginning from at least 2% and improvements in glycated haemoglobin and fasting glucose [10]. Although bariatric surgery is highly effective in managing severe obesity and its associated complications, further research is required to better understand its safety profile.

While bariatric surgery rates are rising, a clear understanding of patient demographic risk factors for postoperative complications remains unclear. Although some studies have reported postoperative complications as secondary outcomes [6, 11–14], there is insufficient evidence specifically focused on the safety of bariatric surgery in relation to patient demographics and their influence on complication risks. In the USA in 2022, the postoperative complication rate ranged between 2 and 13% for laparoscopic sleeve gastrectomy (LSG) and between 1 and 15% for Roux-en-Y gastric bypass (RYGB) [15]. Inconsistencies exist within the available literature regarding complications risks. For instance, observational research in Australia reported that older age and female sex were statistically significant risk factors for postoperative complications [6, 12]. In contrast, a Canadian cohort study reported no association between postoperative complications and age [16].

To address these gaps and inconsistencies, this rapid review aims to synthesise evidence on the association between patient demographic risk factors and postoperative complications, including mortality, following primary bariatric surgery. This may inform clinical guidelines about bariatric surgery, promoting informed decision-making for clinicians and their patients. It may also promote the implementation of measures, such as enhanced monitoring and specific perioperative care, to avert potential postoperative complications.

## Materials and Methods

The development of this rapid systematic review protocol was guided by previous research [17–19], the Centre for Review and Dissemination’s Guidance for undertaking reviews in healthcare [20], the Joanna Briggs Institute methodology [21], and the Preferred Reporting Items for Systematic Review and Meta-Analysis Protocols statement [22]. This rapid systematic review did not involve patients and the public in the protocol development.

### Research Question

Does the likelihood of developing postoperative complications of primary bariatric surgery vary across different patient demographic groups?

### Eligibility

Using modified versions of the Population, Interventions, Comparators, and Outcomes (PICO) framework below, we developed a research question and selected study eligibility criteria [23].

This study focused on adult patients aged 18 or over who underwent primary bariatric surgery worldwide, regardless of the healthcare setting. Patient factors such as age, gender, ethnicity, socioeconomic status, education level, employment status, and geographical location were considered independent variables. The primary outcomes of interest were any postoperative complications arising from bariatric surgery. Mortality was assessed as a secondary outcome. The review included comparative observational studies (e.g. cohort, cross-sectional, and case–control) published in English. These studies investigated the association between patient demographic risk factors (independent variables) and outcomes (dependent variables), regardless of follow-up duration.

### Search Strategy, Information Sources, and Study Selection

In collaboration with the subject matter expert (EA) and an academic librarian, we developed a search strategy. On 26 July 2022, we used keywords and phrases relevant to the field of study to search the Medline database for potentially relevant articles. We refined the search results by limiting them to English-language studies involving human adults and published between 2017 and the present. All identified records were exported from the database into EndNote 20 reference management software, and duplicate records were removed where feasible. Subsequently, we thoroughly examined all available full-length reports obtained from these records to assess their potential for inclusion. Additionally, we searched the reference lists of included studies for potentially relevant articles. The complete electronic database search strategy is presented in Supplementary Table S1.

Initially, the independent variable of patient factors encompassed baseline clinical factors (e.g. comorbidities, height, weight, BMI) and behavioural factors (e.g. smoking, alcohol), in addition to demographic factors. However, following the initial second screen, the number of studies (*n* = 133) remained excessive for a rapid review. Consequently, we opted to restrict patient factors to demographic characteristics solely.

### Data Collection Process

We extracted data as published from the full-text articles into a table created in Microsoft Word for iterative editing. Data extraction included the following: study design, year and country of study, sample size, inclusion, and exclusion criteria, setting and population, patient demographic factor, postoperative complications, statistical analysis, and association between demographic factors and complications. For studies which reported more than five postoperative complications, we extracted results for the five most common complications based on the rates reported in the studies, regardless of their statistical significance. Definitions for all complications reported are presented in Supplementary Table S2. We did not seek additional information from the authors of the selected studies.

### Risk of Bias in Individual Studies

To assess the methodological quality of the studies included for review, we used the standardised critical appraisal tools from the Joanna Briggs Institute [24, 25]. This approach facilitated a systematic evaluation of each study’s design, methodology, and potential sources of bias. The information gathered from this assessment informed our subsequent discussions on the overall strength of the evidence presented in the synthesised results.

### Synthesis of Results

Our results are presented in both narrative form within the “Results” section and in summary tables for a comprehensive overview. Where appropriate, we conducted a meta-analysis to quantitatively synthesise the findings and derive more robust conclusions.

All demographic predictors used were dichotomised, and the number in each category and the number of adverse events in each category were recorded. Where the number of adverse events was not provided, these were estimated by back-transforming from relative risks or odds ratios provided, except for studies with case–control designs which were excluded. Studies reporting analyses of time till adverse event and studies presenting results for age as a continuous variable were also excluded.

Risks adjusted for other predictors (such as BMI or medical history variables) were avoided wherever possible. The demographic variables (particularly age) have potential associations with most other predictors of adverse events; adjusted results would provide underestimates of the relationship of interest between the demographic variables and adverse events. Where only adjusted results were available, these results were included in the main analyses and then omitted in follow-up sensitivity analyses.

Reference categories for sex, age, and race were defined as males, ≥ 65 years of age, and white race, respectively. Where reported results were presented for more than two categories of age or race, frequency counts were summed to create the two categories. Where no age cut point corresponded to ≥ 65 years, we included any cut point between ≥ 60 years or ≥ 70 years as acceptable alternatives. However, the main analysis was followed by a sensitivity analysis including only studies for which reported results for ≥ 65 years exactly.

Separate random effects meta-analyses were used to pool the risk ratios for each demographic predictor for each adverse outcome where at least two independent results were available for pooling. Studies reporting zero relevant adverse events for both demographic groups were excluded from that analysis. In cases where there were zero relevant adverse events in one of the two demographic categories, a correction factor of 0.5 was added to all frequency counts. Results are presented in Forest plots showing pooled RRs with 95% CIs.

Forest plots are used to visually present both individual and pooled study results. Heterogeneity is quantified using the *I*^2^ statistic (percentage of total variation attributable to between-study differences) and *p*-values from the Q-test of heterogeneity to assess the statistical significance of the observed variation.

Publication bias, where studies with statistically significant results are more likely to be published, was evaluated using funnel plots and Egger’s test. However, these analyses were only performed when there were at least ten between-group comparisons available, as recommended by the Cochrane Handbook [26].

All analyses were conducted using the metafor() package in R software. The significance level was set to 0.05.

## Results

### Study Selection

**Fig. 1:**
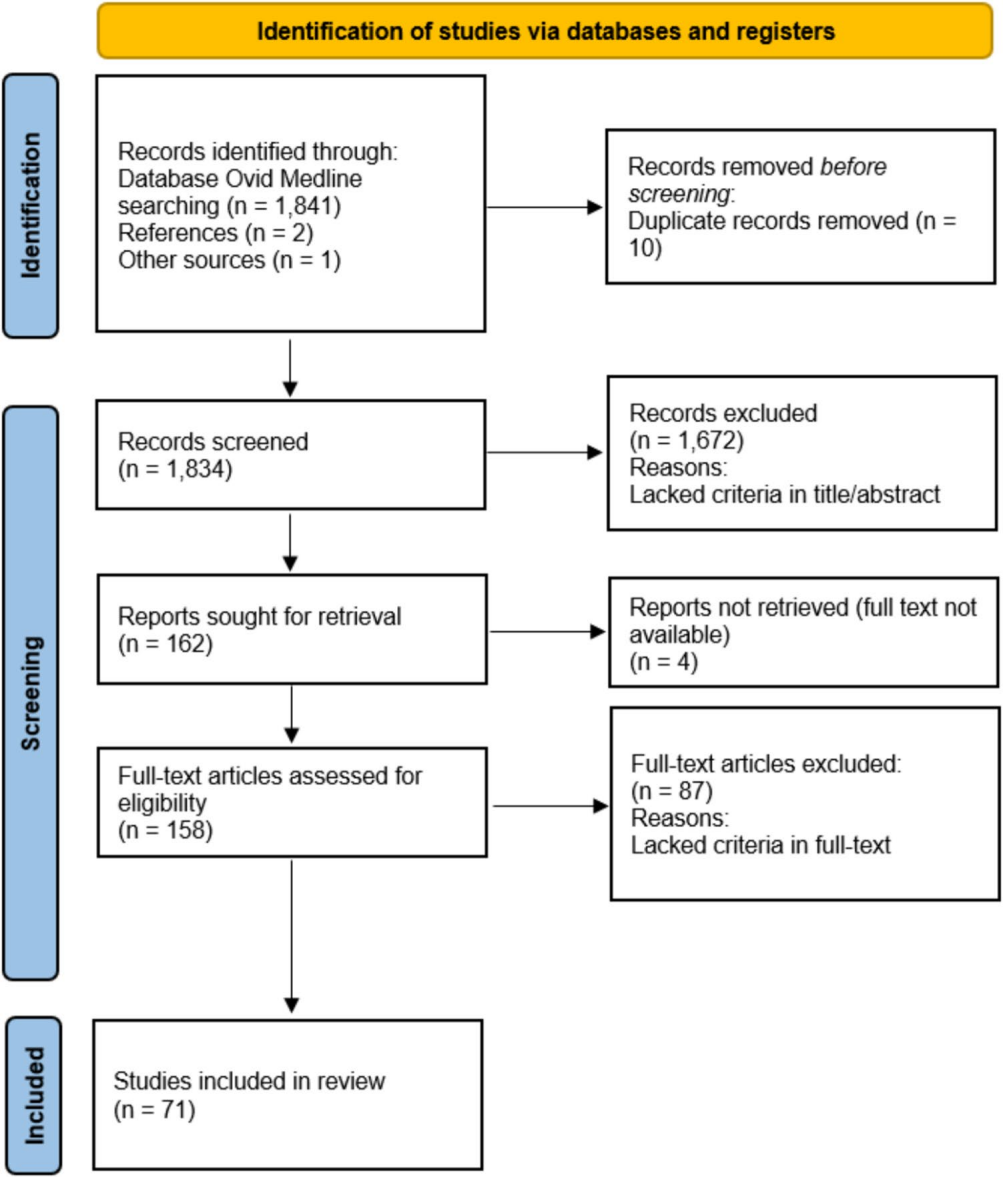
PRISMA flow diagram [27]

A flow diagram of the study selection process is presented in Fig. 1. Our search strategy identified 1831 records after the removal of ten duplicates. Three additional studies were identified from other sources. Out of these, 1672 records were excluded following the initial screening, and four records were excluded due to the unavailability of full-text versions, leaving 158 full-text articles for a second screening. Upon further evaluation, 87 additional records were excluded for reasons summarised in Fig. 1 and detailed in Supplementary Section S3.

### Study Characteristics

A summary of the results and characteristics of the included studies is presented in Supplementary Section S4.

### Risk of Bias Within Studies

We present the results of our quality assessment of each study in Supplementary Table S5. Cohort studies had five [28–33], six [34–39], seven [40–73], eight [74–94], nine [95], and ten [96] out of 11 quality items, and case–control studies had nine [97, 98] out of ten quality items.

### Results of Synthesis

The three most frequently reported demographic characteristics (age, sex, and race) were considered for pooling. All potential overlaps between source data were identified for each overlap group, and to avoid double counting of the same patients, only the study with the largest sample size was included in the pooling. For race, all studies were conducted using US data (with or without Canada). All these studies reported results from MBSAQIP or data sets which likely overlapped the MBSAQIP. To avoid multiple counting of the same patients, we did not pool results for race.

### Mortality

**Fig. 2:**
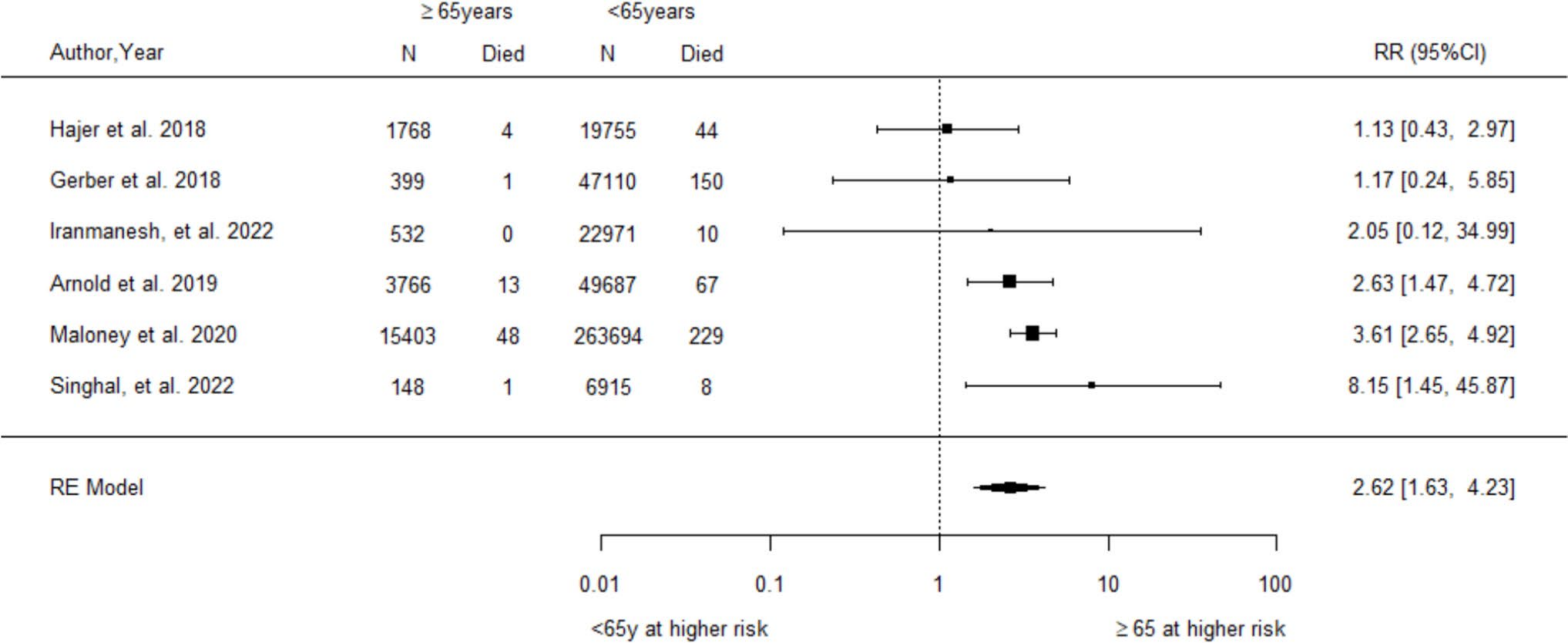
Forest plot of relative risk for postoperative mortality associated with older relative to younger age

For age, the pooled analysis of six studies [28, 33, 34, 47, 57, 82] demonstrated a significantly increased relative risk of postoperative mortality among older compared to younger patients (RR = 2.62, 95% CI 1.63–4.23, Fig. 2). While a moderate level of heterogeneity was observed (*I*^2^ = 42%), the Q-statistic test did not reach statistical significance (*p*-value = 0.151). No studies adjusted results for comorbidities, but a sensitivity analysis excluding the one study with different age groupings produced a slightly higher pooled estimate (RR = 3.33, 95% CI 2.55–4.34, Supplementary Section S6 Fig. S6.1).

After excluding studies with overlapping results, there were insufficient studies available to pool results of mortality by sex.

### Anastomotic Leak

**Fig. 3:**
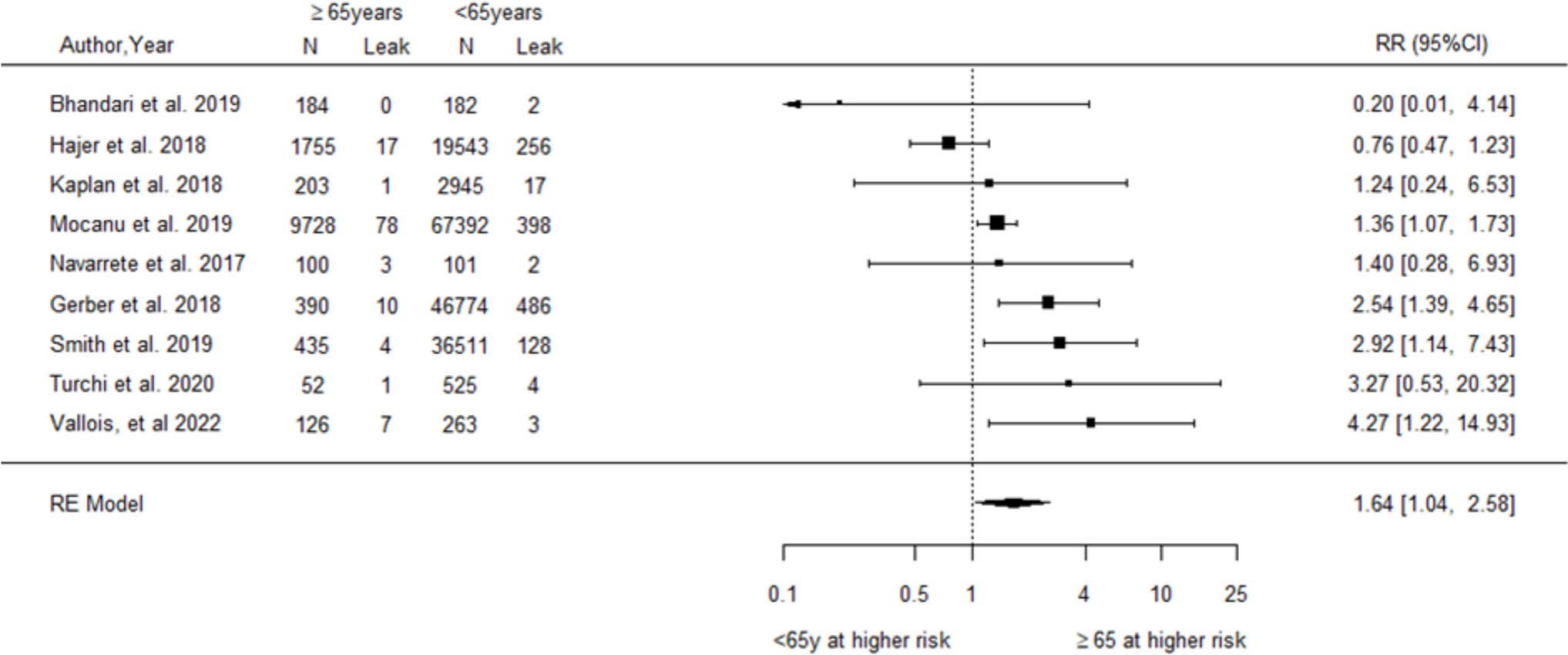
Forest plot of relative risk for postoperative anastomotic leak associated with older relative to younger age

For age, the pooled analysis of nine studies [33, 37, 38, 40, 51, 64, 67, 76, 82] demonstrated a significantly increased relative risk of postoperative anastomotic leak among older compared to younger patients (RR = 1.64, 95% CI 1.04–2.58, Fig. 3). The analysis detected a moderate to high level of heterogeneity between studies (*I*^2^ = 61.1%, Q-statistic *p*-value = 0.023). Egger’s test detected no evidence of publication bias (*p* = 0.931). Sensitivity analyses excluding studies with adjusted results for comorbidities and another analysis excluding one study with different age groupings produced similar pooled results (RR = 1.46, 95% CI 0.89–2.38, Supplementary Section S6 Fig. S6.2 and RR = 2.47, 95% CI 1.50–4.07, Supplementary Section S6 Fig. S6.3, respectively).

**Fig. 4:**
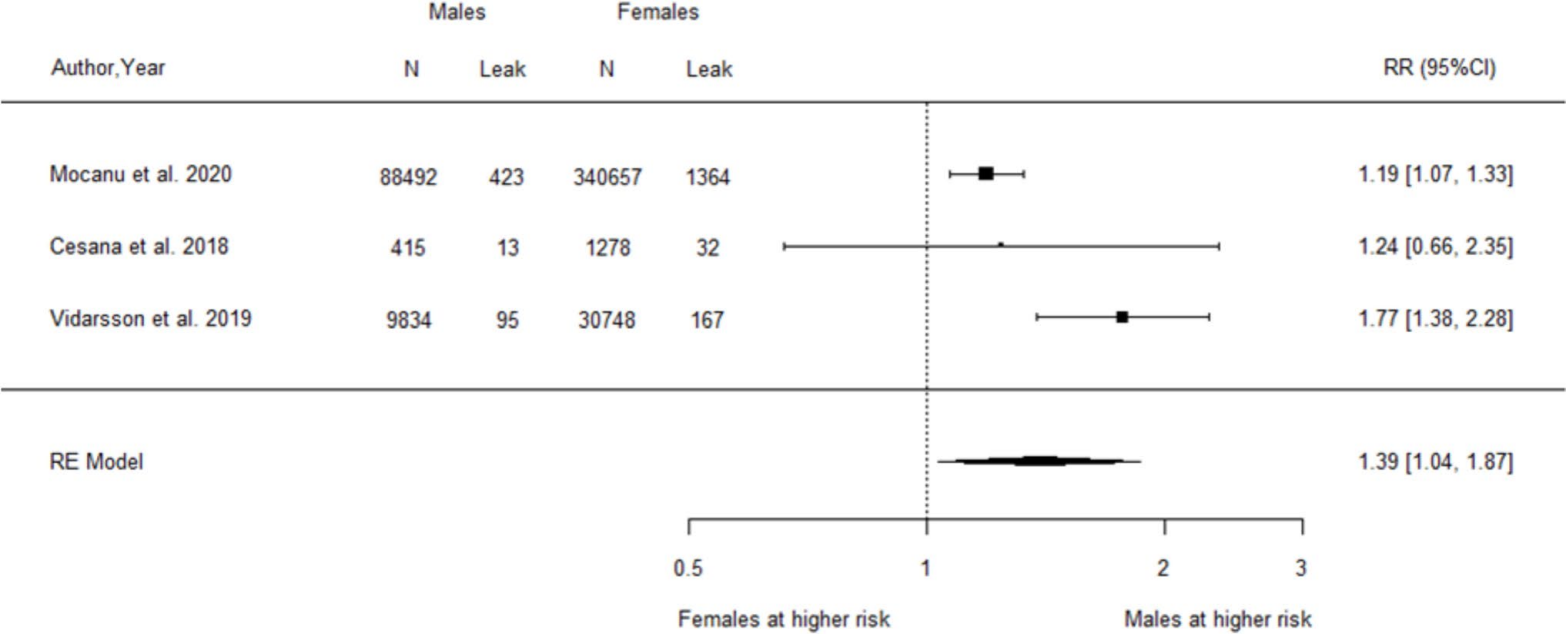
Forest plot of relative risk for postoperative anastomotic leak associated with male relative to female sex

For sex, the pooled analysis of three studies [39, 46, 72] demonstrated a significantly increased relative risk of postoperative anastomotic leak among males compared to females (RR = 1.39, 95% CI 1.04–1.87, Fig. 4). The analysis detected a high level of heterogeneity between studies (*I*^2^ = 72.36%, Q-statistic *p*-value = 0.018).

### Haemorrhage

**Fig. 5:**
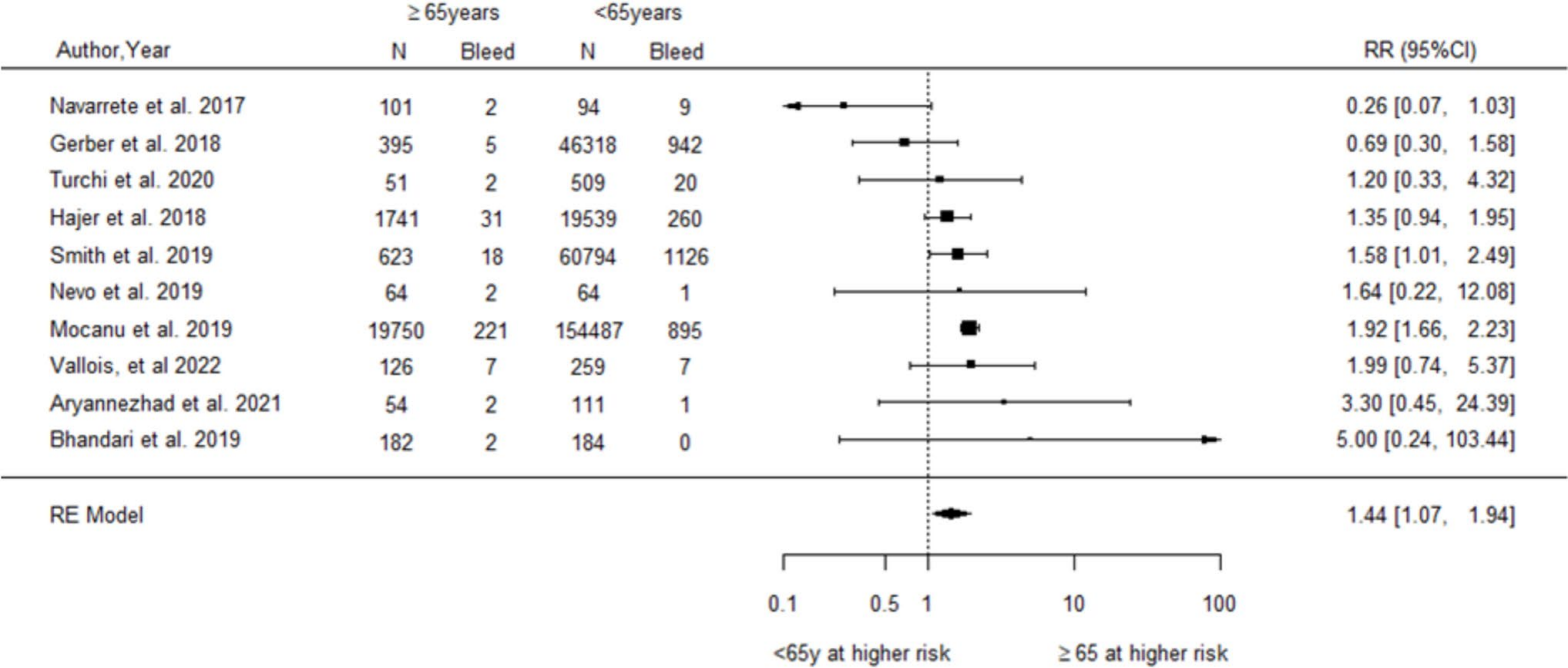
Forest plot of relative risk for postoperative haemorrhage associated with older relative to younger age

For age, the pooled analysis of ten studies [33, 37, 40, 51, 53, 64, 66, 75, 76, 82] demonstrated a significantly increased relative risk of postoperative haemorrhage among older compared to younger patients (RR = 1.44, 95% CI 1.07–1.94, Fig. 5). The analysis detected a moderate level of heterogeneity between studies (*I*^2^ = 45.3%, Q-statistic *p*-value = 0.046). Egger’s test detected no evidence of publication bias (*p* = 0.353). Sensitivity analyses excluding studies that adjusted results for comorbidities produced almost identical pooled results (RR = 1.46, 95% CI 1.07–1.98, Supplementary Section S6 Fig. S6.4). However, another analysis excluding studies with different age groupings showed no significant effect (RR = 0.94, 95% CI 0.39–2.26, Supplementary Section S6 Fig. S6.5).

**Fig. 6:**
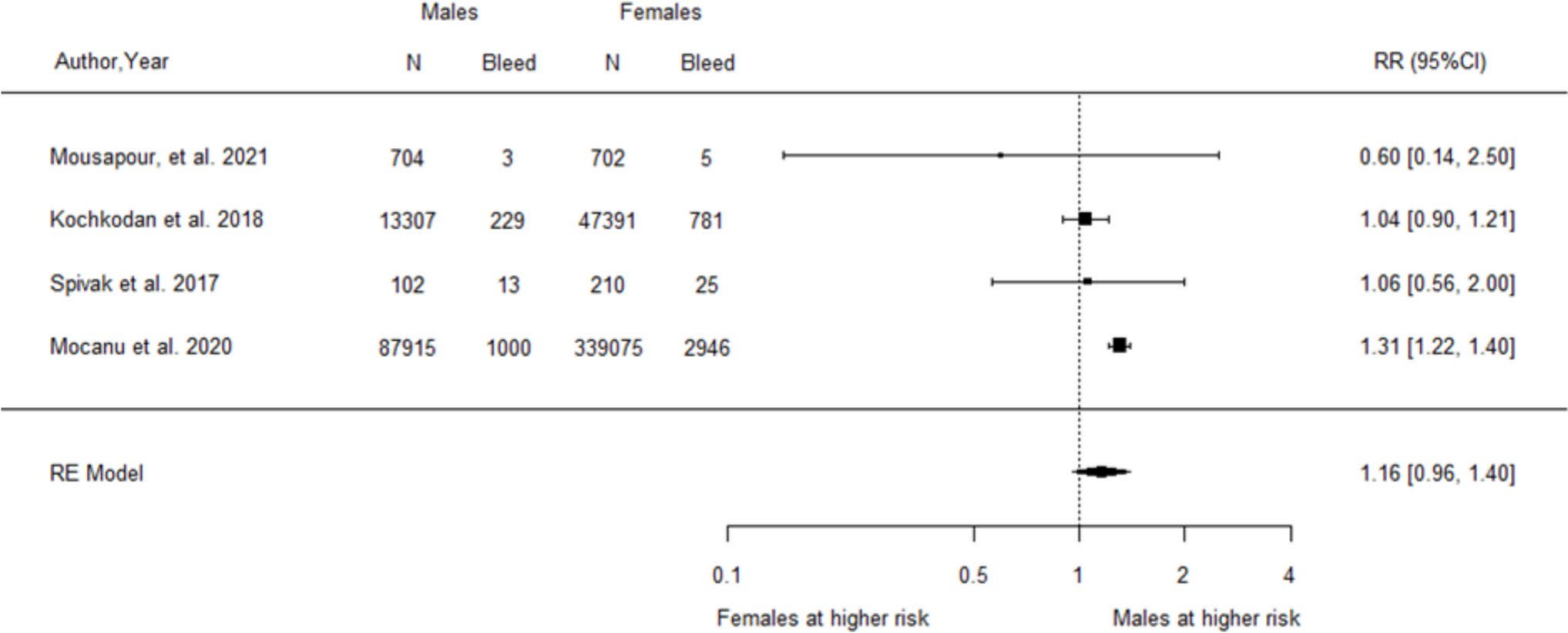
Forest plot of relative risk for postoperative haemorrhage associated with male relative to female sex

For sex, the pooled analysis of four studies [46, 59, 74, 92] demonstrated a non-significant trend to suggest an increased relative risk of postoperative haemorrhage among males compared to females (RR = 1.16, 95% CI 0.96–1.40, Fig. 6). The analysis detected a significant amount of heterogeneity between studies (*I*^2^ = 64.94%, Q-statistic *p*-value = 0.0356).

### Serious Complications

**Fig. 7:**
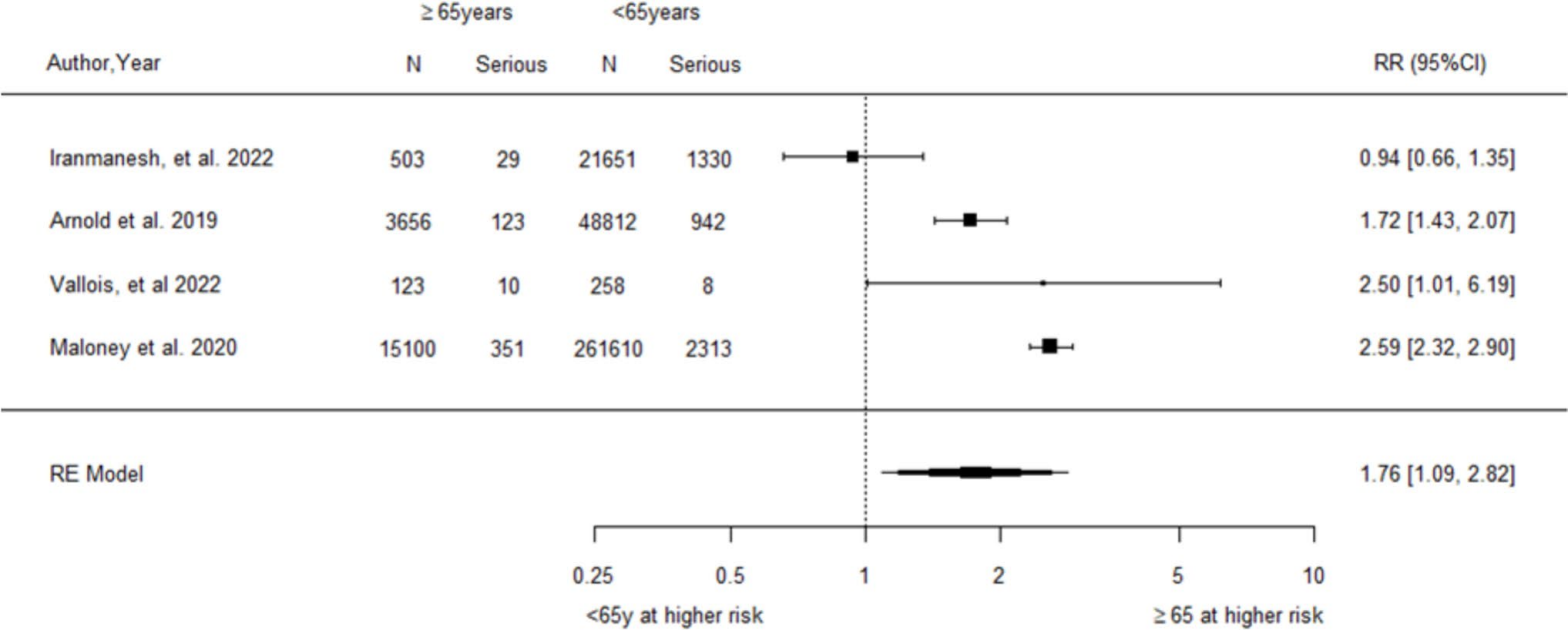
Forest plot of relative risk for postoperative serious complications associated with older relative to younger age

For age, the pooled analysis of four studies [28, 40, 47, 57] demonstrated a significantly increased relative risk of postoperative serious complications among older compared to younger patients (RR = 1.76, 95% CI 1.09–2.82, Fig. 7). The analysis detected a very high level of heterogeneity between studies (*I*^2^ = 93.24%, Q-statistic *p*-value < 0.001). A sensitivity analysis excluding the single study that adjusted results for comorbidities and with different age groupings produced almost identical pooled results (RR = 1.65, 95% CI 0.94–2.90, Supplementary Section S6 Fig. S6.6).

After excluding studies with overlapping results, there were insufficient studies available to pool results of serious complications by sex.

### Narrative Synthesis

Out of the 22 studies that examined race/ethnicity, 11 studies found that complication rates were higher among Black/African American patients compared to other races or ethnicities [42, 44, 46–50, 65, 69, 88, 89]. In contrast, four studies reported higher complication rates among White/Caucasian patients [68, 79, 91, 97]. An analysis of three studies revealed that Hispanic ethnicity was linked to a lower risk of complications [45, 47, 88], whereas complication rates were higher among other ethnic groups in one study [89] and lower in another study [46]. No association between race/ethnicity and complication rates was observed in six studies [52, 66, 67, 70, 73, 83]. Concerning heritage, one study found that first- or second-generation immigrants had an increased risk of complications [35].

Several studies explored the impact of various socioeconomic factors, including insurance status, index of multiple deprivation, disposable income, economic aid, education, and profession. Regarding insurance status, two studies observed an increased complication rate among patients covered by Medicare (USA) [32, 60], while one study reported a similar association with Medicaid (USA) [75]. Conversely, two studies found a decreased complication rate among self-pay patients [32, 60], while one study reported no association with insurance status [31]. Additionally, one study found no association between index of multiple deprivation, a measure of socioeconomic status in the UK, and postoperative complications [79]. Another study reported an increased complication rate among patients with lower disposable income and those receiving disability pension or social assistance, while no association was observed with education or profession [35].

The remaining demographic factors investigated were marital status and environmental factors such as geographical location and residence. One study found that higher complication rates were associated with being divorced/widow/widower, while lower rates were observed in individuals who were single [35]. Another study reported that complication rates increased with at least one social determinant of health [41]. These factors included economic (employment status, poor occupational environment, food insecurity, housing instability, financial hardship), education (early childhood development issues, inadequate education and literacy), social (cultural, race, incarceration, legal, psychosocial issues), healthcare (unavailability, inaccessibility, healthcare literacy), and environmental (exposure to natural disaster, lead or mould exposure, safety) factors [41]. Of the two studies analysing geographical location, both reported no association [56, 79]. Complication rates were lower among patients living in a medium or small town in one study [35].

Regarding race/ethnicity, three studies found an increased mortality risk among Black/African American patients [46, 47, 52], while one study found no such association with race/ethnicity [45].

## Discussion

This systematic review and meta-analysis provide compelling evidence that specific demographic factors are associated with an increased risk of postoperative complications following primary bariatric surgery. Our findings highlight the importance of considering these factors when evaluating patients for bariatric surgery and developing tailored perioperative care plans. This may involve intensified monitoring and enhanced multidisciplinary team postoperative support, including pain management, nutritional counselling, and psychosocial support, to minimise complications and improve patient outcomes [99].

Older age was consistently identified as a significant risk factor for postoperative mortality, serious complications, anastomotic leak, and haemorrhage. This is in line with some previous research and recommendations in clinical practice guidelines for bariatric surgery [5, 100], while other studies presented contrasting results [101]. Older patients are more likely to have underlying health conditions such as cardiovascular disease, diabetes mellitus, and respiratory disorders [40]. These comorbidities can increase the risk of complications during and after surgery [40]. As people age, their body’s ability to cope with stress and recover from surgery may decline [102]. This reduced physiological reserve can increase the risk of complications, including mortality [103]. Older patients may require more complex surgical procedures or have longer operative times, which can increase the risk of complications [104]. Older patients may have a slower recovery time and may be more prone to complications such as infections, wound healing problems, and blood clots [103]. These factors can interact with each other to increase the overall risk of postoperative complications in older patients undergoing bariatric surgery. Clinicians should carefully balance the increased risk of complications for older individuals with the potential health benefits [105] and relatively low absolute rates of deaths and major complications associated with bariatric surgery [5, 106].

Male sex was associated with an increased risk of anastomotic leak, suggesting that men may be at higher risk for certain surgical complications. Men may have anatomical variations in the gastrointestinal tract that could influence the healing process and increase the risk of anastomotic leak. For example, differences in the length and diameter of the gastrointestinal tract or the angle of the anastomosis could impact healing [107]. Testosterone, the primary male sex hormone, has been implicated in various physiological processes, including wound healing [108]. It is possible that testosterone may influence the healing of bariatric surgery anastomoses. Men are more likely to engage in certain behaviours, such as smoking and excessive alcohol consumption [109, 110], which can negatively impact wound healing and increase the risk of complications [111, 112]. Further research is needed to elucidate the specific mechanisms underlying the increased risk of anastomotic leak in male patients undergoing bariatric surgery. This knowledge could inform the development of targeted interventions to reduce the risk of this complication in men.

Socioeconomic factors such as Black/African American race, low financial status, and marital status were also linked to higher complication risks in some studies. Black/African Americans have experienced historical and systemic inequities in healthcare access and quality [113], which can contribute to disparities in health outcomes. They may also be more likely to have pre-existing health conditions such as diabetes, hypertension, and obesity [113], which can increase the risk of complications during and after surgery. Black/African Americans may receive lower-quality surgical care, including less experienced surgeons or inadequate postoperative care [114], which could increase the risk of complications.

Individuals with low financial status may have limited access to preventive care, specialised care, and necessary medications, which can increase the risk of complications [35]. Lower-income individuals may have limited or no health insurance coverage, which can hinder access to necessary care, especially in countries like the USA [32]. Individuals with low social support, such as those who are divorced, widowed, or single, may have limited emotional and practical support during recovery [115], which could increase the risk of complications. These findings underscore the importance of addressing social determinants of health and ensuring equitable access to healthcare for all patients undergoing bariatric surgery.

While this review provides valuable insights, it is important to acknowledge its limitations. The inclusion of only observational studies published between 2017 and 2022 from a single electronic database may have introduced potential biases, and the generalizability of the findings may be limited to the included populations. Further research is needed to better understand regional variations and the impact of socioeconomic factors in specific countries. For studies reporting more than five complications, we extracted results for only the five most common complications, regardless of their statistical significance, potentially missing rarer but significant complications. The included studies showed substantial heterogeneity in terms of bariatric surgery types, sample sizes, countries, and follow-up durations. Additionally, the definition of postoperative complications varied across studies, with some including mortality within the definition while others reported it separately. Furthermore, the focus on demographic factors excluded other potentially important predictors of postoperative complications, such as clinical and behavioural factors. Finally, another limitation of this study is the absence of American Society of Anaesthesiologists (ASA) physical status classification data [116]. Given the possible ASA score’s relevance in assessing patient health and its potential impact on postoperative complications from bariatric surgery [117], future studies should incorporate this variable for a more comprehensive risk assessment.

Future research should explore the specific mechanisms underlying the associations between demographic factors and postoperative complications. A synthesis of studies investigating the impact of clinical and behavioural factors, such as preoperative BMI, comorbidities, smoking, alcohol use, and functional status, is needed to provide a more comprehensive understanding of the risk factors for postoperative complications following bariatric surgery.

## Conclusion

In conclusion, older age was consistently associated with an increased risk of postoperative mortality, serious complications, anastomotic leak, and haemorrhage following primary bariatric surgery. Additionally, male sex, Black/African American race, low financial status, and marital status were linked to a higher risk of postoperative complications in some studies. These findings highlight the importance of considering patient demographics and socioeconomic factors when assessing the risks and benefits of bariatric surgery. Further research is needed to explore complication risks across bariatric surgery types and expand the assessment of patient factors to include clinical and behavioural variables.

## Blinded Summary Statement

## Supplementary Information

Below is the link to the electronic supplementary material.

Supplementary file1 (DOCX 1779 KB)

## Data Availability

No datasets were generated or analysed during the current study.
