# Supplementary material for: Demographic Factors Associated with Postoperative Complications in Primary Bariatric Surgery: A Rapid Review
Source: Obes Surg. 2025 Mar 13;35(4):1456–68. doi: 10.1007/s11695-025-07784-x (PMC11976351; doi:10.1007/s11695-025-07784-x)
Supplement: Supplementary file 1 — Supplementary file1 (DOCX 1779 KB) [file 11695_2025_7784_MOESM1_ESM.docx]

**Supplementary File**

**Table of contents**

[Supplementary Table S1: Search strategy 2](#_Toc188393283)

[Supplementary Table S2: Definitions of reported complications 4](#_Toc188393284)

[Supplementary Section S3: List of excluded studies with reasons 6](#_Toc188393285)

[Supplementary Section S4: Summary and characteristics of quantitative studies reviewed 7](#_Toc188393286)

[Supplementary Table S5: Risk of bias assessment of studies reviewed 81](#_Toc188393287)

[Supplementary Section S6: Sensitivity analyses 86](#_Toc188393288)

[S6.1: Mortality as a postoperative complication: sensitivity analysis excluding studies with different age groupings 86](#_Toc188393289)

[S6.2: Anastomotic leak as a postoperative complication: sensitivity analysis excluding studies adjusted for comorbidities 86](#_Toc188393290)

[S6.3: Anastomotic leak as a postoperative complication: sensitivity analysis excluding studies with different age groupings 87](#_Toc188393291)

[S6.4: Haemorrhage as a postoperative complication: sensitivity analysis excluding studies adjusted for comorbidities 88](#_Toc188393292)

[S6.5: Haemorrhage as a postoperative complication: sensitivity analysis excluding studies with different age groupings 89](#_Toc188393293)

[S6.6: Serious complications as a postoperative complication: sensitivity analysis excluding one study adjusted for comorbidities and with different age groupings 89](#_Toc188393294)

[Supplementary Section S7: References 91](#_Toc188393295)

# Supplementary Table S1: Search strategy

Ovid MEDLINE

| **#** | **Query** | **Results to 26^th^ July 2022** |
| --- | --- | --- |
| 1 | bariatric surgery/ or gastric bypass/ | 24,150 |
| 2 | (((bariatric or metabolic or stomach or weight-loss) adj1 (surger* or stapl* or operat*)) or gastroenterostomy or gastric bypass or sleeve gastrectomy or gastric band*).ti,ab. | 34,616 |
| 3 | 1 or 2 | 37,860 |
| 4 | postoperative complications/ or anastomotic leak/ or postoperative hemorrhage/ or venous thromboembolism/ or intestinal obstruction/ or cholelithiasis/ or cholecystolithiasis/ or choledocholithiasis/ or gallstones/ or peptic ulcer/ or incisional hernia/ | 508,577 |
| 5 | (postoperative complication* or (anastomotic adj2 (leak* or stenos?s or stricture*)) or h?emorrhage* or bleed* or venous thromboembol* or ((intestinal or bowel) adj2 obstruction*) or cholelithiasis or cholecystolithiasis or choledocholithiasis or gallstone* or ((marginal or stomal or peptic) adj2 ulcer*) or ((incisional or postoperative or internal) adj2 hernia*)).ti,ab. | 588,047 |
| 6 | 4 or 5 | 968,619 |
| 7 | 3 and 6 | 9,029 |
| 8 | limit 7 to yr="2012 -Current" | 5,591 |
| 9 | limit 7 to yr="2017 -Current" | 3,323 |
| 10 | limit 9 to (english language and humans and "all adult (19 plus years)") | 1,841 |

**NOTES:** Two papers (not retrieved in any of the database searches) were identified by handsearching the bibliographies of full-length articles, and one paper (not retrieved in any of the database searches) was identified by internet searching.

1. Husain F, Jeong IH, Spight D, Wolfe B, Mattar SG. Risk factors for early postoperative complications after bariatric surgery. Ann Surg Treat Res. 2018;95(2):100-10.
2. Pechman DM, Flores FM, Kinkhabwala CM, Salas R, Berk RH, Weithorn D, Camacho DR. Bariatric surgery in the elderly: outcomes analysis of patients over 70 using the ACS-NSQIP database. Surg Obes Relat Dis. 2019;15(11):1923-32.
3. Montgomery JR, Cohen JA, Brown CS, Sheetz KH, Chao GF, Waits SA, Telem DA. Perioperative risks of bariatric surgery among patients with and without history of solid organ transplant. Am J Transplant. 2020;20(9):2530-9

# Supplementary Table S2: Definitions of reported complications

| **Complications** | **Definitions** |
| --- | --- |
| Mortality | - 30-day mortality - 30-day mortality LRYGB - 30-day mortality LSG - 30-day mortality RYGB - Death LRYGB - Death LSG - Early mortality - Mortality - Death - Death during operation (intra-operative death) or post-operative death within 30 days of procedure - Death related to surgery |
| Anastomotic leak | - Anastomotic leak - Anastomotic/staple-line leak LRYGB - Anastomotic/staple-line leak LSG - Gastrointestinal leak - Leak - Leak at the GJ - Leak or perforation SG - Leakage - Proximal leakage - Sleeve or anastomotic leak - Staple line leakage |
| Haemorrhage | - 30-day bleeding - Bleed - Bleed after LSG - Bleeding and reoperation - Bleeding/haematoma - Blood loss requiring transfusion 30-day LRYGB - Blood loss requiring transfusion 30-day LSG - Haemorrhage - Haemorrhage RYGB - Haemorrhage SG - In-hospital bleeding - Intra-abdominal bleeding - Postoperative bleeding - Postoperative haemorrhage - Required transfusion LRYGB - Required transfusion LSG - Upper gastrointestinal bleeding |
| Serious complications | - Clavien-Dindo III-V - Major complication - Major complication LSG - Major complication RYGB - Major postoperative complications - SAE - Serious complication - Serious complication SG - Serious complications - Serious morbidity LRYGB - Serious morbidity LSG - Serious postoperative complication - Severe complication RYGB |
| Any complications | - 30-day complication - Any complication - Any complication LRYGB - Any complication LSG - Any complication RYGB - Any complication SG - Any complication without death LRYGB - Any complication without death LSG - Any complications - any complications - LAGB - any complications - RYGB - Any medical complications - At least one post-op complication - complications - Early postoperative complications - overall - Overall Postoperative complications - Overall 4-year complications - Post-surgery complication - postoperative complications - Postoperative complications - Surgical complications - Total general complications |

# Supplementary Section S3: List of excluded studies with reasons

- Did not meet eligibility criteria for population and setting [1-13]
- Did not meet eligibility criteria for patient factor [14-83]
- Did not meet eligibility criteria for outcome [84, 85]
- Did not meet eligibility criteria for study design [86, 87]

# Supplementary Section S4: Summary and characteristics of quantitative studies reviewed

A total of 71 studies published between 2017 and 2022 were included in this review, comprising 69 cohort studies[29-97] and 2 case-control studies.[98, 99] Of these studies, 10 studies used data collections combining both the United States (US) and Canada,[33, 44, 47, 49, 61, 62, 64, 67, 69, 70] 27 were conducted on US data only,[31, 35, 37, 38, 40, 42, 45, 46, 48, 51, 52, 56, 57, 66, 71, 73, 75, 78, 81, 82, 84-86, 91, 92, 97, 99] four were conducted on Canadian data only,[32, 36, 68, 83] five studies were conducted in Sweden,[53, 55, 74, 88, 89] three were conducted in Israel,[60, 93, 94] two studies were conducted in each of England,[30, 76] Iran,[34, 39] Spain,[43, 95] Germany,[59, 87] Poland,[79, 96] Turkey,[50, 58] Chile,[63, 65] and one study was conducted in each of France,[29] Argentina,[41] India,[72] Switzerland,[77] New Zealand,[80] the Netherlands,[98] China,[54] and Italy.[90] A number of studies reported analyses on the same source data source, such as Metabolic and Bariatric Surgery Accreditation and Quality Improvement (MBSAQIP) from the US and Canada analysed in 22 studies,[33, 35, 38, 40, 42, 44-49, 57, 61, 62, 64, 66, 67, 69-71, 86, 92] Scandinavian Obesity Surgery Register (SOReg) analysed in 5 studies,[53, 55, 74, 88, 89] Ontario Bariatric Register analysed in 4 studies,[32, 36, 68, 83] American College of Surgeon National Surgical Quality Improvement Program (ACS-NSQIP) analysed in 3 studies,[37, 73, 81] Michigan Bariatric Surgery Collective (MBSC) analysed in 3 studies,[51, 56, 82] and single institution data from Barcelona analysed in 2 studies [43, 95] and Santiago analysed in 2 studies.[63, 65] All 71 studies were in clinical populations, with sample sizes ranging from 100,000 to 500,000 in 18 studies,[35, 40, 42, 44, 45, 47-49, 57, 61, 64, 67, 69, 70, 84-86, 92] 40,000 to 80,000 in 10 studies,[33, 53, 55, 56, 62, 71, 73, 81, 82, 88] 12,000 to 40,000 in 10 studies,[31, 32, 37, 46, 51, 66, 74, 76, 87, 91] 1,000 to 8,000 in 12 studies,[29, 30, 34, 36, 38, 52, 68, 78, 80, 83, 89, 90] 200 to 800 in 11 studies,[41, 43, 63, 72, 77, 79, 94-98] and 40 to 200 in 10 studies.[39, 50, 54, 58-60, 65, 75, 93, 99] The studies explored various types of bariatric surgical procedures, including laparoscopic sleeve gastrectomy (LSG) in 55 studies,[29-35, 37-40, 42-51, 54, 56-61, 63-70, 72, 73, 75, 76, 79, 81-84, 86, 87, 90-96, 99] Roux-en-Y gastric bypass (RYGB) in 54 studies,[29, 30, 32-42, 44-49, 51-53, 56, 57, 59, 62-74, 76-84, 86, 88, 89, 91, 92, 96, 98] one anastomosis gastric bypass in four studies,[30, 34, 39, 72] biliopancreatic diversion with duodenal switch in three studies,[40, 82, 97] laparoscopic adjustable gastric banding (LAGB) in seven studies,[46, 51, 52, 55, 76, 82, 91] sleeve gastrectomy with jejunal bypass in two studies,[63, 65] and other or unspecified techniques in three studies.[30, 76, 85]

A total of 58 studies examined age as an exposure variable,[29, 30, 32, 35, 36, 38-41, 43-47, 50, 53, 54, 56, 58-67, 69-81, 83-99] 35 studies examined sex,[34-37, 40, 44, 45, 47, 48, 50, 53, 54, 58, 59, 61, 62, 64, 65, 67, 69-71, 75-77, 82, 84, 85, 90-92, 94, 96, 98, 99] 22 studies examined race/ethnicity,[33, 35, 40, 42, 44-47, 49, 51, 57, 61, 62, 64, 69-71, 75, 76, 91, 92, 98] three studies examined insurance status,[52, 84, 85] and two studies examined geographical location.[68, 76] Additionally, one study each examined residence,[55] education,[55] profession,[55] disposable income,[55] marital status,[55] economic aid,[55] heritage,[55] index of multiple deprivation,[76] and general social determinants of health.[31] The specific complications investigated varied across the studies. Most studies reported on a mix of multiple postoperative complications, including but not limited to gastrointestinal, infectious, haemorrhagic, cardiovascular, respiratory, and renal complications. However, some studies investigated specific individual rarer complications, such as acute pancreatitis,[84] sepsis,[35] marginal ulcer,[36, 71] gallstones and/or subsequent cholecystectomy,[50, 58, 65, 91, 98] deep vein thrombosis and pulmonary embolism,[47, 70, 97] leak,[53, 62, 90, 92] hypoglycaemia and early dumping syndrome,[54] haemorrhage,[61, 94] dehydration,[64] Clostridium difficile infection,[69] strictures,[74] thiamine deficiency,[75] portomesenteric vein thrombosis,[99] and anaemia.[78] 30 studies reported complications occurring within 30 days after surgery.[30, 33, 37, 40-44, 46, 48, 49, 51, 52, 55-57, 64, 66-71, 77, 82, 86, 88, 92, 95, 96] One study reported complications within 60 days,[31] three studies reported complications within 90 days,[29, 94, 97] one study reported complications within six months,[84] four studies reported complications within one year,[58, 65, 74, 75] one study reported complications within two years,[98] one study reported complications within four years,[38] one study reported complications within eight years,[78] and 29 studies reported complications with an unspecified timeframe.[32, 34-36, 39, 45, 47, 50, 53, 54, 59-63, 72, 73, 76, 79-81, 83, 85, 87, 89-91, 93, 99] Mortality was included in the definition of postoperative complications in 10 studies,[31, 40, 43, 49, 51, 52, 56, 68, 72, 77] while 19 studies reported mortality as a separate outcome.[30, 32, 33, 37, 39, 42, 44, 45, 48, 57, 63, 66, 73, 80, 81, 86-88, 95]

Table S4: Summary and characteristics of quantitative studies reviewed

| **Study details** | **Participant details** | **Patient factors (independent variables)** | **Outcome(s) – postoperative complications** | **Statistical methods, result/effect estimates** | **Author conclusions and reviewer’s comments** |
| --- | --- | --- | --- | --- | --- |
| **Authors:**  Vallois, et al. [88]  **Year published:** 2022  **Study design:**  Cohort study  **Country:**  France | **Sample size:**  N=1,681  **Inclusion criteria:**   - All patients with morbid obesity who underwent laparoscopic bariatric surgery (laparoscopic sleeve gastrectomy (LSG), laparoscopic Roux-en-Y gastric bypass (LRYGB)) from October 2005 to April 2019 at two French tertiary referral bariatric centres - People with a follow-up longer than 12 months after bariatric surgery   **Exclusion criteria:**   - Nil   **Setting and population:**   - Patient records identified from a prospectively maintained database from two French tertiary referral bariatric centres | - Age | 1. Postoperative complications group and separately (occurring within 90 days after surgery) (severe postoperative complications, overall morbidity, leakage, haemorrhage, infectious complications, reoperation) | **Statistical analysis:**   - Descriptive statistics - Propensity Score Matching (PSM) used to compare complication rates between age ≥60y (n=133) and age <60y (n=266) groups - Paired population was analysed using a generalized linear model “with an appropriate distribution for serious complications.”   **Results/effect estimates:**   1. Postoperative complications   Overall morbidity (%)   - Age ≥60y: 31.6% (42/133) - Age <60y: 22.9% (61/266) - P = 0.044   Leakage (%)   - Age ≥60y: 5.3% (7/133) - Age <60y: 1.1% (3/266) - P = 0.026   Serious complications (%)   - Age ≥60y: 7.5% (10/133) - Age <60y: 3.0% (8/266) - P = 0.053   Infectious complications (%)   - Age ≥60y: 10.6% (14/133) - Age <60y: 7.2% (19/266) - P = 0.187   Haemorrhage (%)   - Age ≥60y: 5.3% (7/133) - Age <60y: 2.6% (7/266) - P = 0.195 | **Author’s conclusion:**  “This propensity-matched study suggests that laparoscopic bariatric surgery is an effective treatment in obese elderly patients (EPs) in terms of weight loss and resolution of comorbidities. However, the EP should be warned of the increased risk of severe postoperative complications within 90 days, including leakage and reoperation rates, especially after RYGB.”  **Reviewer’s comments:**  This study shows that older age is significantly associated with increased rates of overall morbidity, and leakage. Although not statistically significant, rates of serious complications, infectious complications, and haemorrhage tended to be higher among older patients.  This study has clearly met 7/10 (70%) criteria in the critical appraisal tool. |
| **Authors:**  Singhal, et al. [89]  **Year published:** 2022  **Study design:**  Cohort study  **Country:**  England | **Sample size:**  N=7,092  **Inclusion criteria:**   - All adult (≥18y) patients undergoing elective primary bariatric surgery (LSG, Roux-en-Y gastric bypass (RYGB), one anastomosis gastric bypass (OAGB), others) between 1^st^ May and 31^st^ October 2020, regardless of the surgical approach or the patient’s preoperative COVID-19 status   **Exclusion criteria:**   - Nil   **Setting and population:**   - “Global, multicentre” | - Age | 1. Postoperative complications group (within 30 days) 2. Mortality (within 30 days) | **Statistical analysis:**   - Descriptive statistics - Chi-square test used to compare complication rates between patient groups age ≥65y and age <65y   **Results/effect estimates:**  1. Postoperative complications (%)   - Age ≥65y: 11.4% (17/149) - Age <65y: 6.6% (460/6,923) - P = 0.022   2. Mortality (%)   - Age ≥65y: 0.7% (1/149) - Age <65y: 0.1% (8/6,923) - P = 0.17 | **Author’s conclusion:**  “Bariatric surgery during the COVID-19 pandemic is associated with a higher complication rate in those ≥65 years of age compared to those <65 years old.”  **Reviewer’s comments:**  This study shows that older age is significantly associated with increased rates of postoperative complications. Although not statistically significant, mortality rates tended to be higher among older patients.  This study has clearly met 6/9 (67%) criteria in the critical appraisal tool. |
| **Authors:**  Khalid, et al. [90]  **Year published:** 2022  **Study design:**  Cohort study  **Country:**  United States | **Sample size:**  N=16,736  **Inclusion criteria:**   - Patients who underwent vertical sleeve gastrectomy (VSG) in the United States from January 1, 2010 through June 30, 2018   **Exclusion criteria:**   - Nil   **Setting and population:**   - Patient records identified from MARINER-15, an all-payer claims database from January 1, 2010 through June 30, 2018 in the United States of America | - Social determinants of health (SDH) (economic: employment status, poor occupational environment, food insecurity, housing instability, financial hardship; education: early childhood development issues, inadequate education and literacy; social: cultural, race, incarceration, legal, psychosocial issues; healthcare: unavailability, inaccessibility, healthcare literacy; environmental: exposure to natural disaster, lead or mould exposure, safety) | 1. Postoperative complications group and separately (cardiac complications, acute kidney injury (AKI), deep vein thrombosis (DVT), wound complication, haematoma, pneumonia, pulmonary embolism (PE), transfusion, or urinary tract infection (UTI) within 60 days; 30- and 90-day all-cause mortality; all-cause readmission within 30 or 90 days) | **Statistical analysis:**   - Descriptive statistics - Hazard Ratios (HR) for complications associated with history of at least one SDH (i.e. disadvantage) compared with none - “The total population was exactly matched in a 1:1 fashion based on” covariates   **Results/effect estimates:**  1. Postoperative complications  Any complication (%)   - ≥1 SDH: 9.48% (793/8,368) - No SDH: 3.59% (300/8,368) - P <0.001 - HR = 1.20 (95% CI: 1.03-1.40, p = 0.02)   AKI (%)   - ≥1 SDH: 1.35% (113/8,368) - No SDH: 0.75% (63/8,368) - P <0.001 - HR = 0.74 (95% CI: 0.51-1.08, p = 0.12)   Haematoma (%)   - ≥1 SDH: 0.53% (44/8,368) - No SDH: 0.44% (37/8,368) - P <0.001 - HR = 0.78 (95% CI: 0.48-1.26, p = 0.31)   Pneumonia (%)   - ≥1 SDH: 1.25% (105/8,368) - No SDH: 0.48% (40/8,368) - P <0.001 - HR = 1.39 (95% CI: 0.94-2.09, p = 0.11)   UTI (%)   - ≥1 SDH: 6.02% (504/8,368) - No SDH: 1.73% (145/8,368) - P <0.001 - HR = 1.37 (95% CI: 1.11-1.70, p = 0.004) | **Author’s conclusion:**  “Patients undergoing VSG who had at least 1 type of SDH had increased rates of any complication, cardiac complications, acute kidney injury, deep vein thrombosis, wound complication, pneumonia, transfusion and urinary tract infection, but they were also less likely to return for readmission at 30 and 90 days.”  **Reviewer’s comments:**  This study shows that the presence of at least one SDH is significantly associated with increased risk of any complication, and UTI. Although the hazard ratios were not statistically significant, rates of AKI, haematoma, and pneumonia tended to be higher among patients with at least one SDH.  This study has clearly met 7/9 (78%) criteria in the critical appraisal tool. |
| **Authors:**  Iranmanesh, et al. [91]  **Year published:** 2022  **Study design:**  Cohort study  **Country:**  Canada | **Sample size:**  N=23,513  **Inclusion criteria:**   - Adult patients (>18y) undergoing primary RYGB or SG in accredited Bariatric Centres of Excellence in Ontario, Canada over the period from January 2010 to August 2019 - Patients who had a minimum follow-up of 3 months after surgery   **Exclusion criteria:**   - Patients undergoing other bariatric procedures (i.e. adjustable gastric band, duodenal switch) - Patients undergoing reoperative bariatric surgery, including revisions, conversions and reversals of any previous bariatric procedure - Patients who refused to participate in the Ontario Bariatric Registry   **Setting and population:**   - Patient records identified from the Ontario Bariatric Registry | - Age | 1. Postoperative complications group and separately (from surgery up to last completed follow-up visit) (overall, early (<30 days after surgery), gastrointestinal leaks, internal hernia, anastomotic stricture, gastrojejunal ulcer, bowel obstruction, surgical site infection (SSI), thrombotic event (DVT/PE), bleeding, nutritional deficiency) 2. 30-day mortality | **Statistical analysis:**   - Descriptive statistics - “Primary and secondary outcomes were compared between patients <65y and ≥65y using Fisher’s exact and Mann-Whitney tests where appropriate.”   **Results/effect estimates:**  1. Postoperative complications  Overall complications (%)   - Age ≥65y: 13.7% (73/532) - Age <65y: 14.7% (3,388/22,981) - P = 0.537   Overall major complications (%)   - Age ≥65y: 5.5% (29/532) - Age <65y: 5.8% (1,330/22,981) - P = 0.850   Gastrojejunal ulcer (%)   - Age ≥65y: 4.7% (15/532) - Age <65y: 3.6% (700/22,981) - P = 0.287   Internal hernia (%)   - Age ≥65y: 3.4% (11/532) - Age <65y: 2.5% (488/22,981) - P = 0.276   Nutritional deficiency (%)   - Age ≥65y: 2.3% (12/532) - Age <65y: 2.4% (558/22,981) - P = 0.999   2. Mortality   - Age ≥65y: 0% (0/532) - Age <65y: 0% (10/22,981) - P = 0.999 | **Author’s conclusion:**  “Patients <65 and ≥65 years had similar perioperative morbidity and mortality after bariatric surgery. Even though patients <65 years had overall better medium-term outcomes, bariatric surgery is safe and yields significant weight loss and comorbidities improvement in patients ≥65 years.”  **Reviewer’s comments:**  This study shows that older age is not associated with increased rates of postoperative complications, or mortality.  This study has clearly met 5/9 (56%) criteria in the critical appraisal tool. |
| **Authors:**  Nafiu, et al. [92]  **Year published:** 2021  **Study design:**  Cohort study  **Country:**  United States, Canada | **Sample size:**  N=44,090  **Inclusion criteria:**   - Adult patients (>18y) who underwent bariatric surgery (SG, RYGB) between 2015 and 2018 in bariatric surgical institutions in the United States and Canada - Patients who were assigned an American Society of Anaesthesiologists (ASA) physical status classification of 1 or 2   **Exclusion criteria:**   - Patients who had revision/conversion - Patients who had a history of prior bariatric surgery - Patients who had a body mass index (BMI) <25 kg/m^2^ - Patients who were listed as emergent cases - Patients who were missing information on race - Patients who were not of either black or white race   **Setting and population:**   - Patient records identified from the Metabolic and Bariatric Surgery Accreditation and Quality Improvement (MBSAQIP) Participant Use File (PUF) | - Race | 1. 30-day composite complications group (occurrence of 1 of 7 postoperative events: wound complications, pulmonary complications, renal complications, neurologic complications, cardiac complications, sepsis, bleeding or unplanned reoperation) 2. 30-day mortality | **Statistical analysis:**   - Descriptive statistics - Propensity score-adjusted logistic regression models used “to estimate the odds ratios (OR) and 95% confidence intervals (CIs) for the strength of the association between race and our independent variables”   **Results/effect estimates:**  1. Postoperative complications (%)   - Non-Hispanic black: 0.7% (149/22,045) - Non-Hispanic white: 0.4% (87/22,045) - OR = 1.72 (95% CI: 1.32-2.24, p <0.01)   2. Mortality   - Non-Hispanic black: 0.1% (11/22,045) - Non-Hispanic white: 0.0% (4/22,045) - OR = 2.75 (95% CI: 0.88-8.64, p = 0.08) | **Author’s conclusion:**  “Even among relatively healthy patients, race appears to be an important determinant of post-bariatric surgery complications and resource utilization. Research and interventions aimed at narrowing the racial disparities in bariatric surgery outcomes may need to broaden the focus beyond the racial variation in the preoperative co-morbidity burden.”  **Reviewer’s comments:**  This study shows that black race is significantly associated with increased risk of postoperative complications. Although not statistically significant, mortality rates tended to be higher among black patients.  This study has clearly met 7/9 (78%) criteria in the critical appraisal tool. |
| **Authors:**  Mousapour, et al. [93]  **Year published:** 2021  **Study design:**  Cohort study  **Country:**  Iran | **Sample size:**  N=1,414  **Inclusion criteria:**   - Patients who underwent primary laparoscopic bariatric surgery (SG, RYGB, OAGB) from March 2013 to March 2017 in the Tehran Obesity Treatment Centre - Patients who had completed 36-months follow-up - Patients aged 18-65 years with BMI ≥40 kg/m^2^ or between 35 and 40 kg/m^2^ in the presence of at least one obesity-related comorbidity   **Exclusion criteria:**   - Nil   **Setting and population:**   - Patients from the Tehran Obesity Treatment Centre | - Sex | 1. Postoperative complications group and separately (in-hospital: vomiting, bleeding, hospital-acquired pneumonia, other infection, PE, abscess due to leak; 30-day: vomiting, marginal ulcer, bleeding, other infection, intestinal obstruction, gastroesophageal reflux, bleeding, abscess due to leak, peritonitis, other; late: bleeding, other, intestinal obstruction, gastroesophageal reflux, abscess due to leak) | **Statistical analysis:**   - Descriptive statistics - “Fisher’s exact test was used for the comparison of postoperative complications”   **Results/effect estimates:**  1. Postoperative complications  In-hospital bleeding   - Women: 0.85% (6/707) - Men: 1.13% (8/707)   In-hospital abscess due to leak   - Women: 0.28% (2/707) - Men: 0.57% (4/707)   30-day vomiting   - Women: 0.42% (3/707) - Men: 0.28% (2/707)   30-day bleeding   - Women: 0.71% (5/707) - Men: 0.42% (3/707)   30-day abscess due to leak   - Women: 0.42% (3/707) - Men: 0.71% (5/707) | **Author’s conclusion:**  “Our matched‑pair cohort analysis demonstrated that bariatric surgery results in comparable short‑ and mid‑term efficacy in men and women, and is associated with similar rate and severity of postoperative complications between sexes. These findings suggest bariatric surgeons not to consider sex for patient selection in bariatric surgery.”  **Reviewer’s comments:**  This study shows that there is no significant difference in rates of postoperative complications between sexes.  This study has clearly met 8/9 (89%) criteria in the critical appraisal tool. |
| **Authors:**  Hui, et al. [94]  **Year published:** 2021  **Study design:**  Cohort study  **Country:**  United States | **Sample size:**  N=438,752  **Inclusion criteria:**   - Patients undergoing elective SG and RYGB   **Exclusion criteria:**   - Patients undergoing revisional, endoscopic, and uncommon or investigational procedures   **Setting and population:**   - Patient records identified from the MBSAQIP PUF from 2015-2017 | - Age - Sex - Race | 1. Postoperative Sepsis (PS), stratified by presence or absence of organ/space surgical site infection (OS-SSI) | **Statistical analysis:**   - Descriptive statistics between cases and controls - Binary logistic regression used to evaluate independent factors associated with PS (cases) in each subgroup (with and without OS-SSI) adjusted for covariates - “Variables with P <0.1 in univariate analysis were retained in multivariate analysis”   **Results/effect estimates:**  1. With OS-SSI (%)  Age ≥50y   - Cases PS: 47.3% (141/298) - Controls Non-PS: 39.2% (233/594) - OR = 1.44 (95% CI: 1.07-1.93, p = 0.016)   Male   - Cases PS: 21.1% (63/298) - Controls Non-PS: 23.2% (138/594) - P >0.05   White Race   - Cases PS: 81.7% (232/298) - Controls Non-PS: 85.8% (478/594) - P >0.05   Black Race   - Cases PS: 17.6% (50/298) - Controls Non-PS: 12.6% (70/594) - P >0.05   Other Race   - Cases PS: 0.7% (2/298) - Controls Non-PS: 1.6% (9/594) - P >0.05   2. Without OS-SSI  Age ≥50y   - Cases PS: 49.0% (178/363) - Controls Non-PS: 33.9% (148,362/437,497) - OR = 1.30 (95% CI: 1.04-1.63, p = 0.024)   Male   - Cases PS: 29.8% (108/363) - Controls Non-PS: 20.6% (89,997/437,497) - P <0.05   White Race   - Cases PS: 79.4% (269/363) - Controls Non-PS: 79.7% (320,263/437,497) - P >0.05   Black Race   - Cases PS: 18.3% (62/363) - Controls Non-PS: 19.1% (76,679/437,497) - P >0.05   Other Race   - Cases PS: 2.4% (8/363) - Controls Non-PS: 1.3% (5,091/437,497) - P >0.05 | **Author’s conclusion:**  “Development of OS-SSI after primary bariatric surgery is associated with sepsis and increased 30-day mortality. Patients without OS-SSI who develop PS have a significantly higher mortality rate compared with patients with OS-SSI who develop PS. Early identification and intervention in patients with PS, including those without OS-SSI, may improve survival in this high-risk group.”  **Reviewer’s comments:**  This study shows that older age, but not male sex or race, is significantly associated with increased risk of postoperative sepsis with OS-SSI, while older age, and male sex, but not race, is significantly associated with increased rates of postoperative sepsis without OS-SSI.  This study has clearly met 9/10 (90%) criteria in the critical appraisal tool. |
| **Authors:**  Di Palma, et al. [95]  **Year published:** 2021  **Study design:**  Cohort study  **Country:**  Canada | **Sample size:**  N=2,830  **Inclusion criteria:**   - All patients who underwent RYGB at University Health Network, a tertiary care centre in Toronto, Ontario, between 2011 and 2017   **Exclusion criteria:**   - Nil   **Setting and population:**   - Patients from the University Health Network - Patient records identified from the prospectively maintained institutional bariatric surgery database, the MBSAQIP database, the Ontario Bariatric Registry, and individual patient electronic health records | - Age - Sex | 1. Marginal ulceration (including recalcitrant ulcers requiring surgical revision) | **Statistical analysis:**   - Descriptive for means and proportions between cases and controls - Multivariate analysis was performed using a binomial logical regression model adjusted for covariates - “A Cox proportional hazards model was applied to determine adjusted risk factors for patients with recalcitrant ulcers requiring surgical revision in comparison to the medically responsive patients.”   **Results/effect estimates:**  1. Marginal ulceration  Age   - Cases marginal ulceration: mean 43.2y - Controls no marginal ulceration: mean 45.0y - OR = 0.95 (95% CI: 0.93-0.98, p <0.001)   Sex   - Cases marginal ulceration: female 81.0% (158/195), male 19.0% (37/195) - Controls no marginal ulceration: female 83.1% (978/1,177), male 16.9% (199/1,177) - Male sex: OR = 1.18 (95% CI: 0.69-2.02, p = 0.556) | **Author’s conclusion:**  “Patients with a history of smoking and use of immunosuppressive medication were at significantly higher risk of developing MU that failed medical therapy. Additional evidence is needed to inform perioperative management of bariatric patients.”  **Reviewer’s comments:**  This study shows that younger age, but not male sex, is significantly associated with increased risk of marginal ulceration.  This study has clearly met 9/10 (90%) criteria in the critical appraisal tool. |
| **Authors:**  Bal, et al. [96]  **Year published:** 2021  **Study design:**  Cohort study  **Country:**  United States | **Sample size:**  N=31,498  **Inclusion criteria:**   - Patients who underwent either primary LRYGB or LSG between January 2015 and December 2017 at hospitals participating in the American College of Surgeons National Surgical Quality Improvement Program (ACS-NSQIP)   **Exclusion criteria:**   - Nil   **Setting and population:**   - Patient records identified from the American College of Surgeons National Surgical Quality Improvement Program (ACS-NSQIP) PUF database | - Sex | - 30-day postoperative complications separately (superficial SSI, deep incisional SSI, OS-SSI, wound dehiscence, pneumonia, unplanned intubation, PE, prolonged ventilation, acute renal failure (ARF), UTI, cerebrovascular accident (CVA), cardiac arrest, myocardial infarction (MI), blood loss requiring transfusion, DVT, sepsis, septic shock, reoperation), for LSG and LRYGB separately - 30-day mortality, for LSG and LRYGB separately | **Statistical analysis:**   - Descriptive statistics - “Propensity score matching was used to evaluate 30-day hospital readmission rates and postoperative complications as independent variables with other clinically relevant pre-operative characteristics”   **Results/effect estimates:^a^**  1. LSG  Superficial SSI (%)   - Male: 0.51% (55/10,788) - Female: 0.49% (53/10,814) - P = 0.83   UTI (%)   - Male: 0.24% (26/10,788) - Female: 0.55% (59/10,814) - P = 0.0003   Blood loss requiring transfusion (%)   - Male: 0.73% (79/10,788) - Female: 0.60% (65/10,814) - P = 0.24   DVT (%)   - Male: 0.38% (41/10,788) - Female: 0.30% (32/10,814) - P = 0.29   Reoperation (%)   - Male: 1.03% (111/10,788) - Female: 0.97 % (105/10,814) - P = 0.66   30-day mortality (%)   - Male: 0.15% (16/10,788) - Female: 0.05% (5/10,814) - P = 0.02   2. LRYGB  Superficial SSI (%)   - Male: 1.13% (56/4,961) - Female: 1.62% (80/4,935) - P = 0.04   Organ space infection (%)   - Male: 0.79% (39/4,961) - Female: 0.91% (45/4,935) - P = 0.50   UTI   - Male: 0.30% (15/4,961) - Female: 1.01% (50/4,935) - P <0.001   Blood loss requiring transfusion (%)   - Male: 1.43% (71/4,961) - Female: 1.30% (64/4,935) - P = 0.56   Reoperation (%)   - Male: 2.56% (127/4,961) - Female: 2.39% (118/4,935) - P = 0.59   30-day mortality (%)   - Male: 0.24% (12/4,961) - Female: 0.10% (5/4,935) - P = 0.09 | **Author’s conclusion:**  This study found that men “experience more severe postoperative complications following bariatric surgery, despite favouring LSG, which has been associated with lesser operative risks. … These findings also indicate the need to examine healthcare barriers faced by male patients in the context of follow-up care, as well as, guide discussions about the need for close postoperative monitoring given the higher number of severe complications in this demographic.”  **Reviewer’s comments:**  This study shows that female sex is significantly associated with increased rates of UTI, but not superficial SSI, blood loss requiring transfusion, DVT, or reoperation, while male sex is significantly associated with increased rates of mortality following LSG. It also shows that female sex is significantly associated with increased rates of superficial SSI, and UTI, but not organ space infection, blood loss requiring transfusion, or reoperation, following LRYGB. Although not statistically significant, mortality rates tended to be higher among female LRYGB patients.  This study has clearly met 5/9 (56%) criteria in the critical appraisal tool. |
| **Authors:**  Athanasiadis, et al. [97]  **Year published:** 2021  **Study design:**  Cohort study  **Country:**  United States | **Sample size:**  N=1,045  **Inclusion criteria:**   - All septuagenarian (≥70y) patients who underwent primary LRYGB and LSG procedures during 2011-2015   **Exclusion criteria:**   - Nil   **Setting and population:**   - Patient records identified from the prospectively maintained departmental MBSAQIP database | - Age | 1. Postoperative complications group (30-day, up to 4-year for any reintervention or reoperation) | **Statistical analysis:**   - Descriptive statistics - Multivariable binary regression used to compare complication rates by age group, adjusted for covariates   **Results/effect estimates:**  1. Postoperative complications (%)  30-day complications (%)   - Age ≥70y (n=29): 10% - Age 60-70y (n=90): 8% - Age <60y (n=850): 6% - P = 0.530   Overall, 4-year complications (%)   - Age ≥70y (n=29): 38% - Age 60-70y (n=80): 28% - Age <60y (n=739): 23% - P = 0.014 | **Author’s conclusion:**  “In our study, we identified that bariatric surgery can be accomplished on septuagenarian patients with appropriate weight loss, but long-term complications are a concern. When possible, bariatric surgery should be performed earlier in life as it provides a greater impact on comorbidity resolution and is safer. Nevertheless, with the presence of obesity in septuagenarians rising, bariatric surgeons should consider broadening their patient selection criteria to include and select ≥ 70-yearold patients who are appropriate candidates for surgery.”  **Reviewer’s comments:**  This study shows that older age is significantly associated with increased risk of overall postoperative complications within 4 years, but not within 30 days.  This study has clearly met 7/9 (78%) criteria in the critical appraisal tool. |
| **Authors:**  Aryannezhad, et al. [98]  **Year published:** 2021  **Study design:**  Cohort study  **Country:**  Iran | **Sample size:**  N=168  **Inclusion criteria:**   - Patients who underwent sleeve gastrectomy (SG) or gastric bypass (GB), from March 2013 to March 2017 in the Tehran Obesity Treatment Centre - Controls were matched based on sex, BMI at baseline, and surgery type   **Exclusion criteria:**   - Nil   **Setting and population:**   - Patients from the Tehran Obesity Treatment Centre | - Age | 1. Postoperative complications separately (haemorrhage, MI, PE, infectious, DVT, reintubation, prolonged hospitalisation (>7 days)) 2. Mortality | **Statistical analysis:**   - Chi-square test for proportions of postoperative complications between older (aged ≥60y) and younger (aged 18-60y) groups   **Results/effect estimates:**  1. Postoperative complications (%)  Haemorrhage   - Age ≥60y: 3.1% (2/56) - Age 18-60y: 0.9% (1/112) - P = 0.59   PE   - Age ≥60y: 0% (0/56) - Age 18-60y: 0.9% (1/112)   Infectious   - Age ≥60y: 1.7% (1/56) - Age 18-60y: 0% (0/112)   Prolonged hospitalisation   - Age ≥60y: 7.1% (4/56) - Age 18-60y: 4.5% (5/112) - P = 0.44   DVT   - Age ≥60y: 0% (0/56) - Age 18-60y: 0% (0/112)   2. Mortality   - Age ≥60y: 0% (0/56) - Age 18-60y: 0% (0/112) | **Author’s conclusion:**  “Bariatric surgery is a safe intervention for the management of obesity and obesity-related co-morbidities in older adults, with similar surgery-risk and complication rates to those of younger adults.”  **Reviewer’s comments:**  This study shows that older age is not associated with increased rates of postoperative complications, or mortality.  This study has clearly met 8/9 (89%) criteria in the critical appraisal tool. |
| **Authors:**  Welsh, et al. [99]  **Year published:** 2020  **Study design:**  Cohort study  **Country:**  United States | **Sample size:**  N=212,970  **Inclusion criteria:**   - Adult patients who underwent a laparoscopic primary bariatric operation of RYGB, SG, or biliopancreatic diversion with duodenal switch (BPD-DS in 2015 and 2016   **Exclusion criteria:**   - Patients who had a revision/conversion principal operative procedure - Patients who were under the age of 18 - Patients who were underweight (with a BMI of <18.5) post-operation - Patients who were of an unknown race - Patients who did not have complete data for the relevant covariates of interest   **Setting and population:**   - Patient records identified from the 2015 and 2016 MBSAQIP PUF | - Race - Age - Sex | 1. 30-day readmission | **Statistical analysis:**   - Descriptive statistics - “A multivariable, logistic regression analysis was conducted to model the relationship between race and 30-day readmission, after adjusting for relevant variables as covariates.”   **Results/effect estimates:^b^**  1. 30-day readmission  Race   - White (Ref): 4.2% (6,155/145,007) - Black: 6.0% (2,223/36,970), OR = 1.39 (95% CI: 1.32-1.46, p <0.0001) - Asian: 3.9% (38/985), OR = 0.95 (95% CI: 0.68-1.31, p = 0.74) - Hispanic: 4.2% (1,198/28,673), OR = 1.02 (95% CI: 0.96-1.09, p = 0.50) - Other: 4.0% (53/1,335), OR = 0.93 (95% CI: 0.70-1.22, p = 0.59)   Age   - OR = 0.99 (95% CI: 0.99-0.99, p <0.0001)   Sex   - Male vs Female: OR = 0.85 (95% CI: 0.81-0.90, p <0.0001) | **Author’s conclusion:**  “Black patients have higher odds of readmission and multiple grades of complications (including death) compared with White patients. Hispanic patients have higher odds of a Grade 3 complication compared with White patients. No significant differences were found with other races.”  **Reviewer’s comments:**  This study shows that black race, younger age, and female sex is significantly associated with increased risk of 30-day readmission.  This study has clearly met 7/9 (78%) criteria in the critical appraisal tool. |
| **Authors:**  Turchi, et al. [100]  **Year published:** 2020  **Study design:**  Cohort study  **Country:**  Argentina | **Sample size:**  N=582  **Inclusion criteria:**   - Patients who underwent LRYGB between March 2008 and December 2018 in the Hospital Privado de Comunidad in Mar del Plata, Argentina   **Exclusion criteria:**   - Nil   **Setting and population:**   - Patients from the Hospital Privado de Comunidad in Mar del Plata, Argentina | - Age | 1. Postoperative complications group and separately (within 30 days after surgery) (upper gastrointestinal bleeding, intrabdominal abscess, dehydration, PE, anastomosis stenosis, intestinal obstruction, symptomatic gallstones, leak, trocar point hernia) | **Statistical analysis:**   - Descriptive statistics - “Multivariable binary logistic regression analyses were performed to evaluate whether age was independently associated with the occurrence of postoperative complications”   **Results/effect estimates:**  1. Postoperative complications (%)  Overall   - Young (18-39y): 9.6% (17/177) - Middle-aged (40-59y): 6.8% (24/352) - Elderly (≥60y): 7.5% (4/53)   Upper gastrointestinal bleeding   - Young: 4.0% (7/177) - Middle-aged: 3.7% (13/352) - Elderly: 3.8% (2/53)   Anastomosis stenosis   - Young: 1.1% (2/177) - Middle-aged: 0.9% (3/352) - Elderly: 0% (0/53)   Intestinal obstruction   - Young: 1.7% (3/177) - Middle-aged: 0.6% (2/352) - Elderly: 1.9% (1/53)   Leak   - Young: 0% (0/177) - Middle-aged: 1.1% (4/352) - Elderly: 1.9% (1/53) | **Author’s conclusion:**  “In this study, age was not found to be an independent predictor of postoperative complications nor the likelihood of co-morbidity resolution. Therefore, older age alone should not be an absolute contraindication for LRYGB.”  **Reviewer’s comments:**  This study shows that older age is not associated with increased rates of postoperative complications.  This study has clearly met 8/9 (89%) criteria in the critical appraisal tool. |
| **Authors:**  Sundaresan, et al. [101]  **Year published:** 2020  **Study design:**  Cohort study  **Country:**  United States | **Sample size:**  N=106,706  **Inclusion criteria:**   - Patients who underwent RYGB or SG between 2015 and 2017   **Exclusion criteria:**   - Patients who were <18y, underwent revisions/conversions, miniloop GB, gastric plication, endoscopic therapies, emergency cases, hand-assisted cases, open procedures, or intragastric balloon procedures - Patients with unknown Hispanic ethnic status (separate to racial status)   **Setting and population:**   - Patient records identified from the MBSAQIP PUF gathered from 832 academic and community MBSAQIP-accredited centres between 2015 and 2017 - “Matching was performed on patient demographic characteristics (age, BMI, and race) and co-morbid risk factors” | - Race | - 30-day postoperative complications group (minor, major) and separately (minor: wound disruption, SSI, UTI; major: deep SSI, organ space SSI, ARF, cardiac arrest requiring resuscitation, coma >24 hours, CVA, MI, ventilator use >48 hours, pneumonia, progressive renal insufficiency, venous thromboembolism (VTE), PE, sepsis, septic shock, unplanned intubation, and unplanned admission to intensive care unit (ICU); dehydration, VTE, bleeding, leak), for SG and RYGB separately - 30-day mortality for SG and RYGB separately | **Statistical analysis:**   - Descriptive statistics - Chi-square analysis used to compare complication rates between Hispanic and non-Hispanic patients for all procedures and separately for RYGB and SG   **Results/effect estimates:^a^**  1. RYGB  Major complication (%)   - Hispanic (n=14,447): 1.3% - Non-Hispanic (n=14,447): 1.6% - P <0.05   Minor complication (%)   - Hispanic: 1.5% - Non-Hispanic: 1.8% - P <0.05   Superficial SSI   - Hispanic: 0.7% - Non-Hispanic: 0.9% - P = 0.0782   UTI   - Hispanic: 0.5% - Non-Hispanic: 0.6% - P = 0.0887   30-day ICU admission   - Hispanic: 0.8% - Non-Hispanic: 0.9% - P = 0.3738   30-day mortality   - Hispanic: 0.1% - Non-Hispanic: 0.1% - P = 0.2391   2. SG  Major complication (%)   - Hispanic (n=38,906): 0.7% - Non-Hispanic (n=38,906): 0.8% - P = 0.2802   Minor complication (%)   - Hispanic: 0.6% - Non-Hispanic: 0.7% - P = 0.1998   UTI (%)   - Hispanic: 0.3% - Non-Hispanic: 0.3% - P = 0.4996   30-day ICU admission (%)   - Hispanic: 0.3% - Non-Hispanic: 0.4% - P <0.05   Dehydration (%)   - Hispanic: 0.5% - Non-Hispanic: 0.4% - P = 0.0934   30-day mortality   - Hispanic: 0.0% - Non-Hispanic: 0.1% - P = 0.1047 | **Author’s conclusion:**  “Hispanic patients undergo disproportionately low rates of metabolic and bariatric surgery procedures and present with lower incidence of preoperative co-morbidities. Additionally, Hispanic patients have the same or decreased incidence of postoperative complications compared with non-Hispanic patients, thereby corroborating the Hispanic paradox.”  **Reviewer’s comments:**  This study shows that non-Hispanic race is significantly associated with increased rates of major and minor postoperative complications, but not superficial SSI, UTI, ICU admission, or mortality, following RYGB. It also shows that non-Hispanic race is significantly associated with increased rates of ICU admission, but not major or minor complications, UTI, dehydration, or mortality, following SG.  This study has clearly met 7/9 (78%) criteria in the critical appraisal tool. |
| **Authors:**  Molero, et al. [102]  **Year published:** 2020  **Study design:**  Cohort study  **Country:**  Spain | **Sample size:**  N=261  **Inclusion criteria:**   - Patients aged >50y who had undergone LSG as a stand-alone procedure in the university hospital from January 2007 to December 2013, with a follow-up of 1-5 years   **Exclusion criteria:**   - Patients with liver cirrhosis - Patients with chronic renal failure (creatinine ≥2 mg/dl or glomerular filtration less than 40 ml/min) - Patients with a follow-up less than 1 year - Patients with no information about the primary variable of the study   **Setting and population:**   - Patient records identified and reviewed from a prospectively maintained database in the institution (university hospital) - Younger patients were matched with the older patients for sex, BMI, excess body weight, smoking, and comorbidities | - Age | 1. 30-day postoperative complications group | **Statistical analysis:**   - Descriptive statistics between older (age ≥60y) and younger (age 50-59y)   **Results/effect estimates:**  1. Postoperative complications (%)   - Age ≥60y: 8% (9/116) - Age 50-59y: 11% (16/145) | **Author’s conclusion:**  “LSG provides acceptable outcomes and is safe in older adults indicating that age should not be a limitation to perform BS in this population.”  **Reviewer’s comments:**  This study shows that older age is not associated with increased rates of postoperative complications.  This study has clearly met 8/9 (89%) criteria in the critical appraisal tool. |
| **Authors:**  Mocanu, et al. [103]  **Year published:** 2020  **Study design:**  Cohort study  **Country:**  Canada, United States | **Sample size:**  N=430,936  **Inclusion criteria:**   - Patients who underwent primary LSG or RYGB from 2015 to 2017   **Exclusion criteria:**   - Patients with prior bariatric surgery - Patients who underwent emergency surgery   **Setting and population:**   - Patient records identified from the MBSAQIP PUF gathered from over 832 accredited centres across the United States and Canada between 2015 and 2017 | - Race - Sex - Age | 1. 30-day major postoperative complications group (leak, bleed, reintervention, reoperation, unplanned intubation, length of stay >7 days, ARF, cardiac arrest, coma >24 hr, CVA, MI, VTE) 2. Mortality | **Statistical analysis:**   - “Non-parsimonious multivariable logistic regression models were developed to determine predictive factors for mortality and major complications.” Effects expressed as OR, adjusted for covariates.   **Results/effect estimates:**  1. Major postoperative complications   - Older age (per 1 year): OR = 1.05 (95% CI: 1.04-1.06, p = 0.30) - Female: OR = 1.05 (95% CI: 1.01-1.10, p = 0.03) - Race: Black vs White: OR = 1.30 (95% CI: 1.24-1.35, p <0.001) - Race: Other vs White: OR = 0.90 (95% CI: 0.84-0.96, p = 0.001)   2. Mortality   - Older age (per 1 year): OR = 1.05 (95% CI: 1.04-1.06, p <0.001) - Female: OR = 0.53 (95% CI: 0.42-0.65, p <0.001) - Race: Black: OR = 1.78 (95% CI: 1.39-2.26, p <0.001) - Race: Other: OR = 0.89 (95% CI: 0.58-1.35, p = 0.581) | **Author’s conclusion:**  “Race and sex are independent predictors of adverse outcomes following bariatric surgery in a multivariate logistic regression analysis of the MBSAQIP database. The influence of these factors requires further study in order to continue optimizing bariatric surgery outcomes.”  **Reviewer’s comments:**  This study shows that female sex, and black race, but not older age, are significantly associated with increased risk of postoperative complications, while other non-White races are significantly associated with decreased risk. It also shows that older age, male sex, and black race, but not other non-White races, are significantly associated with increased risk of mortality.  This study has clearly met 7/9 (78%) criteria in the critical appraisal tool. |
| **Authors:**  Maloney, et al. [104]  **Year published:** 2020  **Study design:**  Cohort study  **Country:**  United States | **Sample size:**  N=279,419  **Inclusion criteria:**   - Patients who underwent LRYGB or LSG from 2015 to 2016   **Exclusion criteria:**   - Patients with revision/conversion flag, mini-loop GB, gastric-plication, endoscopic therapy, “other” flag, emergency cases, hand-assisted cases, N.O.T.E.S flag, open flag, and intragastric balloon flag   **Setting and population:**   - Patient records identified from the MBSAQIP PUF gathered from 791 accredited centres from the beginning of January 2015 to the end of December 2016 | - Age - Sex - Race | - Postoperative complications group (major: ARF, cardiac arrest requiring cardiopulmonary resuscitation, coma >24 hr, CVA, MI, ventilator use >48 hr, pneumonia, progressive renal insufficiency, PE, sepsis, septic shock, unplanned intubation, unplanned admission to ICU; Surgical Site Occurrences (SSO): wound disruption, SSI, deep SSI, organ space SSI) - Reoperation - Readmission - Mortality | **Statistical analysis:**   - “Multivariate stepwise logistic regression analysis was performed, controlling for clinically relevant covariables to generate odds ratios (ORs) for factors associated with adverse outcomes”   **Results/effect estimates:**  1. Major complications   - Age 45-54y vs control (18-44y): OR = 1.45 (95% CI: 1.31-1.61, p <0.05) - Age 55-64y vs control: OR = 1.96 (95% CI: 1.76-2.18, p <0.05) - Age 65y+ vs control: OR = 2.80 (95% CI: 2.45-3.21, p <0.05) - Female: OR = 0.67 (95% CI: 0.62-0.73, p <0.05) - Black vs White: OR = 1.33 (95% CI: 1.20-1.46, p <0.05) - Hispanic vs White: OR = 0.81 (95% CI: 0.69-0.95, p <0.05)   2. SSO complication   - Age 45-54y vs control: p >0.05 - Age 55-64y vs control: OR = 1.55 (95% CI: 1.18-2.02, p <0.05) - Age 65y+ vs control: OR = 1.54 (95% CI: 1.04-2.28, p <0.05) - Female: p >0.05 - Black vs White: p >0.05 - Hispanic vs White: p >0.05   3. Reoperation   - Age 45-54y vs control: p >0.05 - Age 55-64y vs control: p >0.05 - Age 65y+ vs control: p >0.05 - Female: p >0.05 - Black vs White: OR = 1.10 (95% CI: 1.00-1.20, p <0.05) - Hispanic vs White: p >0.05   4. Readmission   - Age 45-54y vs control: OR = 0.89 (95% CI: 0.85-0.93, p <0.05) - Age 55-64y vs control: OR = 0.90 (95% CI: 0.85-0.96, p <0.05) - Age 65y+ vs control: p >0.05 - Female: OR = 1.18 (95% CI: 1.12-1.24, p <0.05) - Black vs White: OR = 1.39 (95% CI: 1.33-1.46, p <0.05) - Hispanic vs White: p >0.05   5. Mortality   - 45-54y vs control: OR = 1.93 (95% CI: 1.38-2.70, p <0.05) - 55-64y vs control: OR = 3.18 (95% CI: 2.26-4.47, p <0.05) - 65y+ vs control: OR = 5.35 (95% CI: 3.58-7.98, p <0.05) - Female: OR = 0.48 (95% CI: 0.38-0.61, p <0.05) - Black vs White: OR = 1.73 (95% CI: 1.30-2.30, p <0.05) - Hispanic vs White: p >0.05 | **Author’s conclusion:**  “Overall, bariatric surgery (SG or RYGB) remains a low mortality risk procedure for all age groups. However, all age group classifications > 45 years had higher incidence of major complications and mortality compared to patients 18–45 years (despite older individuals having lower preoperative BMI) indicating delaying surgery is detrimental.”  **Reviewer’s comments:**  This study shows that older age is significantly associated with increased risk of major complications, SSO complications, and mortality, and decreased risk of readmission, but not reoperation. Female sex is significantly associated with decreased risk of major complications, and mortality, significantly increased risk of readmission, and no significant difference in risk of SSO complications, or reoperation. Black race is significantly associated with increased risk of major complications, reoperation, readmission, and mortality, but not SSO complications. Hispanic race is significantly associated with decreased risk of major complications, but not SSO complications, reoperation, readmission, or mortality.  This study has clearly met 7/9 (78%) criteria in the critical appraisal tool. |
| **Authors:**  Hoffman, et al. [105]  **Year published:** 2020  **Study design:**  Cohort study  **Country:**  United States | **Sample size:**  N=30,266  **Inclusion criteria:**   - Male sex - Patients who underwent metabolic and bariatric surgery (LRYGB, laparoscopic adjustable gastric banding (LAGB), LSG) between January 1, 2015 and December 31, 2015   **Exclusion criteria:**   - Patients whose age was not specified - Patients with age <20y - Patients with unspecified preoperative BMI - Patients belonging to other races, who were not White or African American - Patients who did not report their race or reported as “unknown”   **Setting and population:**   - Patient records identified from the 2015 MBSAQIP PUF | - Race - Age | 1. 30-day postoperative complications group (SSI, deep incisional SSI, organ/space SSI, wound disruption, pneumonia, unplanned intubation, PE, ventilator >48 hr, progressive renal insufficiency, ARF, UTI, stroke/CVA, coma >24 hr, peripheral nerve injury, cardiac arrest, MI, transfusion within 72 hr, vein thrombosis requiring therapy, sepsis, septic shock) | **Statistical analysis:**   - Descriptive statistics - Unconditional multivariate logistic regression models used to examine the association between patient characteristics and postoperative complications among men only, adjusted for covariates   **Results/effect estimates:**  1. Postoperative complications (%)  Race   - African-American: 3.4% (125/3,643) - White (Ref): 2.8% (753/26,623) - OR = 1.25 (95% CI: 1.02-1.52, p <0.05)   Age (years):   - OR = 1.01 (95% CI: 1.00-1.02, p <0.05) | **Author’s conclusion:**  “Despite being eligible for MBS based on both BMI and obesity-related comorbidities, AA men are significantly less likely to undergo MBS. Those AA men who receive surgery are significantly younger than White men but also experience greater comorbidities compared to White men and all women. Further longitudinal studies into patient-, system-, and provider-level barriers are necessary to understand and address these disparities.”  **Reviewer’s comments:**  This study shows that African-American race, and older age are significantly associated with increased risk of postoperative complications.  This study has clearly met 7/9 (78%) criteria in the critical appraisal tool. |
| **Authors:**  Haal, et al. [106]  **Year published:** 2020  **Study design:**  Case-control study  **Country:**  Netherlands | **Sample size:**  N=699  **Inclusion criteria:**   - All patients (aged 18-65y) who underwent primary LRYGB between January 2013 and December 2015 - Patients who underwent cholecystectomy because of postoperative biliary symptoms (biliary colic, cholecystitis, choledocholithiasis, cholangitis, biliary pancreatitis) which developed within 2 yrs of LRYGB (cases) - Patients who underwent LRYGB after each corresponding case and who remained free of biliary symptoms and cholecystectomy, and with minimum follow-up period of 24 months (controls)   **Exclusion criteria:**   - Patients with prior or concomitant gallbladder surgery - Patients with prior bariatric surgery - Patients with another type of bariatric surgery than LRYGB - Patients with the development of symptomatic gallstones more than 2 yrs after LRYGB   **Setting and population:**   - Patient records identified from the electronic medical record system at the Medical Centre Slotervaart in Amsterdam, the Netherlands | - Age - Sex - Ethnicity | 1. Cholecystectomy (because of postoperative biliary symptoms which developed within 2 yrs of LRYGB) | **Statistical analysis:**   - “Univariate logistic regression analysis was used to evaluate associations between potential risk factors and the occurrence of cholecystectomy. Additionally, all variables were entered in a multivariable logistic regression analysis (full model). Using stepwise backward elimination, a final model was derived (in each subsequent step, the least significant variable in the model was removed until all remaining variables have individual p values smaller than 0.125)’   **Results/effect estimates:**  1. Cholecystectomy   - Age: OR = 0.98 (95% CI: 0.96-0.99, p = 0.005) - Female: OR = 1.83 (95% CI: 1.06-3.17, p = 0.031) - Caucasian: OR = 1.82 (95% CI: 1.10-3.02, p = 0.019) | **Author’s conclusion:**  “In our study, higher %TWL and preoperative pain syndrome were associated with an increased risk for cholecystectomy besides the traditional risk factors female gender and Caucasian ethnicity. These factors can be used to identify high-risk patients, who might benefit from preventive measures.”  **Reviewer’s comments:**  This study shows that younger age, female sex, and Caucasian ethnicity are significantly associated with increased risk of requiring a subsequent cholecystectomy.  This study has clearly met 9/10 (90%) criteria in the critical appraisal tool. |
| **Authors:**  Gambhir, et al. [107]  **Year published:** 2020  **Study design:**  Cohort study  **Country:**  United States, Canada | **Sample size:**  N=369,032  **Inclusion criteria:**   - Patients who underwent LSG and LRYGB between January 1, 2015 and December 31, 2017   **Exclusion criteria:**   - Patients under the age of 18 - Patients with an ASA physical status class equal or greater than 4 - Patients undergoing emergent, revisional, or laparoscopic-converted-to-open cases   **Setting and population:**   - Patient records identified from the MBSAQIP database gathered from 814 accredited centres across the United States and Canada from January 1, 2015 to December 31, 2017 | - Age - Sex - Race | 1. DVT 2. PE | **Statistical analysis:**   - Descriptive statistics - “Multivariate logistic regression analysis was used to determine adjusted odds ratios (OR) for the risk of DVT and PE”, controlling for clinically relevant variables   **Results/effect estimates:**  1. DVT   - Age: OR = 1.0 (95% CI: 0.99-1.01, p = 0.58) - Sex (male vs female): OR = 1.3 (95% CI: 1.06-1.57, p = 0.012) - Race (black vs white): OR = 1.3 (95% CI: 1.03-1.56, p = 0.02)   2. PE   - Age: OR = 1.0 (95% CI: 1.00-1.03, p = 0.007) - Sex (male vs female): OR = 0.9 (95% CI: 0.69-1.20, p = 0.51) - Race (black vs white): OR = 1.9 (95% CI: 1.48-2.42, p <0.001) | **Author’s conclusion:**  “Prolonged operative length is associated with a higher risk of DVT and PE after either LSG or LRYGB. Transfusion and history of DVT are the largest risk factors for developing DVT and PE. There is a decreased risk of PE after LSG compared to LRYGB.”  **Reviewer’s comments:**  This study shows that male sex, and black race, but not older age, are significantly associated with increased risk of DVT. It also shows that black race, but not older age or male sex, is significantly associated with increased risk of PE.  This study has clearly met 7/10 (70%) criteria in the critical appraisal tool. |
| **Authors:**  Dugan, et al. [108]  **Year published:** 2020  **Study design:**  Cohort study  **Country:**  United States | **Sample size:**  N=429,664  **Inclusion criteria:**   - Patients who underwent primary SG and RYGB between 2015 and 2016   **Exclusion criteria:**   - Patients undergoing emergent, endo-therapy, gastric plication, revisional and balloon procedures - Patients with incomplete data   **Setting and population:**   - Patient records identified from the MBSAQIP PUF gathered from more than 800 accredited centres between 2015 and 2016 | - Sex | - 30-day postoperative complications group and separately (major: ARF, cardiac arrest, coma >24hr, CVA, MI, postoperative ventilator, progressive renal insufficiency, PE, sepsis, septic shock, unplanned intubation, VTE, OS-SSI, unplanned ICU admission; minor: postoperative SSI, UTI, wound disruption, incisional SSI; bleeding, dehydration, leak), for SG and RYGB separately - 30-day mortality, for SG and RYBG separately | **Statistical analysis:**   - Descriptive statistics - PSM used to compare outcomes between male (n=88,426) and female (n=341,238) groups   **Results/effect estimates:^a^**  1. LSG  Major complication (%)   - Male: 1.12% - Female: 0.89% - P <0.0001   Minor complication (%)   - Male: 0.43% - Female: 0.75% - P <0.0001   Superficial SSI (%)   - Male: 0.19% - Female: 0.25% - P = 0.4944   UTI (%)   - Male: 0.12% - Female: 0.34% - P <0.0001   Unplanned ICU admission (%)   - Male: 0.61% - Female: 0.48% - P <0.0001   30-day mortality (%)   - Male: 0.1% - Female: 0.05% - P <0.0001   2. RYGB  Major complication (%)   - Male: 2.21% - Female: 1.7% - P <0.0001   Minor complication (%)   - Male: 1.33% - Female: 1.97% - P <0.0001   Superficial SSI (%)   - Male: 0.71% - Female: 0.95% - P <0.0001   UTI (%)   - Male: 0.21% - Female: 0.59% - P <0.0001   Unplanned ICU admission (%)   - Male: 1.46% - Female: 1.02% - P <0.0001   30-day mortality (%)   - Male: 0.23% - Female: 0.12% - P <0.0001 | **Author’s conclusion:**  “Male gender is an independent risk factor for major post-operative complications and 30-day mortality, even after controlling for comorbidities.”  **Reviewer’s comments:**  This study shows that male sex is significantly associated with increased risk of major complications, unplanned ICU admission, and mortality, decreased risk of minor complications, and UTI, but not superficial SSI, following LSG. It also shows that male sex is significantly associated with increased risk of major complications, unplanned ICU admission, and mortality, and decreased risk of minor complications, superficial SSI, and UTI following RYGB.  This study has clearly met 5/9 (56%) criteria in the critical appraisal tool. |
| **Authors:**  Amirian, et al. [109]  **Year published:** 2020  **Study design:**  Cohort study  **Country:**  United States, Canada | **Sample size:**  N=106,932  **Inclusion criteria:**   - Patients aged ≥18 yrs - Patients with BMI ≥35 - Patients who underwent either primary LRYGB or LSG in 2016 - Patients with known information on race   **Exclusion criteria:**   - Patients with unknown race or ethnicity information - Patients with previous bariatric surgery - Patients undergoing revisional or conversional procedures - Patients undergoing emergency surgery - Patients undergoing any surgical approach other than conventional laparoscopic approach   **Setting and population:**   - Patient records identified from the 2016 MBSAQIP PUF gathered from over 790 centres between January 1, 2016 and December 31, 2016 | - Race | 1. 30-day postoperative complications group (superficial incisional SSI, deep incisional SSI, OS-SSI, wound disruption, sepsis, septic shock, UTI, ARF, progressive renal insufficiency, mechanical ventilation >48 hr, unplanned intubation, pneumonia, unplanned admission to ICU, DVT, PE, cardiac arrest, MI, stroke, coma >24 hr, peripheral nerve injury, Clostridium difficile colitis, dehydration requiring treatment, incisional hernia, presence of operative drain at 30 days) | **Statistical analysis:**   - Descriptive statistics - “Multivariate logistic regression models were used to examine the effect of race on outcomes”   **Results/effect estimates:**  1. Postoperative complications   - Black or African American: OR = 1.134 (95% CI: 1.064-1.209, p <0.0001) - Asian: OR = 0.781 (95% CI: 0.519-1.176, p = 0.236) - American Indian or Alaska Native: OR = 1.286 (95% CI: 0.874-1.893, p = 0.201) - Native Hawaiian or other Pacific Islanders: OR = 1.301 (95% CI: 0.861-1.966, p = 0.211) | **Author’s conclusion:**  “This study found significant racial differences in short-term outcomes following bariatric surgery. Factors underlying these disparities are unclear and warrant further investigation.”  **Reviewer’s comments:**  This study shows that black or African-American race, but not Asian, American Indian or Alaskan Native, or Native Hawaiian or other Pacific Islanders, is significantly associated with increased risk of postoperative complications.  This study has clearly met 7/9 (78%) criteria in the critical appraisal tool. |
| **Authors:**  Alimogullari and Bulus[110]  **Year published:** 2020  **Study design:**  Cohort study  **Country:**  Turkey | **Sample size:**  N=111  **Inclusion criteria:**   - Patients who underwent SG between June 2015 and June 2016 - Patients with normal hepatobiliary ultrasound findings in the preoperative period   **Exclusion criteria:**   - Patients with a history of cholecystectomy - Patients who had undergone concurrent cholecystectomy with bariatric surgery - Patients who had undergone ursodeoxycholic acid treatment in the postoperative period - Patients without a regular follow-up   **Setting and population:**   - Not reported | - Age - Sex | 1. Presence of gallstones or bile sludge in gallbladder | **Statistical analysis:**   - Descriptive statistics - “A logistic regression analysis was used to determine the independent samples affecting gallstone formation.”   **Results/effect estimates:**  1. Gallstones or bile sludge  Age (y)   - Cases Gallstones: mean 39.4(±11.3)y - Controls No gallstones: mean 38.5(±10.6)y - P = 0.677   Female (%)   - Cases Gallstones: 78% (32/41) - Controls No gallstones: 82% (58/70) - P = 0.532 | **Author’s conclusion:**  “In our study, we found no association between early and late weight loss rates and gallstone formation. Dyslipidemia was the only pre-operative comorbidity that was independently and significantly associated with gallstone formation. However, more studies are needed in order to recommend prophylactic cholecystectomy for patients with dyslipidemia. SG is a promising surgical approach, and prospective, randomized controlled trials and long-term follow-up studies with a larger series will contribute to the literature.”  **Reviewer’s comments:**  This study shows that older age, and female sex are not associated with increased rates of gallstones or bile sludge in the gallbladder.  This study has clearly met 9/10 (90%) criteria in the critical appraisal tool. |
| **Authors:**  Wood, et al. [111]  **Year published:** 2019  **Study design:**  Cohort study  **Country:**  United States | **Sample size:**  N=14,210  **Inclusion criteria:**   - Black and white patients who underwent a primary bariatric operation (RYGB, SG, or LAGB) between June 2006 and January 2017   **Exclusion criteria:**   - Nil   **Setting and population:**   - Patient records identified from the Michigan Bariatric Surgery Collaborative (MBSC) registry | - Race | 1. 30-day postoperative complications group (including serious complications: abdominal abscess, bowel obstruction, leak, bleeding, respiratory failure, renal failure, wound infection/dehiscence, VTE, MI or cardiac arrest, death) | **Statistical analysis:**   - Descriptive statistics - Use of “propensity score matching to assemble cohorts of black and white patients who had undergone a primary laparoscopic bariatric operation, including gastric bypass (Roux-en-Y), vertical sleeve gastrectomy, or adjustable gastric band, between June 2006 and January 2017”, adjusting for patient characteristics, surgeon and hospital   **Results/effect estimates:**  1. 30-day postoperative complications  Any complication (%)   - Black: 8.8% (628/7,105) - White: 6.8% (481/7,105) - Adjusted Odds Ratio (AOR) = 1.20 (95% CI: 1.03-1.39)   Serious complication (%)   - Black: 2.5% (178/7,105) - White: 1.9% (135/7,105) - AOR = 1.16 (95% CI: 0.89-1.52) | **Author’s conclusion:**  “Black patients undergoing bariatric surgery in Michigan had significantly higher rates of 30-day complications and resource utilization and experienced lower weight loss at 1 year than a matched cohort of white patients. … Racial and cultural differences among patients should be considered when designing strategies to optimize outcomes with bariatric surgery.”  **Reviewer’s comments:**  This study shows that black race is significantly associated with increased risk of any complication, but not serious complications.  This study has clearly met 7/9 (78%) criteria in the critical appraisal tool. |
| **Authors:**  Walker, et al. [112]  **Year published:** 2019  **Study design:**  Cohort study  **Country:**  United States | **Sample size:**  N=1,943  **Inclusion criteria:**   - Adults aged 19 to 78 - Patients who underwent primary LAGB or RYGB between March 2006 and April 2009   **Exclusion criteria:**   - Patients who underwent bariatric procedures which were not either LAGB or RYGB - Patients who did not report having medical insurance, did not indicate the type of insurance, or had an insurance type that could not be classified   **Setting and population:**   - Patients identified from six geographically diverse clinical centres in the United States (New York, North Carolina, North Dakota, Oregon, Pennsylvania, Washington State) | - Insurance type | 1. Postoperative complications (death within 6 months, major adverse event within 30 days: DVT or VTE, reintervention with the use of a percutaneous, endoscopic or operative technique, failure to be discharged from hospitals within 30 days of surgery), for LAGB and RYGB separately | **Statistical analysis:**   - Descriptive statistics   **Results/effect estimates:^c^**  1. Postoperative complications (%)  LAGB   - Age-qualified (AQ) Medicare (age ≥65y): 0.0% (0/54) - Disability-qualified (DQ) Medicare (age <65y): 3.0% (2/67) - Non-Medicare (NM): 1.8% (7/390)   RYGB   - AQ: 4.7% (2/43) - DQ: 6.7% (12/178) - NM: 4.3% (52/1,211) | **Author’s conclusion:**  “Medicare participants experienced substantial BMI loss and diabetes remission, with a frequency of adverse events similar to that of NM participants.”  **Reviewer’s comments:**  This study shows that there is no significant difference in rates of postoperative complications between different insurance types.  This study has clearly met 5/9 (56%) criteria in the critical appraisal tool. |
| **Authors:**  Vidarsson, et al. [113]  **Year published:** 2019  **Study design:**  Cohort study  **Country:**  Sweden | **Sample size:**  N=40,844  **Inclusion criteria:**   - Patients who underwent a primary RYGB between 2007 and 2014 - Patients with an isolated leak at the gastrojejunostomy (GJ)   **Exclusion criteria:**   - Patients with no leak registered in the Scandinavian Obesity Surgery Registry (SOReg) - Patients with leaks at other locations - Patients with missing charts - Patients with erroneous registrations   **Setting and population:**   - Patient records identified from the SOReg between 2007 and 2014 | - Sex - Age | 1. Leak at the GJ | **Statistical analysis:**   - Descriptive statistics - “Logistic regression was done to estimate odds ratios (ORs) for significant risk factors for leak”   **Results/effect estimates:**  1. Leak at the GJ (%)  Sex   - Female (Ref): 0.54% (167/30,915) - Male: 0.96% (95/9,929) - OR = 1.47 (95% CI: 1.13-1.90)   Age (y)   - ≤32 (Ref): 0.42% (41/9,827) - 33-40: 0.49% (48/9,739), OR = 1.11 (95% CI: 0.73-1.69) - 41-48: 0.63% (67/10,601), OR = 1.31 (95% CI: 0.88-1.94) - ≥49: 0.99% (106/10,677), OR = 1.83 (95% CI: 1.25-2.67) | **Author’s conclusion:**  “GJ leaks occurred in 0.6% of patients. Risk factors were male sex, age ≥49 years, diabetes, operative time !90 minutes, and conversion to open surgery.”  **Reviewer’s comments:**  This study shows that male sex, and older age are significantly associated with increased risk of leak at the GJ.  This study has clearly met 7/10 (70%) criteria in the critical appraisal tool. |
| **Authors:**  Sun, et al. [114]  **Year published:** 2019  **Study design:**  Cohort study  **Country:**  China | **Sample size:**  N=122  **Inclusion criteria:**   - Patients who underwent SG for morbid obesity between December 2011 and May 2019   **Exclusion criteria:**   - Nil   **Setting and population:**   - Patients at the bariatric surgery centre in Huashan Hospital | - Age - Sex | 1. Hypoglycaemia 2. Early dumping syndrome | **Statistical analysis:**   - Descriptive statistics - “The correlation between baseline characteristics and total scores of the EHSS and the DSRS were tested by Spearman’s correlation analysis, respectively. For symptoms suggestive of hypoglycemia and dumping syndrome, logistic regression analysis was used to explore potential risk factors”, adjusting for age, sex, BMI, waist circumference, and low-density protein cholesterol at baseline   **Results/effect estimates:**  1. Hypoglycaemia  Age (y)   - Cases hypoglycaemia: mean 30.87(±8.73)y - Controls No hypoglycaemia: mean 36.47(±12.45)y - P = 0.0128 - R = -0.14509, p = 0.1629   Male sex (%)   - Cases hypoglycaemia: 33.33% (27/81) - Controls No hypoglycaemia: 46.34% (19/41) - P = 0.1614 - R = 0.09, p = 0.3863   2. Early dumping syndrome  Age (y)   - Cases Dumping group: mean 30.84(±9.76)y - Controls No dumping group: mean 34.09(±10.74)y - P = 0.1392 - R = -0.21921, p = 0.0338   Male sex (%)   - Cases Dumping group: 32% (16/50) - Controls No dumping group: 41.67% (30/72) - P = 0.2786 - R = -0.04, p = 0.6995 | **Author’s conclusion:**  “Mild-to-moderate symptoms suggestive of hypoglycemia  and dumping were rather common in SG patients, with the prevalence rates of 66.39% (81/122) and 40.98% (50/122), respectively. Younger age and lower LDL-C indicated higher risk for hypoglycemia and early dumping, respectively. LDL-C may mediate in the association between younger age and hypoglycemia.”  **Reviewer’s comments:**  This study shows that younger age is significantly associated with increased risk of hypoglycaemia, although it became not statistically significant after adjustment for LDL-C in a subgroup analysis. Male sex is not associated with increased risk of hypoglycaemia. It also shows that younger age, but not male sex, is significantly associated with increased risk of early dumping syndrome.  This study has clearly met 7/10 (70%) criteria in the critical appraisal tool. |
| **Authors:**  Stenberg, et al. [115]  **Year published:** 2019  **Study design:**  Cohort study  **Country:**  Sweden | **Sample size:**  N=41,537  **Inclusion criteria:**   - Patients who underwent a primary LAGB in Sweden between 2010 and 2016   **Exclusion criteria:**   - Patients aged <18y at the time of surgery   **Setting and population:**   - Patient records identified from the SOReg between 2010 and 2016 | - Education - Profession - Disposable income - Residence - Marital status - Economic aid - Heritage | - 30-day postoperative complications group (any, serious) | **Statistical analysis:**   - Descriptive statistics - Logistic regression was used to evaluate risk of any postoperative complication with OR and 95% CI as measures of association, adjusting for age, BMI, sex and specified comorbidities   **Results/effect estimates:**  1. Any postoperative complication (%)  Education   - Primary education <9y (Ref): 8.4% (n=578) - Secondary education: 8.0% (n=1,924), OR = 0.94 (95% CI: 0.85-1.03, p = 0.190) - Higher education <3y: 8.2% (n=358), OR = 0.95 (95% CI: 0.83-1.10, p = 0.514) - Higher education >3y: 7.9% (n=364), OR = 0.93 (95% CI: 0.81-1.07, p = 0.291)   Profession   - Senior officials and management (Ref): 7.4% (n=92) - Professionals and technicians: 8.0% (n=580), OR = 1.10 (95% CI: 0.87-1.38, p = 0.418) - Clerical support workers: 7.5% (n=231), OR = 1.05 (95% CI: 0.81-1.35, p = 0.727) - Services and sales workers: 7.9% (n=984), OR = 1.14 (95% CI: 0.91-1.43, p = 0.254) - Manual labour: 7.5% (n=368), OR = 1.01 (95% CI: 0.80-1.29, p = 0.913) - Elementary occupation: 8.4% (n=205), OR = 1.18 (95% CI: 0.91-1.53, p = 0.203)   Disposable income (%)   - <20^th^ percentile (Ref): 8.6% (n=919) - 20-50^th^ percentile: 8.3% (n=1,048), OR = 0.92 (95% CI: 0.83-1.01, p = 0.092) - 50-80^th^ percentile: 7.6% (n=911), OR = 0.84 (95% CI: 0.76-0.93, p = 0.001) - >80^th^ percentile: 7.5% (n=334), OR = 0.84 (95% CI: 0.72-0.98, p = 0.030)   Residence (%)   - Large city and municipality (Ref): 8.8% (n=1,120) - Medium-sized town and municipality: 7.9% (n=1,235), OR = 0.90 (95% CI: 0.83-0.98, p = 0.017) - Small town, urban area, rural municipality: 7.4% (n=861), OR = 0.84 (95% CI: 0.76-0.92, p = 0.0002)   Marital status   - Married/partner (Ref): 8.3% (n=1,423) - Divorced/widow/widower: 9.5% (n=598), OR = 1.14 (95% CI: 1.03-1.26, p = 0.014) - Single: 7.3% (n=1,202), OR = 0.90 (95% CI: 0.83-0.99, p = 0.022)   Economic aid   - None (Ref): 7.6% (n=2,456) - Retirement pension: 8.1% (n=40), OR = 0.95 (95% CI: 0.68-1.33, p = 0.769) - Disability pension/early retirement: 11.0% (n=487), OR = 1.37 (95% CI: 1.23-1.53, p <0.0001) - Social assistance: 8.9% (n=256), OR = 1.22 (95% CI: 1.07-1.40, p = 0.004)   Heritage   - Swedish born, Swedish descendant (Ref): 7.7% (n=2,474) - Swedish born, non-Swedish descendant: 9.1% (n=176), OR = 1.22 (95% CI: 1.04-1.44, p = 0.014) - Born outside Sweden: 9.3% (n=572), OR = 1.20 (95% CI: 1.09-1.32, p = 0.0002)   2. Serious postoperative complication (%)  Education   - Primary education <9y (Ref): 3.6% (n=246) - Secondary education: 3.2% (n=763), OR = 0.87 (95% CI: 0.75-1.01, p = 0.063) - Higher education <3y: 3.4% (n=149), OR = 0.93 (95% CI: 0.75-1.14, p = 0.466) - Higher education >3y: 3.3% (n=150), OR = 0.89 (95% CI: 0.72-1.09, p = 0.265)   Profession   - Senior officials and management (Ref): 3.0% (n=37) - Professionals and technicians: 2.9% (n=214), OR = 0.99 (95% CI: 0.70-1.43, p = 0.985) - Clerical support workers: 3.4% (n=106), OR = 1.20 (95% CI: 0.82-1.76, p = 0.351) - Services and sales workers: 3.1% (n=391), OR = 1.12 (95% CI: 0.79-1.59, p = 0.519) - Manual labour: 3.1% (n=150), OR = 1.09 (95% CI: 0.75-1.57, p = 0.635) - Elementary occupation: 3.6% (n=88), OR = 1.28 (95% CI: 0.87-1.90, p = 0.170)   Disposable income (%)   - <20^th^ percentile (Ref): 3.6% (n=389) - 20-50^th^ percentile: 3.4% (n=437), OR = 0.91 (95% CI: 0.79-1.05, p = 0.183) - 50-80^th^ percentile: 3.2% (n=376), OR = 0.79 (95% CI: 0.68-0.92, p = 0.002) - >80^th^ percentile: 2.4% (n=108), OR = 0.57 (95% CI: 0.46-0.72, p <0.0001)   Residence (%)   - Large city and municipality (Ref): 3.2% (n=410) - Medium-sized town and municipality: 3.2% (n=505), OR = 1.02 (95% CI: 0.89-1.17, p = 0.760) - Small town, urban area, rural municipality: 3.3% (n=385), OR = 1.04 (95% CI: 0.91-1.20, p = 0.547)   Marital status   - Married/partner (Ref): 3.2% (n=553) - Divorced/widow/widower: 4.2% (n=268), OR = 1.30 (95% CI: 1.12-1.52, p = 0.001) - Single: 2.9% (n=481), OR = 0.96 (95% CI: 0.84-1.09, p = 0.520)   Economic aid   - None (Ref): 3.1% (n=994) - Retirement pension: 2.8% (n=14), OR = 0.76 (95% CI: 0.44-1.32, p = 0.326) - Disability pension/early retirement: 4.5% (n=199), OR = 1.37 (95% CI: 1.16-1.61, p = 0.0002) - Social assistance: 3.8% (n=109), OR = 1.32 (95% CI: 1.08-1.62, p = 0.008)   Heritage   - Swedish born, Swedish descendant (Ref): 3.2% (n=1,025) - Swedish born, non-Swedish descendant: 3.1% (n=60), OR = 1.00 (95% CI: 0.76-1.30, p = 0.993) - Born outside Sweden: 3.5% (n=217), OR = 1.08 (95% CI: 0.93-1.26, p = 0.288) | **Author’s conclusion:**  “Socioeconomic factors influence the risk for early postoperative complication after laparoscopic gastric bypass surgery. The impact is not enough to exclude patients from surgery, but they must be taken into account in preoperative risk assessment.”  **Reviewer’s comments:**  This study shows that lower disposable income, residence in a large city, being divorced/widow/widower, receiving disability pension or social assistance, and being a first- or second-generation immigrant are significantly associated with increased risk of any postoperative complications. Being single is significantly associated with decreased risk, while education, and profession are not associated. It also shows that lower disposable income, being divorced/widow/widower, and receiving disability pension or social assistance, but not education, profession, residence, or heritage, are significantly associated with increased risk of serious postoperative complications.  This study has clearly met 6/9 (67%) criteria in the critical appraisal tool. |
| **Authors:**  Smith, et al. [116]  **Year published:** 2019  **Study design:**  Cohort study  **Country:**  United States | **Sample size:**  N=62,561  **Inclusion criteria:**   - Patients aged ≥18y who underwent primary RYGB or SG from June 13, 2006 and April 4, 2018   **Exclusion criteria:**   - Patients who underwent revision surgery   **Setting and population:**   - Patient records identified from the MBSC registry | - Age | - 30-day postoperative complications (including severe complications: intrabdominal abscess formation, bowel obstruction/hernia, anastomotic leak, bleeding, respiratory failure, renal failure, wound infection/dehiscence, VTE, MI/cardiac arrest, death), separately for RYGB and SG | **Statistical analysis:**   - Descriptive statistics - A multivariable logistic regression model was used to evaluate the association between age cohorts and 30-day outcomes, controlling for clinically relevant covariates.   **Results/effect estimates:^a^**  1. RYGB  Any complication (%)   - Age 45-69y (Ref) (n=13,267): 10.7% - Age <45y (n=12,014): 11.2%, OR = 1.06 (95% CI: 0.97-1.16) - Age ≥70y (n=202): 14.6%, OR = 1.46 (95% CI: 1.01-2.11)   Severe complication (%)   - Age 45-69y (Ref): 3.4% - Age <45y: 3.0%, OR = 0.88 (95% CI: 0.75-1.03) - Age ≥70y: 4.6%, OR = 1.42 (95% CI: 0.78-2.57)   Haemorrhage (%)   - Age 45-69y (Ref): 3.1% - Age <45y: 2.6%, OR = 0.81 (95% CI: 0.69-0.96) - Age ≥70y: 5.1%, OR = 1.77 (95% CI: 1.01-3.08)   Infection (%)   - Age 45-69y (Ref): 3.3% - Age <45y: 3.4%, OR = 1.05 (95% CI: 0.90-1.22) - Age ≥70y: 2.5%, OR = 0.76 (95% CI: 0.34-1.74)   Obstruction (%)   - Age 45-69y (Ref): 2.0% - Age <45y: 3.0%, OR = 1.35 (95% CI: 1.12-1.61) - Age ≥70y: 1.9%, OR = 0.88 (95% CI: 0.32-2.39)   2. SG  Any complication (%)   - Age 45-69y (Ref) (n=18,410): 5.2% - Age <45y (n=18,229): 5.5%, OR = 1.09 (95% CI: 0.98-1.20) - Age ≥70y (n=439): 7.4%, OR = 1.47 (95% CI: 1.06-2.04)   Serious complication (%)   - Age 45-69y (Ref): 1.5% - Age <45y: 1.6%, OR = 1.11 (95% CI: 0.93-1.33) - Age ≥70y: 2.7%, OR = 1.83 (95% CI: 1.09-3.07)   Leak or perforation (%)   - Age 45-69y (Ref): 0.3% - Age <45y: 0.4%, OR = 1.26 (95% CI: 0.85-1.86) - Age ≥70y: 0.9%, OR = 2.86 (95% CI: 1.01-8.07)   Haemorrhage (%)   - Age 45-69y (Ref): 1.3% - Age <45y: 0.9%, OR = 0.65 (95% CI: 0.52-0.82) - Age ≥70y: 1.9%, OR = 1.53 (95% CI: 0.88-2.67)   Infection (%)   - Age 45-69y (Ref): 1.0% - Age <45y: 1.2%, OR = 1.15 (95% CI: 0.93-1.43) - Age ≥70y: 1.8%, OR = 1.81 (95% CI: 0.92-3.59) | **Author’s conclusion:**  “Despite these limitations, our findings demonstrate that bariatric surgery in patients ≥70 years old results in substantial weight loss and co-morbidity remission and has an acceptable safety profile, with a serious complication rate of <5% and mortality rate similar to younger patients. As a result of this work, bariatric surgery programs and surgeons with self-imposed age limits should consider broadening their patient selection criteria to include patients ≥70 years old after careful evaluation to ensure their appropriateness for surgery.”  **Reviewer’s comments:**  This study shows that older age is significantly associated with increased risk of any complication, and haemorrhage, but not severe complications, infection, or obstruction, following RYGB. It also shows that older age is significantly associated with increased risk of any complication, severe complications, and leak or perforation, but not haemorrhage, or perforation, following SG.  This study has clearly met 7/9 (78%) criteria in the critical appraisal tool. |
| **Authors:**  Sheka, et al. [117]  **Year published:** 2019  **Study design:**  Cohort study  **Country:**  United States | **Sample size:**  N=108,198  **Inclusion criteria:**   - Patients who underwent either LRYGB or LSG between January 1, 2015 and December 31, 2015 - Patients who were either non-Hispanic black or non-Hispanic white   **Exclusion criteria:**   - Patients with missing data for age, BMI, or race/ethnicity   **Setting and population:**   - Patient records identified from the 2015 MBSAQIP PUF | - Race | - 30-day postoperative complications group and separately (ARF, cardiac arrest, deep incisional SSI, MI, respiratory failure, OS-SSI, pneumonia, progressive renal insufficiency, PE, sepsis, septic shock, superficial SSI, transfusion, unplanned intubation, UTI, DVT, wound disruption, unplanned ICU admission), separately for LRYGB and LSG - 30-day mortality, separately for LRYGB and LSG | **Statistical analysis:**   - Descriptive statistics - “A significance level of 0.05 was used for univariate analysis. A multivariate logistic regression model was created to determine the independent contribution of race to 30-day mortality.”   **Results/effect estimates:^a^**  1. LRYGB  Any complication (%)   - Black: 4.9% (304/6,249) - White: 4.9% (1,338/27,020) - P = 0.944   Superficial SSI (%)   - Black: 1.0% (60/6,249) - White: 1.0% (227/27,020) - P = 0.713   Required transfusion (%)   - Black: 1.3% (79/6,249) - White: 1.2% (338/27,020) - P = 0.847   UTI (%)   - Black: 0.4% (24/6,249) - White: 0.5% (144/27,020) - P = 0.151   Unplanned ICU admission (%)   - Black: 1.5% (94/6,249) - White: 1.4% (379/27,020) - P = 0.944   Death (%)   - Black: 0.2% (13/6,249) - White: 0.2% (47/27,020) - P = 0.538 - AOR not significant, p = 0.585   2. LSG  Any complication (%)   - Black: 2.2% (390/17,524) - White: 2.2% (1,280/57,405) - P = 0.973   Superficial SSI (%)   - Black: 0.2% (36/17,524) - White: 0.3% (167/57,405) - P = 0.057   Required transfusion (%)   - Black: 0.6% (100/17,524) - White: 0.6% (326/57,405) - P = 0.966   UTI (%)   - Black: 0.3% (48/17,524) - White: 0.3% (175/57,405) - P = 0.51   Unplanned ICU admission (%)   - Black: 0.7% (126/17,524) - White: 0.6% (359/57,405) - P = 0.176   Death (%)   - Black: 0.2% (31/17,524) - White: 0.1% (44/57,405) - P <0.001 - AOR = 3.613 (95% CI: 1.990-6.558, p <0.001) | **Author’s conclusion:**  “We found significant racial disparities in bariatric surgery outcomes, including higher mortality in black patients undergoing SG. The specific causes of these disparities remain unclear and must be the subject of future research.”  **Reviewer’s comments:**  This study shows that black race is not associated with increased rates of postoperative complications following both LRYGB and LSG. It also shows that black race is significantly associated with increased mortality rates following LSG, but not LRYGB.  This study has clearly met 7/9 (78%) criteria in the critical appraisal tool. |
| **Authors:**  Ozdas and Bozkurt[118]  **Year published:** 2019  **Study design:**  Cohort study  **Country:**  Turkey | **Sample size:**  N=130  **Inclusion criteria:**   - Patients who underwent LSG in a single centre due to morbid obesity between January 2014 and December 2017   **Exclusion criteria:**   - Patients who had underwent cholecystectomy before surgery - Patients who were found to have gallstones in the gallbladder in a preoperative ultrasound and underwent simultaneous cholecystectomy - Patients who were lost to follow-up   **Setting and population:**   - Not reported | - Age - Sex | 1. Gallstones detected on ultrasound at 12 months after surgery | **Statistical analysis:**   - Descriptive statistics - “An independent sample t test and a Mann-Whitney U test were used for the analysis of quantitative data. A Wilcoxon test was used in the analysis of quantitative data. A chi-square test was used in the analysis of qualitative data, and Fischer’s exact test was used when the requirements for chi-square were not met.”   **Results/effect estimates:**  1. Age (y)   - Cases Gallstones detected: median 42.0 - Controls Gallstones not detected: median 35.0 - P = 0.002   2. Sex   - Cases Gallstones detected: male 17.1% (6/35), female 22.1% (21/95) - Controls Gallstones not detected: male 82.9% (29/35), female 77.9% (74/95) - P = 0.536 | **Author’s conclusion:**  “The general risk factors for the development of gallstones were not found to be consistent with the factors in the patient population undergoing LSG. The present study identified no significant relationship between decreased BMI following LSG and the postoperative development of gallstones. Preoperative hypertension and coronary artery disease were found to be significantly related to the development of gallstones after surgery. The authors suggest that patients with preexisting CAD and hypertension in the preoperative period must be followed with ultrasound more meticulously.”  **Reviewer’s comments:**  This study shows that older age, but not sex, is significantly associated with increased rates of gallstones.  This study has clearly met 8/10 (80%) criteria in the critical appraisal tool. |
| **Authors:**  Nickel, et al. [119]  **Year published:** 2019  **Study design:**  Cohort study  **Country:**  Germany | **Sample size:**  N=180  **Inclusion criteria:**   - Patients who underwent RYGB or LSG between January 2006 and November 2014, with indication for obesity surgery according to SAGES, ASMBS, IFSO, and the German S3 guidelines, and a BMI ≥40 or 35-40 with major obesity-associated comorbidities   **Exclusion criteria:**   - Patients who underwent surgeries other than RYGB or LSG - Patients who underwent re-do surgery - Patients who underwent open surgery - Patients who were participating in concurring studies - Patients with insufficient information on follow-up, age of onset of obesity, and years of obesity - Patients with a BMI <35   **Setting and population:**   - Patient records identified from a prospectively collected database from the Department of General, Visceral and Transplant Surgery of the Heidelberg University Hospital | - Age - Sex | 1. Postoperative complications group | **Statistical analysis:**   - Descriptive statistics - “Multiple linear and logistic regression analyses were used to assess the influence of several independent variables”   **Results/effect estimates:**  1. Postoperative complications   - Age: OR = 1.001, p = 0.890 - Sex (female vs male): OR = 1.055, p = 0.369 | **Author’s conclusion:**  “Greater BMI was associated with a lower %EWL and age was associated with a low %TWL. YOO and AOO did not influence outcome. Age, BMI, and EOSS score were the most important predictors for risk and success after obesity surgery. Surgery should be performed early enough for optimal outcomes.”  **Reviewer’s comments:**  This study shows that older age, and female sex are not associated with increased risk of postoperative complications.  This study has clearly met 8/9 (89%) criteria in the critical appraisal tool. |
| **Authors:**  Nevo, et al. [120]  **Year published:** 2019  **Study design:**  Cohort study  **Country:**  Israel | **Sample size:**  N=131  **Inclusion criteria:**   - Patients who underwent SG between May 2010 and November 2015   **Exclusion criteria:**   - Patients with less than 6 months of follow-up   **Setting and population:**   - Patient records identified from a prospectively collected database from the bariatric surgery unit of the Tel-Aviv Sourasky medical centre | - Age | 1. Postoperative complications group and separately (early: leak/abscess, bleeding/haematoma, other, reoperation; late (>30 days): gastroesophageal reflux disease (GORD), dysphagia/stricture, postoperative ventral hernia) | **Statistical analysis:**   - Descriptive statistics - “For multivariate analysis the forward stepwise logistic regression technique was applied.”   **Results/effect estimates:**  1. Postoperative complications (%)  Bleeding/haematoma   - Age ≥65y: 3% (2/66) - Age <65y: 1.5% (1/65) - P = 0.562   Other   - Age ≥65y: 1.5% (1/66) - Age <65y: 1.5% (1/65) - P = 1   Re-operation   - Age ≥65y: 3% (2/66) - Age <65y: 1.5% (1/65) - P = 0.562   GORD   - Age ≥65y: 7.5% (5/66) - Age <65y: 4.6% (3/65) - P = 0.469   Dysphagia/stricture   - Age ≥65y: 4.5% (3/66) - Age <65y: 3% (2/65) - P = 0.651 | **Author’s conclusion:**  “In an elderly population, laparoscopic sleeve gastrectomy is safe and effective, yet weight loss outcomes are more modest when compared to a younger surgical population. Carefully selected elderly patients can benefit from bariatric surgery.”  **Reviewer’s comments:**  This study shows that older age is not associated with increased risk of postoperative complications.  This study has clearly met 7/9 (78%) criteria in the critical appraisal tool. |
| **Authors:**  Mocanu, et al. [121]  **Year published:** 2019  **Study design:**  Cohort study  **Country:**  Canada, United States | **Sample size:**  N=77,596  **Inclusion criteria:**   - Patients who underwent RYGB in 2015 and 2016   **Exclusion criteria:**   - Patients with prior or revisional surgery - Patients younger than 18 yrs - Patients who underwent emergency surgery   **Setting and population:**   - Patient records identified from the MBSAQIP database in 2015 and 2016 | - Age - Sex - Race | 1. Gastrointestinal leak after RYGB (requiring a drain placed for >30 days, readmission, intervention, reoperation, or death due to a leak) | **Statistical analysis:**   - Descriptive statistics - “A nonparsimonious multivariable logistic regression model was developed to determine predictive factors for development of leak.”   **Results/effect estimates:**  1. Age   - P = 0.081 - Age (per 10 yr): OR = 1.9 (95% CI: 1.2-3.0, p = 0.006)   18-30y   - Cases Gastrointestinal leak: 8.8% (42/476) - Controls No gastrointestinal leak: 10.3% (7,972/77,120)   30-40y   - Cases Gastrointestinal leak: 21.0% (100/476) - Controls No gastrointestinal leak: 24.1% (18,573/77,120)   40-50y   - Cases Gastrointestinal leak: 29.4% (140/476) - Controls No gastrointestinal leak: 29.0% (22,383/77,120)   50-60y   - Cases Gastrointestinal leak: 24.4% (116/476) - Controls No gastrointestinal leak: 23.9% (18,464/77,120)   >60y   - Cases Gastrointestinal leak: 16.4% (78/476) - Controls No gastrointestinal leak: 12.6% (9,728/77,120)   2. Sex (female)   - Cases Gastrointestinal leak: 74.8% (256/476) - Controls No gastrointestinal leak: 79.8% (61,512/77,120) - P = 0.007 - Female: OR = 0.86 (95% CI: 0.6-1.1, p = 0.284)   3. Race   - P = 0.09   White (Ref)   - Cases Gastrointestinal leak: 79.8% (380/476) - Controls No gastrointestinal leak: 75.9% (58,547/77,120)   Black   - Cases Gastrointestinal leak: 12.8% (61/476) - Controls No gastrointestinal leak: 14.1% (10,906/77,120) - OR = 1.4 (95% CI: 0.7-3.2, p = 0.369)   Other   - Cases Gastrointestinal leak: 7.4% (35/476) - Controls No gastrointestinal leak: 9.9% (7,667/77,120) - OR = 3.8 (95% CI: 0.9-15.4, p = 0.062) | **Author’s conclusion:**  “Using the robust MBSAQIP database, we found RYGB  to be a safe procedure with low morbidity and mortality. The overall leak rate was 0.6% with leak significantly increasing all other complications, readmission, and reoperation rates at 30 days. Logistic regression identified prior PE and partially dependent functional status as the 2 largest predictors of leak while increased albumin was the only protective factor. Optimizing preoperative nutrition and strength in these patients through structured multidisciplinary programs may therefore have a role in the ongoing improvement of outcomes after RYGB.”  **Reviewer’s comments:**  This study shows that older age, but not sex, or race, is significantly associated with increased risk of gastrointestinal leak.  This study has clearly met 9/10 (90%) criteria in the critical appraisal tool. |
| **Authors:**  Mocanu, et al. [122]  **Year published:** 2019  **Study design:**  Cohort study  **Country:**  Canada, United States | **Sample size:**  N=175,353  **Inclusion criteria:**   - Patients who underwent LSG in 2015 and 2016   **Exclusion criteria:**   - Patients with prior bariatric surgery - Patients younger than 18 yrs - Patients with bougie size <30 or >60 - Patients with pylorus distance <2 or >8 cm - Patients with missing bougie size or pylorus distance information - Patients who underwent emergency surgery   **Setting and population:**   - Patient records identified from the MBSAQIP database in 2015 and 2016 | - Age - Sex - Race | 1. Bleed after LSG (requiring reoperation, readmission, reintervention, or transfusion within the first 72 hrs of surgery, or death caused by bleeding) | **Statistical analysis:**   - Descriptive statistics - “A nonparsimonious multivariable logistic regression model was developed to determine predictive factors for the development of bleed.”   **Results/effect estimates:**  1. Age   - P <0.001 - Older age (per 10 yr): OR = 1.1 (95% CI: 1.03-1.16, p = 0.002)   18-30y   - Cases Bleed: 5.8% (65/1,116) - Controls Nonbleed: 11.9% (20,693/174,237)   30-40y   - Cases Bleed: 18.6% (207/1,116) - Controls Nonbleed: 25.8% (44,965/174,237)   40-50y   - Cases Bleed: 29.8% (332/1,116) - Controls Nonbleed: 29.0% (50,455/174,237)   50-60y   - Cases Bleed: 26.1% (291/1,116) - Controls Nonbleed: 22.0% (38,374/174,237)   >60y   - Cases Bleed: 19.8% (221/1,116) - Controls Nonbleed: 11.3% (19,750/174,237)   2. Sex (female)   - Cases Bleed: 75.5% (842/1,116) - Controls Nonbleed: 79.0% (137,637/174,237) - P = 0.004 - Female: OR = 1.07 (95% CI: 0.92-1.24, p = 0.4)   3. Race   - P = 0.118   White   - Cases Bleed: 72.4% (808/1,116) - Controls Nonbleed: 72.9% (126,998/174,237)   Black   - Cases Bleed: 20.3% (227/1,116) - Controls Nonbleed: 18.5% (32,284/174,237)   Other   - Cases Bleed: 7.3% (81/1,116) - Controls Nonbleed: 8.6% (14,955/174,237) | **Author’s conclusion:**  “Bleeding after LSG is associated with increased complications, readmission and reoperation rates, and mortality at 30 days. SLR techniques independently predict a lower risk of postoperative bleeding after LSG. Adoption of these techniques may therefore have an important role in reducing morbidity and mortality for patients who undergo LSG.”  **Reviewer’s comments:**  This study shows that older age, but not sex, or race, is significantly associated with increased risk of bleed.  This study has clearly met 9/10 (90%) criteria in the critical appraisal tool. |
| **Authors:**  Martin, et al. [123]  **Year published:** 2019  **Study design:**  Cohort study  **Country:**  Chile | **Sample size:**  N=216  **Inclusion criteria:**   - Patients who underwent bariatric surgery (SG, RYGB, sleeve gastrectomy with jejunal bypass (SGJB)) between 2006 and 2017 in a particular institution - All operated patients ≥60y for the case group, who were compared with a control group of patients ≤50y obtained randomly, paired by gender, BMI, T2DM, hypertension, dyslipidaemia, and surgical technique in a 1:2 ratio   **Exclusion criteria:**   - Patients between ages 51 and 59, who did not fit into any of the selected groups   **Setting and population:**   - Not reported | - Age | 1. Surgical complications 2. Mortality | **Statistical analysis:**   - Descriptive statistics - “Differences in categorical variables were analysed with Pearson chi-square, continuous variables with Student’s t test for variables with normal distribution, and Mann-Whitney U test for variables with non-normal distribution. The association analysis between the groups and surgical techniques was performed with chi-square, and the analysis of each technique in particular with Student’s t test.”   **Results/effect estimates:**  1. Surgical complications   - Age ≥60y: 2.8% (2/72) - Age <50y: 2.8% (4/144) - P = 1.000   2. Mortality   - Age ≥60y: 0% (0/72) - Age <50y: 0% (0/144) - P = 1.000 | **Author’s conclusion:**  “In a high-volume centre, the group of patients older than 60 years did not present a greater risk of morbidity and mortality than the control group. Also, no difference was found in 30 days of hospital readmissions. Probably, risk of conversion to open surgery is increased by a higher prevalence of previous laparotomies. To confirm these results, large numbered studies are needed. It is of interest to carry out studies with a long-term follow-up to evaluate the survival rate, weight loss, and remission of comorbidities in these patients.”  **Reviewer’s comments:**  This study shows that older age is not associated with increased risk of postoperative complications, or mortality.  This study has clearly met 6/9 (67%) criteria in the critical appraisal tool. |
| **Authors:**  Ivanics, et al. [124]  **Year published:** 2019  **Study design:**  Cohort study  **Country:**  United States, Canada | **Sample size:**  N=256,817  **Inclusion criteria:**   - Patients who underwent LSG or LRYGB from January 1, 2016 to December 31, 2017   **Exclusion criteria:**   - Patients undergoing emergent surgery, conversions, or revisions, who were aged <18y and without 30-day follow-up data   **Setting and population:**   - Patient records identified from the MBSAQIP database in 2016 and 2017 | - Age - Sex - Race - Hispanic | - Dehydration requiring treatment within 30 days after LSG or LRYGB | **Statistical analysis:**   - Descriptive statistics - “Univariate analysis used Pearson c2 test or Fischer’s exact test for categorical variables. To identify independent risk factors for the need for dehydration therapy, multivariable logistic regression was used to adjust for demographic, co-morbidity, and operative variables.”   **Results/effect estimates:**  1. Age  18-29y (Ref)   - Cases Dehydration treatment: 16.5% (1,578/9,592) - Controls No dehydration treatment: 11.4% (28,234/247,225)   30-39y   - Cases Dehydration treatment: 31.9% (3,056/9,592) - Controls No dehydration treatment: 25.4% (62,904/247,225) - AOR = 0.81 (95% CI: 0.76-0.86, p <0.05)   40-49y   - Cases Dehydration treatment: 27.2% (2,605/9,592) - Controls No dehydration treatment: 29.1% (72,097/247,225) - AOR = 0.57 (95% CI: 0.53-0.61, p <0.05)   50-59y   - Cases Dehydration treatment: 17.2% (1,654/9,592) - Controls No dehydration treatment: 22.4% (55,433/247,225) - AOR = 0.44 (95% CI: 0.41-0.48, p <0.05)   ≥60y   - Cases Dehydration treatment: 7.3% (699/9,592) - Controls No dehydration treatment: 11.6% (28,557/247,225) - AOR = 0.35 (95% CI: 0.32-0.39, p <0.05)   2. Sex  Female (Ref)   - Cases Dehydration treatment: 88.8% (8,521/9,592) - Controls No dehydration treatment: 79.2% (195,867/247,225)   Male   - Cases Dehydration treatment: 11.2% (1,071/9,592) - Controls No dehydration treatment: 20.8% (51,358/247,225) - AOR = 0.52 (95% CI: 0.49-0.56, p <0.05)   3. Race  White (Ref)   - Cases Dehydration treatment: 69.4% (6,657/9,592) - Controls No dehydration treatment: 73.0% (180,415/247,225)   Black   - Cases Dehydration treatment: 23.9% (2,294/9,592) - Controls No dehydration treatment: 17.3% (42,838/247,225) - AOR = 1.33 (95% CI: 1.26-1.40, p <0.05)   Other   - Cases Dehydration treatment: 1.1% (108/9,592) - Controls No dehydration treatment: 1.2% (2,929/247,225) - AOR = 0.95 (95% CI: 0.78-1.16, p >0.05)   Unknown/not reported   - Cases Dehydration treatment: 5.6% (533/9,592) - Controls No dehydration treatment: 8.5% (21,043/247,225) - AOR = 0.80 (95% CI: 0.72-0.88, p <0.05)   4. Hispanic  No (Ref)   - Cases Dehydration treatment: 83.1% (7,968/9,592) - Controls No dehydration treatment: 77.8% (192,293/247,225)   Yes   - Cases Dehydration treatment: 10.9% (1,049/9,592) - Controls No dehydration treatment: 12.7% (31,390/247,225) - AOR = 0.84 (95% CI: 0.79-0.90, p <0.05)   Unknown   - Cases Dehydration treatment: 6.0% (575/9,592) - Controls No dehydration treatment: 9.5% (23,542/247,225) - AOR = 0.69 (95% CI: 0.63-0.76, p <0.05) | **Author’s conclusion:**  “Dehydration is a strong risk factor for postoperative ED  visits and readmission after LSG or LRYGB. Patients at higher risk for requiring dehydration treatment include those undergoing LRYGB, LOS ≥3 days, younger, female, black, experience a postoperative complication, and have GERD, HTN, previous DVT, and chronic steroids/immunosuppression. Closer surveillance and proactive measures for patients with elevated risk may serve to mitigate and potentially prevent the development of postoperative dehydration and improve outcomes.”  **Reviewer’s comments:**  This study shows that younger age, female sex, black race, and non-Hispanic race are significantly associated with increased risk of dehydration requiring treatment.  This study has clearly met 8/10 (80%) criteria in the critical appraisal tool. |
| **Authors:**  Guzman, et al. [125]  **Year published:** 2019  **Study design:**  Cohort study  **Country:**  Chile | **Sample size:**  N=176  **Inclusion criteria:**   - Patients who underwent bariatric surgery (SG, SGJB, RYGB) between January and December 2014 in a Chilean institution - Patients with preoperative negative abdominal ultrasound for cholelithiasis and follow-up for at least 12 months   **Exclusion criteria:**   - Patients with previous cholecystectomy, sludge, stones, or gallbladder polyps recognised on abdominal ultrasound and documented follow-up of less than 12 months - Patients with simultaneous or deferred cholecystectomy   **Setting and population:**   - Not reported | - Age - Sex | 1. Cholelithiasis within 12 months after bariatric surgery | **Statistical analysis:**   - Descriptive statistics - “Multivariate analysis was performed using logistic regression to determine independent variables related to postbariatric” cholelithiasis   **Results/effect estimates:**  1. Cholelithiasis   - Age: OR = 1.02 (95% CI: 0.98-1.06, p >0.05) - Sex: OR = 1.75 (95% CI: 0.82-3.73, p >0.05) | **Author’s conclusion:**  “Incidence of CL [cholelithiasis] was up to one-third of the patients followed up for 12 months after BS. Excessive weight loss and other variables studied did not increase risk. Hypertension seems to be protective against gallstone formation, but this result needs further analysis.”  **Reviewer’s comments:**  This study shows that older age, and sex are not associated with increased risk of postoperative complications.  This study has clearly met 8/10 (80%) criteria in the critical appraisal tool. |
| **Authors:**  Goldberg, et al. [126]  **Year published:** 2019  **Study design:**  Cohort study  **Country:**  United States | **Sample size:**  N=37,634  **Inclusion criteria:**   - Patients who underwent non-revisional LRYGB and LSG procedures - Patients were matched into pairs for age 18-65y vs >65y for LSG and LRYGB separately   **Exclusion criteria:**   - Nil   **Setting and population:**   - Patient records identified from the 2015 to 2017 MBSAQIP database | - Age | - 30-day overall postoperative complications without death, for LSG and LRYGB separately - 30-day anastomotic/staple-line leak, for LSG and LRYGB separately - 30-day reoperation, for LSG and LRYGB separately - 30-day readmission, for LSG and LRYGB separately - 30-day reintervention, for LSG and LRYGB separately - 30-day mortality, for LSG and LRYGB separately | **Statistical analysis:**   - Descriptive statistics - “Based on matched samples, McNemar’s tests were carried out for all binary outcomes.”   **Results/effect estimates:^a^**  1. LSG  Any complication without death   - Age >65y: 3.58% (480/13,422) - Age 18-65: 2.56% (344/13,422) - Risk difference (RD) = 0.0101 (95% CI: 0.0059-0.0143, p <0.0001)   Anastomotic/staple-line leak   - Age >65y: 0.41% (55/13,422) - Age 18-65: 0.45% (60/13,422) - RD = -0.0004 (95% CI: -0.0019-0.0012, p = 0.7093)   30-day reoperation   - Age >65y: 1.08% (145/13,422) - Age 18-65: 0.94% (126/13,422) - RD = 0.0014 (95% CI: -0.0010-0.0038, p = 0.2742)   30-day readmission   - Age >65y: 3.53% (474/13,422) - Age 18-65: 3.51% (471/13,422) - RD = 0.0002 (95% CI: -0.0043-0.0047, p = 0.9481)   30-day reintervention   - Age >65y: 1.07% (144/13,422) - Age 18-65: 0.95% (128/13,422) - RD = 0.0012 (95% CI: -0.0012-0.0036, p = 0.3631)   Death   - Age >65y: 0.23% (31/13,422) - Age 18-65: 0.09% (12/13,422) - RD = 0.0014 (95% CI: 0.0005-0.0024, p = 0.0054)   2. LRYGB  Any complication without death   - Age >65y: 5.73% (309/5,395) - Age 18-65: 5.13% (277/5,395) - RD = 0.0059 (95% CI: -0.0029-0.0147, p = 0.2003)   Anastomotic/staple-line leak   - Age >65y: 1.11% (60/5,395) - Age 18-65: 1.06% (57/5,395) - RD = 0.0006 (95% CI: -0.0034-0.0045, p = 0.8534)   30-day reoperation   - Age >65y: 2.78% (150/5,395) - Age 18-65: 2.22% (120/5,395) - RD = 0.0056 (95% CI: -0.0004-0.0115, p = 0.0774)   30-day readmission   - Age >65y: 6.41% (346/5,395) - Age 18-65: 6.14% (331/5,395) - RD = 0.0028 (95% CI: -0.0067-0.0122, p = 0.5906)   30-day reintervention   - Age >65y: 2.30% (124/5,395) - Age 18-65: 2.63% (142/5,395) - RD = -0.0033 (95% CI: -0.0093-0.0026, p = 0.2972)   Death   - Age >65y: 0.46% (25/5,395) - Age 18-65: 0.28% (15/5,395) - RD = 0.0019 (95% CI: -0.0004-0.0042, p = 0.1539) | **Author’s conclusion:**  “Overall complication rates of bariatric surgery are low in patients >65 years. SG appears to have a favourable safety profile in this patient population compared with RYGB. The overall complication rate for RYGB is not significantly different between the older and younger groups.”  **Reviewer’s comments:**  This study shows that older age is significantly associated with increased risk of any complication without death, and mortality, but not anastomotic/staple-line leak, reoperation, readmission, or reintervention, following LSG. It also shows that older age is not associated with increased risk of postoperative complications, or mortality following LRYGB.  This study has clearly met 7/9 (78%) criteria in the critical appraisal tool. |
| **Authors:**  El Chaar, et al. [127]  **Year published:** 2019  **Study design:**  Cohort study  **Country:**  United States, Canada | **Sample size:**  N=101,599  **Inclusion criteria:**   - Patients who underwent GB or SG in 2015   **Exclusion criteria:**   - Patients with any surgical revisions or conversions - Patients with previous obesity or foregut surgery - Patients in emergency cases - Patients who underwent band procedures - Patients with a lack of 30-day follow-up data - Patients who underwent surgery types other than conventional laparoscopic   **Setting and population:**  Patient records identified from the 2015 MBSAQIP PUF | - Age - Sex | 1. Serious adverse events (SAEs) 30 days after surgery group (intervention, reoperation, drain, unplanned ICU admission, intraoperative or postoperative cardiac arrest requiring cardiopulmonary resuscitation, intraoperative or postoperative stroke/CVA, coma >24 hrs, deep incisional SSI, ARF requiring dialysis, PE, OS-SSI, intraoperative or postoperative transfusion within 72 hrs of surgery start time, intraoperative or postoperative MI, postoperative VTE requiring therapy, pneumonia, ventilator use >48 hrs, septic shock, anticoagulation initiated for presumed/confirmed VTE or PE) 2. Readmission 30 days after surgery | **Statistical analysis:**   - Descriptive statistics - Two separate direct multivariate logistic regression models were used for SAEs and readmissions, controlling for covariates   **Results/effect estimates:**   1. SAEs   Age (unit = 10):   - OR = 1.02 (95% CI: 0.99-1.05, p = 0.20)   Female gender:   - OR = 1.07 (95% CI: 0.99-1.16, p = 0.08)  1. 30-day readmissions   Age (ref = <55y)   - 55-65y: OR = 0.90, 95% CI: 0.82-0.98, p = 0.1) - ≥65y: OR = 1.03 (95% CI: 0.90-1.17, p = 0.68)   Female gender   - OR = 1.26 (95% CI: 1.16-1.36, p <0.0001) | **Author’s conclusion:**  “Our exploratory regression models may be used by clinicians to counsel patients  about surgical risks, although future external validation should occur in non-North American populations.”  **Reviewer’s comments:**  This study shows that older age, and female sex are not associated with increased risk of serious adverse events, while female sex, but not older age, is significantly associated with increased risk of readmission.  This study has clearly met 7/9 (78%) criteria in the critical appraisal tool. |
| **Authors:**  Doumouras, et al. [128]  **Year published:** 2019  **Study design:**  Cohort study  **Country:**  Canada | **Sample size:**  N=5,007  **Inclusion criteria:**   - Patients who underwent RYGB or SG within the province of Ontario for the purposes of weight loss between April 2009 and March 2012 - Patients over 18 yrs of age   **Exclusion criteria:**   - Nil   **Setting and population:**   - Patients identified from four bariatric Centres of Excellence (COEs) within the Ontario Bariatric Network in Canada | - Geographical location (distance from patients’ primary residence to the COE where their surgery was performed) | 1. 30-day postoperative complications group (surgical: anastomotic leak, haemorrhage, postoperative ileus, wound infection, death; medical: cardiac, respiratory, thrombotic complication, UTI, renal failure, ICU admission; general) | **Statistical analysis:**   - “Multivariable logistic regression was used to control for confounding in order to determine the extent to which the aforementioned exposures were associated with short-term overall complications and readmissions.”   **Results/effect estimates:**  1. Postoperative complications   - Distance (per 10 km): AOR = 1.00 (95% CI: 0.99-1.01, p = 0.747) - Resides in a local health integrated network that contains a COE: AOR = 1.10 (95% CI: 0.87-1.40, p = 0.434) - Resides in a neighbourhood classified as rural: AOR = 0.97 (95% CI: 0.77-1.23, p = 0.821) | **Author’s conclusion:**  “The COE model, where a few centers in high population areas service a large geographic region, is adequate in ensuring patients that live further away receive appropriate short-term care.”  **Reviewer’s comments:**  This study shows that geographical location is not associated with increased risk of postoperative complications.  This study has clearly met 7/9 (78%) criteria in the critical appraisal tool. |
| **Authors:**  Dang, et al. [129]  **Year published:** 2019  **Study design:**  Cohort study  **Country:**  Canada, United States | **Sample size:**  N=301,180  **Inclusion criteria:**   - All patients who underwent LRYGB and LSG in 2016 and 2017 - Patients who had elective and non-emergency operations   **Exclusion criteria:**   - Patients with previous bariatric surgery or coded as revisional bariatric surgery   **Setting and population:**   - Patient records identified from the MBSAQIP database between 2016 and 2017 | - Age - Sex - Race/ethnicity | 1. Clostridium difficile infection (CDI) within 30 days following bariatric surgery | **Statistical analysis:**   - Descriptive statistics - “Multivariable logistic regression analysis was used to determine predictive factors for the development of CDI within 30 days.”   **Results/effect estimates:**  1. Age (y)   - Cases CDI: mean 45(±13.0)y - Controls No CDI: mean 44.5(±12.0)y - P = 0.393   18-29:   - Cases CDI: 12.5% (48/383) - Controls No CDI: 11.9% (35,717/300,797)   30-39:   - Cases CDI: 28.5% (109/383) - Controls No CDI: 25.9% (77,594/300,797)   40-49:   - Cases CDI: 21.7% (83/383) - Controls No CDI: 28.9% (87,061/300,797)   50-59:   - Cases CDI: 24.0% (92/383) - Controls No CDI: 22.0% (66,025/300,797)   ≥60:   - Cases CDI: 13.3% (51/383) - Controls No CDI: 11.3% (33,869/300,797)   2. Female   - Cases CDI: 84.1% (322/383) - Controls No CDI: 79.6% (239,434/300,797) - P = 0.031 - AOR = 1.65 (95% CI: 1.24-2.20, p = 0.001)   3. Race/ethnicity  White   - Cases CDI: 81.7% (313/383) - Controls No CDI: 72.5% (218,194/300,797) - P <0.001 - AOR = 1.65 (95% CI: 1.27-2.14, p <0.001)   Black   - Cases CDI: 14.1% (54/383) - Controls No CDI: 17.9% (53,719/300,797)   Other   - Cases CDI: 4.2% (16/383) - Controls No CDI: 9.6% (28,884/300,797) | **Author’s conclusion:**  “The incidence of CDI following bariatric surgery remains lower than both the inpatient medical and general surgery populations. LRYGB had a higher risk of CDI compared to LSG, although the exact mechanism is unknown. Furthermore, CDI is associated with significant adverse outcomes post-operatively but had no increased risk of mortality.”  **Reviewer’s comments:**  This study shows that female sex, and white race, but not older age, are significantly associated with increased risk of CDI.  This study has clearly met 9/10 (90%) criteria in the critical appraisal tool. |
| **Authors:**  Dang, et al. [130]  **Year published:** 2019  **Study design:**  Cohort study  **Country:**  Canada, United States | **Sample size:**  N=274,221  **Inclusion criteria:**   - Patients who underwent primary LRYGB or LSG in 2015 and 2016   **Exclusion criteria:**   - Patients who underwent bariatric procedures other than LRYGB and LSG - Patients under 18 yrs of age - Patients who underwent revisional bariatric surgery - Patients who had previous bariatric or foregut surgery - Patients who underwent emergency surgery   **Setting and population:**   - Patient records identified from the MBSAQIP database between 2015 and 2016 | - Age - Sex - Race | 1. 30-day VTE (any of the following: DVT/PE confirmed on imaging requiring anticoagulation therapy, DVT/PE confirmed during autopsy, reoperation for DVT/PE, readmission for DVT/PE, reintervention for DVT/PE, death due to DVT/PE) | **Statistical analysis:**   - Descriptive statistics - “Multivariable logistic regression analysis was used to determine predictive factors for the development of VTE within 30 days.”   **Results/effect estimates:**  1. Age (y)   - Cases VTE: mean 45.5(±11.8)y - Controls No VTE: mean 44.6(±12.0)y - P = 0.084   18-29:   - Cases VTE: 9.3% (103/1,106) - Controls No VTE: 11.4% (31,134/273,115)   30-39:   - Cases VTE: 23.8% (263/1,106) - Controls No VTE: 25.3% (69,053/273,115)   40-49:   - Cases VTE: 31.2% (345/1,106) - Controls No VTE: 29.0% (79,106/273,115)   50-59:   - Cases VTE: 22.9% (253/1,106) - Controls No VTE: 22.6% (61,690/273,115)   ≥60:   - Cases VTE: 12.8% (142/1,106) - Controls No VTE: 11.8% (32,132/273,115)   2. Female   - Cases VTE: 75.5% (835/1,106) - Controls No VTE: 79.2% (216,307/273,115) - P = 0.003 - Male sex: AOR = 1.16 (95% CI: 1.00-1.34, p = 0.049)   3. Race   - P <0.001   White (Ref)   - Cases VTE: 68.3% (755/1,106) - Controls No VTE: 73.8% (201,596/273,115)   Black   - Cases VTE: 25.1% (278/1,106) - Controls No VTE: 17.2% (47,092/273,115) - AOR = 1.59 (95% CI: 1.38-1.83, p <0.001)   Other   - Cases VTE: 6.6% (73/1,106) - Controls No VTE: 8.9% (24,427/273,115) | **Author’s conclusion:**  “Bariatric surgery carries a low overall risk of VTE, however, in select populations, the risk is high. Despite the low risk, VTE remains a significant contributor to morbidity and mortality associated with bariatric surgery. Stratifying low- and high-risk populations for VTE after bariatric surgery using the BariClot tool allows for informed clinical decision-making and enables further research on different prophylactic measures for low- and high-risk populations.”  **Reviewer’s comments:**  This study shows that male sex, and black race, but not older age, are significantly associated with increased risk of VTE.  This study has clearly met 9/10 (90%) criteria in the critical appraisal tool. |
| **Authors:**  Clapp, et al. [131]  **Year published:** 2019  **Study design:**  Cohort study  **Country:**  United States | **Sample size:**  N=44,379  **Inclusion criteria:**   - Patients who underwent primary GB in 2015 - Patients over the age of 18   **Exclusion criteria:**   - Patients with previous obesity surgery or foregut surgery   **Setting and population:**   - Patient records identified from the 2015 MBSAQIP database | - Age - Sex - Race - Hispanic | - 30-day marginal ulcer following GB | **Statistical analysis:**   - Descriptive statistics - Overall incidence of ulcer was estimated along with the 95% CI using the binomial distribution - “The rare logistic regression was employed to determine factors associated with the occurrence of ulcer”   **Results/effect estimates:**  1. Age (y)   - Cases Ulcer present: mean 44.6(±11.6)y - Controls No ulcer present: mean 45.61(±11.9)y - P = 0.38   2. Sex  Female   - Cases Ulcer present: 80.0% (124/155) - Controls No ulcer present: 80.1% (35,402/44,224)   Male   - Cases Ulcer present: 20.0% (31/155) - Controls No ulcer present: 19.9% (8,822/44,224) - P = 0.94   3. Race  Black or African American (Ref)   - Cases Ulcer present: 20.0% (31/155) - Controls No ulcer present: 14.5% (6,432/44,224)   Others   - Cases Ulcer present: 0.6% (1/155) - Controls No ulcer present: 1.2% (546/44,224) - P = 0.63 - AOR = 0.67 (95% CI: 0.09-4.88, p = 0.69)   Unknown/not reported   - Cases Ulcer present: 1.3% (2/155) - Controls No ulcer present: 7.5% (3,320/44,224) - P = 0.011 - AOR = 0.18 (95% CI: 0.04-0.73, p = 0.017)   White   - Cases Ulcer present: 78.1% (121/155) - Controls No ulcer present: 76.7% (33,926/44,224) - P = 0.12 - AOR = 0.77 (95% CI: 0.51-1.14, p = 0.19)   4. Hispanic  No   - Cases Ulcer present: 85.8% (133/155) - Controls No ulcer present: 78.9% (34,911/44,224)   Unknown   - Cases Ulcer present: 6.5% (10/155) - Controls No ulcer present: 9.2% (4,070/44,224) - P = 0.23   Yes   - Cases Ulcer present: 7.7% (12/155) - Controls No ulcer present: 11.9% (5,243/44,224) - P = 0.12 | **Author’s conclusion:**  “Using the MBSAQIP database, anastomotic ulcers seem to occur rarely in the first month. The large majority are diagnosed and treated endoscopically with minimal need for surgical intervention. The risk of anastomotic ulcer was increased with increased BMI, need for PTC [percutaneous transluminal cardiac catheterisation], and history of DVT/PE.”  **Reviewer’s comments:**  This study shows that older age, male sex, and race are not significantly associated with increased risk of marginal ulcer.  This study has clearly met 9/10 (90%) criteria in the critical appraisal tool. |
| **Authors:**  Bhandari, et al. [132]  **Year published:** 2019  **Study design:**  Cohort study  **Country:**  India | **Sample size:**  N=368  **Inclusion criteria:**   - Patients aged ≥65y who underwent laparoscopic bariatric surgery (RYGB, OAGB, SG) at a single institution from January 2010 to December 2013 - Asia-Pacific patients with BMI >37.5 kg/m^2^ and patients with BMI >32.5 kg/m^2^ with two co-morbid conditions - An equal number of adult patients (aged 17-64y), who also underwent bariatric surgery in the same period at the same institution, were matched for surgery type   **Exclusion criteria:**   - Nil   **Setting and population:**   - Patient records identified from a prospectively maintained database of bariatric metabolic procedures at a single institution | - Age | - Postoperative complications (episodes of hypotension, bleed, re-exploration, leak, early mortality, port site, minor wound infection) | **Statistical analysis:**   - Descriptive statistics   **Results/effect estimates:**  1. Postoperative complications (%)  Episodes of hypotension   - Geriatric (age ≥65y): 1.1% (2/184) - Adult (age 17-64y): 0% (0/184)   Bleed   - Geriatric: 1.1% (2/184) - Adult: 0% (0/184)   Leak   - Geriatric: 0% (0/184) - Adult: 1.1% (2/184)   Re-exploration   - Geriatric: 1.1% (2/184) (bleeding) - Adult: 1.1% (2/184) (leaks)   Early mortality   - Geriatric: 0% (0/184) - Adult: 0.54% (2/184) (PE) | **Author’s conclusion:**  “Bariatric surgery could be safe and feasible in selected geriatric Indian patients over 65 years old. It does not result in more perioperative or short-term complications as compared to the adult group. The length of hospital stay is similar to that in adult patients. Long-term and prospective studies are needed to confirm these findings.”  **Reviewer’s comments:**  This study shows that older age is not associated with increased risk of postoperative complications.  This study has clearly met 6/10 (60%) criteria in the critical appraisal tool. |
| **Authors:**  Arnold, et al. [133]  **Year published:** 2019  **Study design:**  Cohort study  **Country:**  United States | **Sample size:**  N=53,533  **Inclusion criteria:**   - Patients with BMI ≥35 kg/m^2^ and age ≥40y who underwent LRYGB or LSG between 2010 and 2014   **Exclusion criteria:**   - Patients who underwent emergency surgery - Patients with BMI <35 kg/m^2^ - Patients aged <40y   **Setting and population:**   - Patient records identified from the ACS NSQIP PUF from 2010 to 2014 | - Age | 1. Minor postoperative complications (superficial SSI, wound dehiscence, progressive renal insufficiency, UTI, peripheral nerve injury, transfusions, DVT requiring therapy, thrombophlebitis) 2. Major postoperative complications (deep SSI, OS-SSI, PE, cardiac arrest, MI, sepsis, septic shock) 3. 30-day mortality | **Statistical analysis:**   - Descriptive statistics - Multivariate stepwise logistic regression analysis was used, controlling for clinically relevant covariables to generate ORs for factors associated with adverse outcomes   **Results/effect estimates:^d^**  1. Minor postoperative complications (%)   - Age 40-49y (Ref): 4.6% (816/24,014) - Age 50-59y: 5.3% (786/19,997), AOR = 1.01 (95% CI: 0.91-1.12) - Age 60-64y: 6.6% (280/5,743), AOR = 1.15 (95% CI: 0.99-1.33) - Age 65-69y: 6.9% (151/3,040), AOR = 1.18 (95% CI: 0.98-1.42) - Age ≥70y: 9.1% (48/739), AOR = 1.57 (95% CI: 1.15-2.15)   2. Major postoperative complications (%)   - Age 40-49y (Ref): 2.2% (394/24,014) - Age 50-59y: 2.6% (385/19,997), AOR = 1.01 (95% CI: 0.88-1.17) - Age 60-64y: 3.8% (163/5,743), AOR = 1.36 (95% CI: 1.12-1.65) - Age 65-69y: 4.2% (90/3,040), AOR = 1.43 (95% CI: 1.12-1.82) - Age ≥70y: 6.3% (33/739), AOR = 2.13 (95% CI: 1.46-3.09)   3. Mortality (%)   - Age 40-49y (Ref): 0.1% (20/24,014) - Age 50-59y: 0.2% (31/19,997), AOR = 1.45 (95% CI: 0.83-2.51) - Age 60-64y: 0.3% (16/5,743), AOR = 2.16 (95% CI: 1.13-4.15) - Age 65-69y: 0.3% (9/3,040), AOR = 2.28 (95% CI: 1.06-4.92) - Age ≥70y: 0.5% (4/739), AOR = 4.30 (95% CI: 1.57-11.73) | **Author’s conclusion:**  “The present study demonstrates increasing complications and mortality for older patients undergoing laparoscopic weight loss surgery. Elderly patients have significantly higher rates of many comorbidities than their younger counterparts. However, when controlling for comorbidities, age was found to be independently associated with major and minor complications in patients undergoing laparoscopic RYGB and SG, as well as with mortality in patients undergoing laparoscopic Roux-en-Y.”  **Reviewer’s comments:**  This study shows that older age is significantly associated with increased risk of minor and major postoperative complications, and mortality.  This study has clearly met 7/9 (78%) criteria in the critical appraisal tool. |
| **Authors:**  Almby and Edholm[134]  **Year published:** 2019  **Study design:**  Cohort study  **Country:**  Sweden | **Sample size:**  N=36,362  **Inclusion criteria:**   - Patients who underwent RYGB between 2007 and 2013   **Exclusion criteria:**   - Nil   **Setting and population:**   - Patient records identified from the SOReg between 2007 and 2013 | - Age | 1. Anastomotic strictures within 1 y of surgery | **Statistical analysis:**   - Descriptive statistics - “Risk factors with a p < 0.10 were entered into a multivariate logistic regression analysis.”   **Results/effect estimates:**  1. Anastomotic strictures (%)  Age (y)   - Cases Stricture: mean 46.4(±12.4)y - Controls No stricture: mean 40.9(±11.0)y - P <0.0001 - <30y (Ref): 18% (n = 6,248) - 30-40y: 29% (n = 10,501), OR = 1.1 (95% CI: 0.5-2.3) - 40-50y: 32% (n = 11,225), OR = 1.2 (95% CI: 0.6-2.6) - 50-60y: 18% (n = 6,406), OR = 1.9 (95% CI: 0.9-4.1) - >60y: 3.5% (n = 1,248), OR = 6.2 (95% CI: 2.7-14.3)   Sex   - Cases Stricture: 30% (30/101) - Controls No stricture: 24% (8,703/36,261) - P = 0.19 | **Author’s conclusion:**  “Most anastomotic strictures are diagnosed within 70 days of surgery and can be successfully treated with two dilations or less. Dilating a strictured gastrojejunostomy after RYGB entails a risk of perforation (3.8%). Late-presenting strictures are more resistant to dilation than early presenting.”  **Reviewer’s comments:**  This study shows that older age, but not sex, is significantly associated with increased risk of anastomotic strictures.  This study has clearly met 9/10 (90%) criteria in the critical appraisal tool. |
| **Authors:**  Tang, et al. [135]  **Year published:** 2018  **Study design:**  Cohort study  **Country:**  United States | **Sample size:**  N=105  **Inclusion criteria:**   - Patients aged 18-65y who underwent VSG at the Johns Hopkins Centre for Bariatric Surgery between April 2011 and February 2015   **Exclusion criteria:**   - Nil   **Setting and population:**   - Patient records identified from the Johns Hopkins Centre for Bariatric Surgery | - Age - Sex - Race | - Postoperative thiamine deficiency (defined as <78 nM within 1y of the VSG) | **Statistical analysis:**   - Descriptive statistics - “Any variables with a P <0.1 in the univariate analysis were placed into a multivariate logistic regression best-fit model that also controlled for age, alcohol usage, sex, race, year of operation, and preoperative BMI.”   **Results/effect estimates:**  1. Age   - Cases Thiamine deficient: median 39y - Controls Thiamine sufficient: median 43.5y - P = 0.11 - OR = 0.97 (95% CI: 0.92-1.03, p = 0.36)   2. Sex   - P = 0.53   Female (Ref)   - Cases Thiamine deficient: 81.5% (22/27) - Controls Thiamine sufficient: 75.6% (59/78)   Male   - Cases Thiamine deficient: 18.5% (5/27) - Controls Thiamine sufficient: 24.4% (19/78) - OR = 0.70 (95% CI: 0.20-2.42, p = 0.57)   3. Race   - P = 0.024   Caucasian (Ref)   - Cases Thiamine deficient: 22.2% (6/27) - Controls Thiamine sufficient: 52.6% (41/78)   African American   - Cases Thiamine deficient: 66.7% (18/27) - Controls Thiamine sufficient: 41.0% (32/78) - OR = 3.90 (95% CI: 1.25-12.21, p = 0.019)   Other minorities   - Cases Thiamine deficient: 11.1% (3/27) - Controls Thiamine sufficient: 6.4% (5/78) - OR = 11.18 (95% CI: 1.56-80.11, p = 0.016) | **Author’s conclusion:**  “We found an alarmingly high prevalence of thiamine  deficiency (25.7%) in postoperative SG patients. This was associated with African American and other minority ethnicity, as well as postoperative nausea and vomiting, but not with multivitamin compliance, perhaps due to a lack of accurate reporting and inadequate thiamine content in the vitamins. As such, a prospective trial with standardized supplementation and close follow-up is needed. Because thiamine deficiency can result in Wernicke encephalopathy along with other serious consequences, it is important to identify risk factors so that thiamine deficiency can be prevented or detected early in these high-risk individuals. Additionally, it is important that future studies ensure full vitamin intake adherence and uniform amounts of thiamine supplementation so that optimal thiamine intake can be determined in SG patients.”  **Reviewer’s comments:**  This study shows that African-American race, and other minorities, but not older age, or male sex, are significantly associated with increased risk of postoperative thiamine deficiency.  This study has clearly met 10/10 (100%) criteria in the critical appraisal tool. |
| **Authors:**  Sun, et al. [136]  **Year published:** 2018  **Study design:**  Cohort study  **Country:**  England | **Sample size:**  N=26,420  **Inclusion criteria:**   - Patients aged ≥18y who were diagnosed with obesity and underwent bariatric surgery (GB, LAGB, LSG, others) as a primary procedure in National Health Services (NHS) sites or NHS-funded sites between April 2006 and March 2012   **Exclusion criteria:**   - Nil   **Setting and population:**   - Patient records identified from the Hospital Episode Statistics Admitted Care database | - Age - Sex - Ethnicity - Geographical area - Index of multiple deprivation (IMD) (socioeconomic status) | 1. Postoperative complications | **Statistical analysis:**   - Descriptive statistics - “Logistic regression was used for a model of the binary readmission and complication outcome variables. For each outcome, adjusted regression models were derived using stepwise forward selection of candidate explanatory variables.”   **Results/effect estimates:**  1. Postoperative complications  Age (y)   - 35-44y: OR = 0.9731 (95% CI: 0.7927-1.1993) - 45-54y: OR = 0.9128 (95% CI: 0.7413-1.1280) - 55-64y: OR = 1.0035 (95% CI: 0.7876-1.2775) - 65+y: OR = 0.7490 (95% CI: 0.3903-1.3063)   Female sex   - OR = 1.0634 (95% CI: 0.9022-1.2588)   Non-Caucasian ethnic group   - OR = 0.7143 (95% CI: 0.5989-0.8484)   Geographical area   - North England: OR = 0.7805 (95% CI: 0.6294-0.9683) - Central England: OR = 1.1529 (95% CI: 0.9036-1.4685) - East England: OR = 1.1608 (95% CI: 0.8528-1.5726) - South England: OR = 1.0543 (95% CI: 0.8366-1.3280)   IMD   - 2^nd^ quintile: OR = 0.9726 (95% CI: 0.8039-1.1753) - 3^rd^ quintile: OR = 0.8932 (95% CI: 0.7192-1.1052) - 4^th^ quintile: OR = 0.8290 (95% CI: 0.6503-1.0497) - 5^th^ quintile (last deprived): OR = 0.9005 (95% CI: 0.6884-1.1672) | **Author’s conclusion:**  “Gastric bypass was the most frequently reported procedure in England across the observation period. While utilization across all procedure types increased between 2007 and 2010, overall uptake of bariatric surgery in England represents only a small proportion of the eligible population. Readmission and complication rates were lower for gastric banding relative to those for either gastric bypass or sleeve gastrectomy. The observed inpatient mortality rate was low across all procedure types.”  **Reviewer’s comments:**  This study shows that Caucasian ethnicity, but not older age, female sex, geographical area, or IMD, is significantly associated with increased risk of postoperative complications.  This study has clearly met 8/10 (80%) criteria in the critical appraisal tool. |
| **Authors:**  Schurner, et al. [137]  **Year published:** 2018  **Study design:**  Cohort study  **Country:**  Switzerland | **Sample size:**  N=711  **Inclusion criteria:**   - All patients who underwent a primary LRYGB at the University Hospital Zurich between January 2006 and December 2013   **Exclusion criteria:**   - Patients aged <18y - Patients that specifically declared their unwillingness to have their clinical data collected for research purposes   **Setting and population:**   - Patients records identified from the database of the bariatric surgery in the University Hospital Zurich, and their clinical databases (KISIM, Cistec, Zurich) | - Age - Sex | 1. 30-day anaesthesia-related complications group (intubation complications (unexpected difficult airway, secondary change of intubation technique, failed intubation), the need for re-intubation or delayed extubation, complications related to catheter insertion and positioning during surgery; medication-related adverse reactions, postoperative nausea and vomiting (PONV)), and PONV separately 2. 30-day surgical complications group (gastrointestinal complications (e.g. anastomotic stenosis or leak, incisional hernia, fluid collection, anastomotic ulcer), infections (wound infections, general infections, UTIs, pneumonia), bleeding complications, cardiovascular (tachycardia, MI), respiratory (pleural effusion), renal complications), and infections separately | **Statistical analysis:**   - Descriptive statistics - “To correct for potential confounders, a multivariable model was constructed. Covariates with a significant correlation in the univariate analyses (p < 0.1), as well as covariates of clinical interest were included in the model.”   **Results/effect estimates:**  1. Anaesthesia-related complications   - Age (>35y): OR = 0.55 (95% CI: 0.38-0.76, p <0.001)   2. PONV   - Age (>35y): OR = 0.53 (95% CI: 0.37-0.76, p <0.001) - Sex (female vs male): OR = 1.91 (95% CI: 1.30-2.87, p <0.001)   3. Surgical complications   - Age (>42y): OR = 0.85 (95% CI: 0.45-1.63, p = 0.6)   4. Infections   - Age (>41y): OR = 1.24 (95% CI: 0.84-1.82, p = 0.3) - Sex (female vs male): OR = 1.50 (95% CI: 0.99-2.32, p = 0.05) | **Author’s conclusion:**  “Roux-en-Y bariatric surgery has few anaesthetic complications, the most common is PONV. PONV is more common in younger patients and not more common with volatile anaesthetics. Major complications are overall rare and occur in patients with lower BMI and longer surgery, likely reflecting more difficult procedures.”  **Reviewer’s comments:**  This study shows that younger age is significantly associated with increased risk of anaesthesia-related complications, PONV, but not surgical complications, or infections. It also shows that female sex is significantly associated with increased risk of PONV, and infections.  This study has clearly met 8/10 (80%) criteria in the critical appraisal tool. |
| **Authors:**  Moon, et al. [138]  **Year published:** 2018  **Study design:**  Case-control study  **Country:**  United States | **Sample size:**  N=44  **Inclusion criteria:**   - Patients who underwent LSG between January 1, 2008 and September 30, 2016 - Patients who developed portomesenteric vein thrombosis (PMVT) after LSG - “For each patient, 3 controls were selected using caliper-matching age by 5 years, caliper-matching preoperative BMI by 5 kg/m^2^, and exact matching by sex and center.”   **Exclusion criteria:**   - Nil   **Setting and population:**   - Patient records identified from a prospectively maintained database of 5,538 patients at 5 bariatric centres in the United States | - Sex - Age | - Portomesenteric vein thrombosis (PMVT) | **Statistical analysis:**   - Descriptive statistics - “Conditional logistic regression model was fitted to accurately analyse the matched data, history of cancer, current smoking status, diabetes status, and levothyroxine use as exposure variables.”   **Results/effect estimates:**  1. Sex   - P >0.99   Female   - Cases PMVT: 72.7% (8/11) - Controls No PMVT: 72.7% (24/33)   Male   - Cases PMVT: 27.3% (3/11) - Controls No PMVT: 27.3% (9/33)   2. Age (y)   - Cases PMVT: mean 49.9(±11.5)y - Controls No PMVT: mean 50.8(±10.8)y - P = 0.82 | **Author’s conclusion:**  “Incidence of PMVT is low after SG. A personal history  of malignancy and type 2 diabetes increase the risk of PMVT. Increasing abdominal pain in a context of dehydration is common presenting symptoms with diagnosis confirmed by CT. Anticoagulation is the standard treatment. There was no mortality associated with the occurrence of PMVT in this cohort.”  **Reviewer’s comments:**  This study shows that female sex and older age are not associated with increased risk of PMVT.  This study has clearly met 9/10 (90%) criteria in the critical appraisal tool. |
| **Authors:**  McCracken, et al. [139]  **Year published:** 2018  **Study design:**  Cohort study  **Country:**  United States | **Sample size:**  N=2,116  **Inclusion criteria:**   - Patients aged ≥18y with a BMI >35 kg/m^2^ who underwent RYGB between January 1, 2004 and December 31, 2014   **Exclusion criteria:**   - Patients without preRYGB assessment of haemoglobin (HbA1C) - Patients without postRYGB HbA1C levels   **Setting and population:**   - Patient records identified from a prospectively maintained clinical registry at a tertiary nutrition and weight management centre | - Age | 1. Anaemia within 8 years after RYGB (mild: female HbA1C 11–11.9 g/dL, male HbA1C 11–12.9 g/dL; moderate: female and male HbA1C 8–10.9 g/dL; severe: female and male HbA1C <8 g/dL) | **Statistical analysis:**   - Descriptive statistics - “Predictors of severe anaemia in the RYGB cohort were evaluated using Cox regression and identifying patient characteristics (e.g., age, sex, BMI, preRYGB iron status, early postRYGB weight loss) that were associated with postRYGB severe anaemia.”   **Results/effect estimates:**  1. Severe anaemia in the RYGB cohort   - Age ≥40y: HR = 2.97 (95% CI: 1.14-7.75, p = 0.026) | **Author’s conclusion:**  “The long-term incidence of clinically significant anaemia after RYGB is alarmingly high and warrants more detailed study.”  **Reviewer’s comments:**  This study shows that older age is significantly associated with increased risk of severe anaemia.  This study has clearly met 9/10 (90%) criteria in the critical appraisal tool. |
| **Authors:**  Major, et al. [140]  **Year published:** 2018  **Study design:**  Cohort study  **Country:**  Poland | **Sample size:**  N=788  **Inclusion criteria:**   - Patients who gave informed consent to participate in the study and met the eligibility criteria for bariatric treatment, either for LSG or LRYGB (BMI ≥35 kg/m^2^ in patients in whom surgically induced weight loss can result in the improvement of obesity-related diseases, or BMI ≥40 kg/m^2^)   **Exclusion criteria:**   - Patients who had previous bariatric procedures - Patients who lacked necessary data   **Setting and population:**   - Patient records from the prospective databases of bariatric patients in two academic hospitals in Poland | - Age | 1. Early postoperative complications (<30 days) group and separately (biochemical rhabdomyolysis, gastrointestinal leakage, gastrointestinal stricture, postoperative haemorrhage, wound infection) 2. Late postoperative complications (>30 days) group and separately (gastrointestinal stricture, port site hernia, marginal ulcer) | **Statistical analysis:**   - Descriptive statistics - “The influence of age group on postoperative complications, gastrointestinal leakage, gastrointestinal stricture, postoperative hemorrhage, wound infection, port site herniation, marginal ulcer and readmission rates was analyzed in univariate logistic regression models, and then in a multivariate model adjusting for relevant intergroup baseline differences and surgeries at two centers.”   **Results/effect estimates:**  1. Early postoperative complications (%)  Overall   - Age ≥50y: 13.21% (28/212) - Age <50y: 12.85% (74/576) - P = 0.894 - AOR = 1.03 (95% CI: 0.65-1.64, p = 0.892)   Clavien-Dindo III-V   - Age ≥50y: 8.02% (17/212) - Age <50y: 8.68% (50/576) - P = 0.768 - AOR = 0.92 (95% CI: 0.52-1.63, p = 0.768)   Clavien-Dindo I-II   - Age ≥50y: 5.19% (11/212) - Age <50y: 3.82% (22/576) - P = 0.395 - AOR = 1.38 (95% CI: 0.66-2.90, p = 0.397)   2. Late postoperative complications (%)  Overall   - Age ≥50y: 4.72% (10/212) - Age <50y: 2.26% (13/576) - P = 0.114 - AOR = 2.14 (95% CI: 0.92-4.97, p = 0.075)   Clavien-Dindo III-V   - Age ≥50y: 4.25% (9/212) - Age <50y: 1.74% (10/576) - P = 0.042 - AOR = 2.52 (95% CI: 1.01-6.30, p = 0.048) | **Author’s conclusion:**  “Bariatric surgery is safe and feasible in patients over 50 years old. The weight loss effect can be worse among patients over 50 years old; nevertheless, the treatment should be considered as effective.”  **Reviewer’s comments:**  This study shows that older age is significantly associated with increased risk of late Clavien-Dindo III-V complications, but not early overall, early Clavien-Dindo III-V, early Clavien-Dindo I-II, or late overall complications.  This study has clearly met 8/10 (80%) criteria in the critical appraisal tool. |
| **Authors:**  Mackay, et al. [141]  **Year published:** 2018  **Study design:**  Cohort study  **Country:**  New Zealand | **Sample size:**  N=1,362  **Inclusion criteria:**   - Adult patients who underwent LRYGB over a 12-year period between 8 March 2000 and 18 December 2012 - Patients who had attended up to 1 year of follow-up clinic   **Exclusion criteria:**   - Nil   **Setting and population:**   - Patient records identified from a prospectively maintained database from a single private centre in New Zealand | - Age | 1. Postoperative complications group (including but not limited to: cholecystitis, gastrojejunostomy stricture, internal hernia, incisional hernia, haemorrhage, abscess) 2. 30-day mortality | **Statistical analysis:**   - Descriptive statistics - “Comparisons of categorical variables were undertaken using chi-square test”   **Results/effect estimates:**  1. Postoperative complications (%)  Overall   - Age ≥60y: 6.7% (7/105) - Age <60y: 7.5% (94/1,257) - P = 0.7605   Multiple complications   - Age ≥60y: 2.9% - Age <60y: 0.7%   Symptomatic cholelithiasis   - Age ≥60y: 1.9% - Age <60y: 3.5%   2. 30-day mortality   - Age ≥60y: 0% (95% CI: 0-0.03) - Age <60y: 0% (95% CI: 0-3.5) | **Author’s conclusion:**  “LRYGB is an effective weight loss operation in the <60s and ≥60s. LRYGB is safe, with a low complication rate and 30-day postoperative mortality rate. LRYGB should not be restricted on the basis of age alone.”  **Reviewer’s comments:**  This study shows that older age is not associated with increased risk of postoperative complications, or mortality.  This study has clearly met 9/10 (90%) criteria in the critical appraisal tool. |
| **Authors:**  Koh, et al. [142]  **Year published:** 2018  **Study design:**  Cohort study  **Country:**  United States | **Sample size:**  N=41,475  **Inclusion criteria:**   - Severely obese patients aged ≥18y who underwent LRYGB or LSG   **Exclusion criteria:**   - Patients who underwent emergent cases, open cases, converted cases, or revisional cases   **Setting and population:**   - Patient records identified from the 2011 to 2015 ACS-NSQIP clinical database | - Age | - Serious morbidity (sepsis, pneumonia, CVA, respiratory failure, postoperative bleeding, anastomotic leak, ARF, cardiac complications, DVT, PE), for LRYGB and LSG separately - 30-day mortality, for LRYGB and LSG separately | **Statistical analysis:**   - Descriptive statistics - “Multivariate analysis was performed using linear regression for quantitative outcomes and logistic regression for dichotomous outcomes. Risk adjustments were made for covariates including patient characteristics and comorbidities.”   **Results/effect estimates:^a^**  1. LRYGB  Serious morbidity (%)   - Age ≥65y (n = 2,010): 5.7% - Age 18-64y (n = 39,465): 3.4% - OR = 1.43 (95% CI: 1.16-1.76, p = 0.001)   30-day mortality (%)   - Age ≥65y: 0.20% - Age 18-64y: 0.14% - OR = 0.8 (95% CI: 0.28-2.34, p = 0.688)   2. LSG  Serious morbidity (%)   - Age ≥65y (n = 2,055): 4.0% - Age 18-64y (n = 42,495): 2.2% - OR = 1.44 (95% CI: 1.12-1.84, p = 0.005)   30-day mortality (%)   - Age ≥65y: 0.29% - Age 18-64y: 0.07% - OR = 3.62 (95% CI: 1.34-9.83, p = 0.011) | **Author’s conclusion:**  “Overall, bariatric surgery is safe in the elderly population with a 30-day mortality rate of <0.3 per cent. This mortality rate in the elderly is similar between bariatric procedures (LRYGB vs LSG). As expected, bariatric surgery in the elderly is associated with higher morbidity compared with the nonelderly patient population. Therefore, elderly patients should be counselled regarding their higher risk of serious morbidity, but should not be denied for bariatric surgery based solely on their age.”  **Reviewer’s comments:**  This study shows that older age is significantly associated with increased risk of serious morbidity, but not mortality, following LRYGB. It also shows that older age is significantly associated with increased risk of serious morbidity, and mortality following LSG.  This study has clearly met 7/9 (78%) criteria in the critical appraisal tool. |
| **Authors:**  Kochkodan, et al. [143]  **Year published:** 2018  **Study design:**  Cohort study  **Country:**  United States | **Sample size:**  N=61,708  **Inclusion criteria:**   - Patients who underwent primary bariatric surgery (RYGB, LAGB, SG, BPD-DS) from 2006 to 2016   **Exclusion criteria:**   - Nil   **Setting and population:**   - Patient records identified from the MBSC database | - Sex | 1. 30-day postoperative complications group and separately (serious: abdominal abscess, bowel obstruction/hernia, leak, bleeding that requires a blood transfusion of more than 4 units, band-related problems, respiratory failure, renal failure, wound infection/dehiscence, VTE, MI or cardiac arrest, renal failure requiring long-term dialysis, respiratory failure requiring more than 7 days intubation or tracheostomy; surgical: leak/perforation, obstruction, infection, haemorrhage; medical: VTE, cardiac or respiratory problems, renal failure) | **Statistical analysis:**   - Descriptive statistics - “The 30-day complication outcomes were adjusted for males and females for procedure type, approach, patient characteristics, patient comorbidities, and clustering within the different MBSC sites.”   **Results/effect estimates:**  1. 30-day postoperative complications (%)  Any complication (%)   - Female (n = 48,172): 7.14% - Male (n = 13,536): 7.11% - OR = 0.99 (95% CI: 0.89-1.09, p = 0.7988)   Serious complication (%)   - Female: 2.12% - Male: 2.67% - OR = 1.28 (95% CI: 1.12-1.47, p = 0.0004)   Obstruction (%)   - Female: 1.38% - Male: 1.10% - OR = 0.77 (95% CI: 0.59-1.01, p = 0.0588)   Infection (%)   - Female: 1.99% - Male: 2.05% - OR = 1.03 (95% CI: 0.89-1.19, p = 0.6965)   Haemorrhage (%)   - Female: 1.62% - Male: 1.69% - OR = 1.04 (95% CI: 0.90-1.19, p = 0.6272) | **Author’s conclusion:**  “Despite significantly lower weight loss and increased complication rates, males tend to have markedly higher satisfaction and psychological well-being scores than females. To improve outcomes in males, earlier referral to surgery may help to significantly reduce their risk. Conversely, increased attention to psychological support in the perioperative period for females may lead to improved psychological outcomes (i.e., body image, depression, psychological well-being).”  **Reviewer’s comments:**  This study shows that male sex is significantly associated with increased risk of serious complications, but not any complication, infection, or haemorrhage. Although not statistically significant, rates of obstruction tended to be higher in female patients.  This study has clearly met 7/10 (70%) criteria in the critical appraisal tool. |
| **Authors:**  Kaplan, et al. [144]  **Year published:** 2018  **Study design:**  Cohort study  **Country:**  Canada | **Sample size:**  N=3,166  **Inclusion criteria:**   - Adult patients who underwent either LRYGB or LSG between January 2010 and May 2013 with complete 1-year follow-up   **Exclusion criteria:**   - Patients who underwent laparoscopic duodenal switch   **Setting and population:**   - Patient records identified from the Ontario Bariatric Registry database | - Age | 1. Postoperative complications separately (medical: persistent diarrhoea, pulmonary complications, cardiac complications, stroke, nutritional deficiency; surgical: anastomotic leak, DVT/PE, hernia, bowel obstruction, erosion/ulceration, bleeding, wound infection) | **Statistical analysis:**   - Descriptive statistics - “Univariate analysis of patients under 60 years of age versus those 60 years and older was done using the chi-square test or Fisher’s exact test for categorical variables and student’s t test for continuous variables as appropriate.” - Logistic regression analysis was done on 1-year postoperative complications, adjusting for baseline and perioperative characteristics   **Results/effect estimates:**  1. Postoperative complications (%)  Sleeve or anastomotic leak   - Age >60y: 0.5% (1/204) - Age ≤60y: 0.5% (17/2,962) - P >0.05   Internal hernia   - Age >60y: 0.5% (1/204) - Age ≤60y: 0.6% (20/2,962) - P >0.05   Sleeve or anastomotic stenosis   - Age >60y: 0.5% (1/204) - Age ≤60y: 1.8% (55/2,962) - P >0.05   Persistent diarrhoea   - Age >60y: 0.5% (1/204) - Age ≤60y: 0.7% (22/2,962) - P >0.05   Nutritional deficiency   - Age >60y: 2.9% (6/204) - Age ≤60y: 2.5% (75/2,962) - P >0.05   2. 1-year postoperative complications   - OR = 1.11 (95% CI: 0.31-3.94, p = 0.865) | **Author’s conclusion:**  “The older cohort who underwent LRYGB or LSG was at no greater risk for intra-operative and post-operative complications and showed greater reduction in medication use post-surgery when compared to the younger cohort.”  **Reviewer’s comments:**  This study shows that older age is not associated with increased risk of postoperative complications.  This study has clearly met 6/9 (67%) criteria in the critical appraisal tool. |
| **Authors:**  Hussan, et al. [145]  **Year published:** 2018  **Study design:**  Cohort study  **Country:**  United States | **Sample size:**  N=375,224  **Inclusion criteria:**   - Morbidly obese patients (BMI ≥35 kg/m^2^) who underwent elective laparoscopic RYGB, VSG, or hernia repair (controls)   **Exclusion criteria:**   - Patients aged <18y - Patients who were pregnant - Patients with abdominal malignancy - Patients with hernia surgery performed for indications other than hernia treatment - Patients with a hernia with gangrene or an obstruction - Patients with previous bariatric surgery - Patients with a diagnosis of chronic pancreatitis or pancreatic cysts - Patients who underwent open, emergent surgery or multiple surgeries - Patients with mortality or acute pancreatitis on index surgery admission - Patients without an admission length of stay reported   **Setting and population:**   - Patient records identified from the 2012-2014 National Readmission Database | - Age - Sex - Insurance status | - Acute pancreatitis within 6 months following VSG and RYGB, separately | **Statistical analysis:**   - Descriptive statistics - “Risk factors for AP admission within 6 months post-surgery were assessed by weighted logistic regression for VSG and RYGB separately. Univariable models were fit using all candidate risk factors followed by a multivariable model, which included all risk factors with p < 0.1 in the univariable analysis.”   **Results/effect estimates:^a^**  1. VSG  Age (y)   - P = 0.005 - 18-29: OR = 3.76 (95% CI: 1.68-8.45, p = 0.001) - 30-49: OR = 1.87 (95% CI: 0.99-3.56, p = 0.06) - ≥50: Ref   Sex: Female vs male   - OR = 1.99 (95% CI: 1.04-3.80, p = 0.04)   Insurance status   - P = 0.02 - Medicare: OR = 2.50 (95% CI: 1.20-5.19, p = 0.01) - Medicaid: OR = 0.72 (95% CI: 0.37-1.38, p = 0.32) - Self-pay: OR = 0.14 (95% CI: 0.02-0.97, p = 0.047) - Other: OR = 0.65 (95% CI: 0.09-4.73, p = 0.67) - Private insurance: Ref   2. RYGB  Age (y)   - P <0.001 - 18-29: OR = 6.40 (95% CI: 2.49-16.46, p = 0.001) - 30-49: OR = 3.04 (95% CI: 1.50-6.15, p = 0.002) - ≥50: Ref | **Author’s conclusion:**  “More patients develop AP within 6 months after VSG compared to RYGB and controls. This risk is highest for younger patients and those with gallstones. Prospective studies examining mechanisms and prevention are warranted.”  **Reviewer’s comments:**  This study shows that younger age, female sex, and having Medicare insurance are significantly associated with increased risk of acute pancreatitis, while self-pay status is significantly associated with decreased risk following VSG. It also shows that younger age is significantly associated with increased risk of acute pancreatitis following RYGB.  This study has clearly met 7/9 (78%) criteria in the critical appraisal tool. |
| **Authors:**  Hennings, et al. [146]  **Year published:** 2018  **Study design:**  Cohort study  **Country:**  United States | **Sample size:**  N=132,342  **Inclusion criteria:**   - Adult (≥18y) inpatients with a BMI ≥25 kg/m^2^ who underwent bariatric surgery   **Exclusion criteria:**   - Nil   **Setting and population:**   - Patient records identified from the Nationwide Inpatient Sample Database for the years 2003-2010 | - Age - Sex - Insurance status | 1. Postoperative complications group (cardiovascular, pulmonary, urinary, bleeding, infectious, wound complications) | **Statistical analysis:**   - Descriptive statistics - “Cross-tabulation and chi-square tests were used to examine the association between each of the independent factors and the outcome of interest. Factors with significant association were considered possible confounders and were included in multivariate logistic regression models.”   **Results/effect estimates:**  1. Postoperative complications (%)  Age (y)   - <35 (Ref): 4.2% - 35-<65: 6.6%, AOR = 1.38 (95% CI: 1.26-1.52, p <0.001) - ≥65: 12.2%, AOR = 1.48 (95% CI: 1.20-1.84, p <0.001)   Sex   - Male (Ref): 7.7% - Female: 5.9%, AOR = 0.81 (95% CI: 0.74-0.88, p <0.001)   Insurance status   - Private (Ref): 5.7% - Medicare: 11.6%, AOR = 1.54 (95% CI: 1.33-1.78, p <0.001) - Medicaid: 6.5%, AOR = 1.31 (95% CI: 1.08-1.60, p = 0.007) - Self-pay: 4.8%, AOR = 0.81 (95% CI: 0.66-0.997, p = 0.047) | **Author’s conclusion:**  “Publicly insured patients are significantly less likely to undergo bariatric surgery. As a group, these patients experience higher rates of obesity and related complications and thus are most in need of bariatric surgery.”  **Reviewer’s comments:**  This study shows that older age, male sex, and having Medicare or Medicaid insurance are significantly associated with increased risk of postoperative complications, while self-pay status is significantly associated with decreased risk.  This study has clearly met 5/9 (56%) criteria in the critical appraisal tool. |
| **Authors:**  Haskins, et al. [147]  **Year published:** 2018  **Study design:**  Cohort study  **Country:**  United States | **Sample size:**  N=266,544  **Inclusion criteria:**   - Adult (≥18y) patients who underwent elective LRYGB or LSG in 2015   **Exclusion criteria:**   - Patients who underwent emergency surgery, open RYGB, open SG, or revisional bariatric surgery - Patients aged <18y - Patients without 30-day follow-up data available   **Setting and population:**   - Patient records identified from the 2015 MBSAQIP database | - Age | 1. 30-day postoperative complications separately (cardiac events (MI, cardiac arrest requiring CPR), pulmonary events (prolonged intubation, reintubation, pneumonia), wound events (superficial SSI, deep SSI, OS-SSI, wound dehiscence), septic events (sepsis, septic shock), renal events (progressive renal insufficiency requiring dialysis), clotting events (DVT, PE)) 2. 30-day related unplanned reintervention (endoscopic or surgical) 3. 30-day related mortality 4. Composite morbidity and mortality outcome | **Statistical analysis:**   - Descriptive statistics - “Multivariate logistic regression was performed, adjusting for gender and race only since these variables could have functioned as confounders for the association of age with the 30-day morbidity and mortality outcomes of interest. Age quintile one (i.e., the youngest 20% of patients) was used as the reference group and 30-day morbidity, and mortality outcomes were compared between age quintile one and age quintiles two through five using the entire cohort of patients, the RNGYB and SG groups separately, and between the RNGYB and SG groups.”   **Results/effect estimates:**  1. 30-day postoperative complications (%)  Pulmonary events   - Quintile 1 (18-33y): Ref - Quintile 2 (34-41y): AOR = 1.08 (95% CI: 0.86-1.37, p = 0.50) - Quintile 3 (42-47y): AOR = 1.26 (95% CI: 1.00-1.57, p = 0.05) - Quintile 4 (48-56y): AOR = 1.60 (95% CI: 1.29-1.99, p <0.0001) - Quintile 5 (>56y): AOR = 2.87 (95% CI: 2.36-3.50, p <0.0001)   Wound events   - Quintile 1: Ref - Quintile 2: AOR = 1.10 (95% CI: 0.89-1.36, p = 0.38) - Quintile 3: AOR = 1.32 (95% CI: 1.08-1.61, p = 0.007) - Quintile 4: AOR = 1.37 (95% CI: 1.12-1.67, p = 0.003) - Quintile 5: AOR = 1.42 (95% CI: 1.16-1.73, p = 0.001)   Septic events   - Quintile 1: Ref - Quintile 2: AOR = 1.14 (95% CI: 0.82-1.57, p = 0.44) - Quintile 3: AOR = 1.55 (95% CI: 1.15-2.09, p = 0.004) - Quintile 4: AOR = 1.53 (95% CI: 1.13-2.07, p = 0.006) - Quintile 5: AOR = 2.28 (95% CI: 1.72-3.02, p <0.0001)   Clotting events   - Quintile 1: Ref - Quintile 2: AOR = 0.96 (95% CI: 0.76-1.21, p = 0.73) - Quintile 3: AOR = 1.18 (95% CI: 0.95-1.47, p = 0.14) - Quintile 4: AOR = 1.07 (95% CI: 0.85-1.35, p = 0.55) - Quintile 5: AOR = 1.14 (95% CI: 0.91-1.43, p = 0.25)   Renal events   - Quintile 1: Ref - Quintile 2: AOR = 1.23 (95% CI: 0.76-2.01, p = 0.40) - Quintile 3: AOR = 2.68 (95% CI: 1.75-4.09, p <0.0001) - Quintile 4: AOR = 3.11 (95% CI: 2.04-4.72, p <0.0001) - Quintile 5: AOR = 5.77 (95% CI: 3.88-8.56, p <0.0001)   2. 30-day related unplanned reintervention   - Quintile 1: Ref - Quintile 2: AOR = 1.02 (95% CI: 0.92-1.13, p = 0.69) - Quintile 3: AOR = 0.98 (95% CI: 0.88-1.08, p = 0.66) - Quintile 4: AOR = 0.87 (95% CI: 0.78-0.96, p = 0.009) - Quintile 5: AOR = 0.93 (95% CI: 0.84-1.03, p = 0.18)   3. 30-day related mortality   - Quintile 1: Ref - Quintile 2: AOR = 1.08 (95% CI: 0.48-2.46, p = 0.85) - Quintile 3: AOR = 1.11 (95% CI: 0.50-2.47, p = 0.81) - Quintile 4: AOR = 2.08 (95% CI: 1.01-4.27, p = 0.04) - Quintile 5: AOR = 3.31 (95% CI: 1.68-6.50, p = 0.0005)   4. Composite morbidity and mortality outcome   - Quintile 1: Ref - Quintile 2: AOR = 1.09 (95% CI: 1.01-1.17, p = 0.02) - Quintile 3: AOR = 1.15 (95% CI: 1.07-1.23, p = 0.0002) - Quintile 4: AOR = 1.21 (95% CI: 1.13-1.30, p <0.0001) - Quintile 5: AOR = 1.51 (95% CI: 1.41-1.62, p <0.0001) | **Author’s conclusion:**  “Older patients and patients who undergo Roux-en-Y gastric bypass are at an increased risk of perioperative morbidity and mortality following laparoscopic bariatric surgery. Additional studies are needed to determine the association of age with long-term weight loss and cardiometabolic comorbidity resolution following bariatric surgery in order to determine if the increased perioperative risk is offset by improved long-term outcomes in older patients undergoing bariatric surgery.”  **Reviewer’s comments:**  This study shows that older age is significantly associated with increased risk of pulmonary, wound, septic, and renal events, related mortality, and composite morbidity and mortality outcome, but not clotting events, or unplanned reintervention.  This study has clearly met 7/9 (78%) criteria in the critical appraisal tool. |
| **Authors:**  Hajer, et al. [148]  **Year published:** 2018  **Study design:**  Cohort study  **Country:**  Germany | **Sample size:**  N=21,571  **Inclusion criteria:**   - Patients who underwent a primary LSG between January 2005 and December 2016   **Exclusion criteria:**   - Patients who underwent other types of bariatric surgery   **Setting and population:**   - Patient records identified from German Bariatric Surgery Registry | - Age | 1. Postoperative complications group and separately (general: cardiac, pulmonary, renal, UTI, fever, thrombosis; special: blood transfusion, gastroscopy, bleeding and reoperation, staple line leakage, stenosis, ileus, sepsis, abscess, peritonitis, wound infection, burst abdomen) 2. 30-day mortality | **Statistical analysis:**   - Descriptive statistics - “Unadjusted analyses were exploratorily carried out to compare age groups (< 60 years/≥ 60 years). Chi-square tests and robust t tests were performed for categorical and continuous data, respectively.”   **Results/effect estimates:**  1. Postoperative complications (%)  Total general complications   - Age ≥60y: 7.84% (n = 139) - Age <60y: 4.97% (n = 985) - P <0.001   Fever   - Age ≥60y: 0.79% - Age <60y: 1.17% - P = 0.147   Total special complications   - Age ≥60y: 4.51% (n = 828) - Age <60y: 4.18% (n = 908) - P = 0.504   Bleeding and reoperation   - Age ≥60y: 1.75% - Age <60y: 1.31% - P = 0.127   Staple line leakage   - Age ≥60y: 0.96% - Age <60y: 1.29% - P = 0.229   2. Mortality   - Age ≥60y: 0.23% - Age <60y: 0.22% - P = 0.997 | **Author’s conclusion:**  “LSG is a low-risk operation and safe surgical method with acceptable, not elevated perioperative morbidity and mortality rates in patients ≥ 60 years of age.”  **Reviewer’s comments:**  This study shows that older age is significantly associated with increased risk of total general complications, but not fever, total special complications, bleeding and reoperation, staple line leakage, or mortality.  This study has clearly met 5/9 (56%) criteria in the critical appraisal tool. |
| **Authors:**  Gerber, et al. [149]  **Year published:** 2018  **Study design:**  Cohort study  **Country:**  Sweden | **Sample size:**  N=47,660  **Inclusion criteria:**   - Patients who underwent primary GB between May 2007 and October 2016   **Exclusion criteria:**   - Nil   **Setting and population:**   - Patient records identified from the SOReg database | - Age | 1. 30-day postoperative complications (any; surgical: anastomotic leak, bleeding, deep infection/abscess, minor wound complications; medical: thromboembolic event, cardiovascular, pulmonary) 2. 30-day mortality | **Statistical analysis:**   - Descriptive statistics - “The co-variates included in the multivariate analysis were chosen according to stepwise logistic modelling where a P value <0.15 was considered relevant. A logistic regression model was used to estimate odds ratios for various postoperative complications in relation to age. Exposure was defined as age, and the outcome was complications and mortality within 30 days after surgery. The adjustment variables were BMI, sex, and presence of diabetes.”   **Results/effect estimates:**  1. Postoperative complications  Any complications   - Age ≥70y: 3.3%, OR = 0.33 (95% CI: 0.04-2.45) - Age 65-69y: 10.9%, OR = 1.29 (95% CI: 0.87-1.69) - Age 60-64y: 10.2%, OR = 1.16 (95% CI: 0.99-1.36) - Age ≥60y: 10.2%, OR = 1.16 (95% CI: 1.00-1.34) - Age 55-59y: 10.0%, OR = 1.17 (95% CI: 1.05-1.31) - Age 50-54y: 9.8%, OR = 1.17 (95% CI: 1.06-1.29) - Age 45-49y: 8.4%, OR = 0.98 (95% CI: 0.89-1.07) - Age 40-44y: 8.1%, OR = 0.96 (95% CI: 0.88-1.05) - Age 35-39y: 7.9%, OR = 0.94 (95% CI: 0.86-1.04) - Age 30-34y: 8.0%, OR = 0.96 (95% CI: 0.87-1.07) - Age 25-29y: 7.7%, OR = 0.93 (95% CI: 0.83-1.05) - Age 20-24y: 7.2%, OR = 0.86 (95% CI: 0.75-0.99) - Age <20y: OR = 6.4%, 0.77 (95% CI: 0.57-1.06)   Anastomotic leak   - Age ≥70y: N/A - Age 65-69y: 2.7%, OR = 2.09 (95% CI: 1.10-4.00) - Age 60-64y: 1.4%, OR = 1.10 (95% CI: 0.72-1.67) - Age ≥60y: 1.6%, OR = 1.27 (95% CI: 0.89-1.82) - Age 55-59y: 1.9%, OR = 1.72 (95% CI: 1.34-2.22) - Age 50-54y: 1.5%, OR = 1.36 (95% CI: 1.07-1.73) - Age 45-49y: 1.1%, OR = 0.98 (95% CI: 0.77-1.25) - Age 40-44y: 0.9%, OR = 0.79 (95% CI: 0.61-1.01) - Age 35-39y: 0.9%, OR = 0.88 (95% CI: 0.68-1.14) - Age 30-34y: 1.0%, OR = 0.95 (95% CI: 0.72-1.27) - Age 25-29y: 0.7%, OR = 0.63 (95% CI: 0.43-0.91) - Age 20-24y: 0.8%, OR = 0.83 (95% CI: 0.56-1.23) - Age <20y: 0.4%, OR = 0.41 (95% CI: 0.13-1.29)   Postoperative bleeding   - Age ≥70y: 3.3%, OR = 1.43 (95% CI: 0.19-10.56) - Age 65-69y: 1.1%, OR = 0.45 (95% CI: 0.17-1.21) - Age 60-64y: 2.2%, OR = 0.99 (95% CI: 0.71-1.38) - Age ≥60y: 2.0%, OR = 0.89 (95% CI: 0.65-1.22) - Age 55-59y: 2.6%, OR = 1.26 (95% CI: 1.02-1.56) - Age 50-54y: 2.3%, OR = 1.14 (95% CI: 0.94-1.38) - Age 45-49y: 2.5%, OR = 1.24 (95% CI: 1.05-1.47) - Age 40-44y: 2.0%, OR = 1.01 (95% CI: 0.85-1.20) - Age 35-39y: 1.9%, OR = 0.97 (95% CI: 0.81-1.17) - Age 30-34y: 1.6%, OR = 0.84 (95% CI: 0.67-1.05) - Age 25-29y: 1.6%, OR = 0.85 (95% CI: 0.66-1.09) - Age 20-24y: 1.2%, OR = 0.66 (95% CI: 0.47-0.91) - Age <20y: 0.6%, OR = 0.30 (95% CI: 0.11-0.82)   Minor wound complications   - Age ≥70y: N/A - Age 65-69y: 1.6%, OR = 1.45 (95% CI: 0.64-3.30) - Age 60-64y: 2.2%, OR = 2.14 (95% CI: 1.52-3.02) - Age ≥60y: 2.1%, OR = 2.02 (95% CI: 1.46-2.79) - Age 55-59y: 1.2%, OR = 1.18 (95% CI: 0.87-1.60) - Age 50-54y: 1.2%, OR = 1.23 (95% CI: 0.94-1.61) - Age 45-49y: 1.0%, OR = 0.98 (95% CI: 0.76-1.27) - Age 40-44y: 0.9%, OR = 0.95 (95% CI: 0.74-1.22) - Age 35-39y: 1.0%, OR = 1.03 (95% CI: 0.80-1.33) - Age 30-34y: 0.8%, OR = 0.73 (95% CI: 0.53-1.01) - Age 25-29y: 1.0%, OR = 1.07 (95% CI: 0.78-1.46) - Age 20-24y: 0.3%, OR = 0.32 (95% CI: 0.17-0.58) - Age <20y: 0.6%, OR = 0.57 (95% CI: 0.21-1.53)   Any medical complications   - Age ≥70y: N/A - Age 65-69y: 3.5%, OR = 3.43 (95% CI: 1.94-6.08) - Age 60-64y: 1.5%, OR = 1.43 (95% CI: 0.95-2.16) - Age ≥60y: 1.8%, OR = 1.82 (95% CI: 1.29-2.58) - Age 55-59y: 1.2%, OR = 1.22 (95% CI: 0.89-1.66) - Age 50-54y: 1.1%, OR = 1.20 (95% CI: 0.91-1.59) - Age 45-49y: 0.9%, OR = 0.99 (95% CI: 0.76-1.29) - Age 40-44y: 1.0%, OR = 1.16 (95% CI: 0.91-1.49) - Age 35-39y: 0.7%, OR = 0.80 (95% CI: 0.59-1.08) - Age 30-34y: 0.7%, OR = 0.75 (95% CI: 0.53-1.06) - Age 25-29y: 0.6%, OR = 0.68 (95% CI: 0.45-1.01) - Age 20-24y: 0.6%, OR = 0.73 (95% CI: 0.46-1.14) - Age <20y: 0.6%, OR = 0.69 (95% CI: 0.26-1.84)   2. Mortality (%)   - Age ≥70y: 0% - Age 65-69y: 2.7% - Age 60-64y: 1.2% - Age ≥60y: 1.4% - Age 55-59y: 0% - Age 50-54y: 0.6% - Age 45-49y: 0.4% - Age 40-44y: 0.1% - Age 35-39y: 0.4% - Age 30-34y: 0.4% - Age 25-29y: 0% - Age 20-24y: 0% - Age <20y: 0% | **Author’s conclusion:**  “In this large data set, rates of complications and mortality after 30 days were low. For many complications, an increased risk was encountered in patients aged ≥50 years. However, rates of complications and mortality were still acceptably low in these age groups. Taking the expected benefits in terms of weight loss and improvements of co-morbidities into consideration, our findings suggest that patients of older age should be considered for surgery after thorough individual risk assessment rather than denied bariatric surgery based solely on a predefined chronologic age limit.”  **Reviewer’s comments:**  This study shows that age groups 50-54y, 55-59y, and ≥60y are significantly associated with increased risk of any complication, while age groups 50-54y, 55-59y, and 65-69y are significantly associated with increased risk of anastomotic leak. Age groups 45-59y and 55-59y are significantly associated with increased risk of postoperative bleeding, while age groups 20-24y and <20y are significantly associated with decreased risk. Age groups ≥60y and 60-64y are significantly associated with increased risk of minor wound complications, while age group 20-24y is significantly associated with decreased risk. Age groups ≥60y and 65-69y are significantly associated with increased risk of any medical complications. Older age is not associated with increased risk or mortality.  This study has clearly met 8/10 (80%) criteria in the critical appraisal tool. |
| **Authors:**  Dreber, et al. [150]  **Year published:** 2018  **Study design:**  Cohort study  **Country:**  Sweden | **Sample size:**  N=2,579  **Inclusion criteria:**   - Young adult (18-25y) patients who underwent primary RYGB in Sweden from the initiation of SOReg (May 2, 2007) to December 30, 2013 - Older RYGB patients (≥26y) were frequency matched for BMI, sex, and year of surgery   **Exclusion criteria:**   - Nil   **Setting and population:**   - Patient records identified from the SOReg database | - Age | 1. Adverse events (leak, bleeding, abscess, wound complications, port-related complication, cardiovascular complication, VTE, UTI (all evaluated only at 6 wks), ileus, anastomotic stricture, stomal ulcer, perforation, hernia, anaemia/malnutrition requiring intervention, other non-specified RYGB-related adverse event e.g. biliary stones) | **Statistical analysis:**   - Descriptive statistics - “The odds ratios of adverse events in young versus older adults were calculated by logistic regression, and the relative risk for loss to follow-up was analyzed by Poisson regression.” The adjustment variables for adverse events were baseline BMI, sex, year of surgery, co-morbidity, surgical access, duration of surgery, and surgical volume.   **Results/effect estimates:**  1. Any adverse event (%)  0-6 wks   - Age 18-25y: 6.9% - Age ≥26y: 8.6% - P = 0.23   6 wks-1 y   - Age 18-25y: 7.6% - Age ≥26y: 5.6% - P <0.001   1-2 y   - Age 18-25y: 10.4% - Age ≥26y: 7.6% - P <0.001   2-5 y   - Age 18-25y: 20.3% - Age ≥26y: 12.7% - P <0.001 | **Author’s conclusion:**  “While young adults displayed at least equal weight loss as older adults, rates of adverse events were approximately doubled, and loss to follow-up rates were higher. Future studies on the significance of and the etiology behind the higher incidence of serious adverse events are needed. Intensified clinical contact post Roux-en-Y gastric bypass should have the potential to further improve outcomes in young adults.”  **Reviewer’s comments:**  This study shows that older age is significantly associated with increased risk of any adverse event between 6 weeks and 1 year after surgery, but not within 6 weeks.  This study has clearly met 7/10 (70%) criteria in the critical appraisal tool. |
| **Authors:**  Cesana, et al. [151]  **Year published:** 2018  **Study design:**  Cohort study  **Country:**  Italy | **Sample size:**  N=1,738  **Inclusion criteria:**   - Patients who underwent LSG from January 2008 to October 2016   **Exclusion criteria:**   - Patients who underwent LSG after a previous different bariatric intervention   **Setting and population:**   - Patient records identified from a prospectively maintained database including obese patients enrolled for LSG at San Giuseppe Hospital, Milan (2008-2010), and at San Marco Hospital, Zingonia, Bergamo (2010-2016) | - Age - Sex | 1. Proximal leakage following LSG | **Statistical analysis:**   - Descriptive statistics - “The analysis regarding the main aim was performed using the logistic regression model. Bivariate odds ratios (ORs) and 95% confidence intervals (95% CIs) were estimated for each potential predictor present in the database. The multivariate logistic regression model was performed using both the stepwise selection procedure considering all the variables collected in the database and the selection based on the significant association (p ≤ 0.05) identified from the bivariate logistic analysis.”   **Results/effect estimates:**  1. Proximal leakage (%)  Age (y)   - ≤33: 2.9% (13/454), OR = 1.15 (95% CI: 0.60-2.22, p = 0.669) - 34-41: 3.3% (14/424), OR = 1.41 (95% CI: 0.74-2.68, p = 0.290) - 42-49: 2.6% (12/460), OR = 1.01 (95% CI: 0.52-1.97, p = 0.975) - >49: 1.5% (6/400), OR = 0.51 (95% CI: 0.21-1.21, p = 0.125)   Sex (male)   - 3.0% (13/428), OR = 1.25 (95% CI: 0.65-2.41, p = 0.502) | **Author’s conclusion:**  “In this large consecutive cohort study of LSG, proximal staple line reinforcement (buttress material or suture) reduced the risk of a leak. The risk of a proximal leak was much higher in the surgeons first 100 cases, which has implications for training and supervision during this ‘learning curve’ period”.  **Reviewer’s comments:**  This study shows that older age, and male sex are not associated with increased risk of proximal leakage.  This study has clearly met 8/10 (80%) criteria in the critical appraisal tool. |
| **Authors:**  Altieri, et al. [152]  **Year published:** 2018  **Study design:**  Cohort study  **Country:**  United States | **Sample size:**  N=36,947  **Inclusion criteria:**   - All patients who underwent RYGB, SG, and LAGB between 2004 and 2010   **Exclusion criteria:**   - Patients with a missing identifier number - Patients aged <18y - Patients with missing data - Patients who underwent cholecystectomy at the time of the initial procedure or before that since 2000 - Patients who were lost to follow-up   **Setting and population:**   - Patient records identified from the Statewide Planning and Research Collaborative System administrative longitudinal database (New York State) | - Age - Race - Sex | 1. Subsequent cholecystectomy | **Statistical analysis:**   - Descriptive statistics - “Any of patients’ characteristics, co-morbidities, or complications at the time of bariatric surgery that were associated with having subsequent cholecystectomies were further considered in a multivariable Cox proportional hazard model to examine its independent association of having follow-up cholecystectomy. Hazard ratios (HR) of all possible predictors and their corresponding 95% CIs were reported.”   **Results/effect estimates:**  1. Subsequent cholecystectomy (%)  Age (y)   - P <0.0001 - 36-50 vs 18-35: HR = 0.77 (95% CI: 0.71-0.84) - ≥55 vs 18-35: HR = 0.59 (95% CI: 0.53-0.66)   Race   - P <0.0001 - Black vs White: HR = 0.61 (95% CI: 0.52-0.69) - Hispanic vs White: HR = 1.11 (95% CI: 0.99-1.26) - Other vs White: HR = 0.98 (95% CI: 0.88-1.09)   Sex   - P <0.0001 - Female vs Male: HR = 1.26 (95% CI: 1.15-1.39) | **Author’s conclusion:**  “The rate of cholecystectomy after LAGB, RYGB, and SG  was 6.5%, 9.7% and 10.1%, respectively. After accounting for other variables, patients after either LAGB or RYGB were less likely to undergo a subsequent cholecystectomy. In addition, CBD injury had a higher rate of 0.12% during subsequent cholecystectomy. Patients should be counselled preoperatively about this risk. The risks and benefits of ursodiol or prophylactic cholecystectomy should also be considered.”  **Reviewer’s comments:**  This study shows that younger age, white race, and female sex are significantly associated with increased risk of requiring a subsequent cholecystectomy.  This study has clearly met 8/10 (80%) criteria in the critical appraisal tool. |
| **Authors:**  Alizadeh, et al. [153]  **Year published:** 2018  **Study design:**  Cohort study  **Country:**  United States | **Sample size:**  N=133,478  **Inclusion criteria:**   - Patients who underwent LSG or LRYGB in 2015   **Exclusion criteria:**   - Patients who underwent emergent, revisional, or converted surgeries   **Setting and population:**   - Patient records identified from the 2015 MBSAQIP database | - Age - Sex - Race | - Gastrointestinal leak after LSG or LRYGB (30-day leak outcomes, drain present for >30 days, OS-SSI, leak-related 30-day readmission/reoperation/intervention) | **Statistical analysis:**   - Descriptive statistics - “A multivariate logistic regression model was used to analyze the risk factors for gastrointestinal leak, as well as the independent association between various intraoperative and postoperative interventions with the development of gastrointestinal leak.”   **Results/effect estimates:**  1. Age   - Cases With gastrointestinal leak: mean 45(±12)y - Controls Without gastrointestinal leak: mean 44(±12)y - P = 0.67 - AOR = 1.00 (95% CI: 0.99-1.01, p = 0.85)   2. Sex (male)   - Cases With gastrointestinal leak: 23.5% (220/938) - Controls Without gastrointestinal leak: 21% (21,871/132,540) - P = 0.24 - AOR = 1.12 (95% CI: 0.95-1.32, p = 0.16)   3. Race  White (Ref)   - Cases With gastrointestinal leak: 84.4% (575/938) - Controls Without gastrointestinal leak: 80.4% (98,919/132,540) - P = 0.31   African American   - Cases With gastrointestinal leak: 14.7% (138/938) - Controls Without gastrointestinal leak: 18.4% (22,623/132,540) - P = 0.12 - AOR = 0.22 (95% CI: 0.03-1.59, p = 0.13)   Asian   - Cases With gastrointestinal leak: 0.1% (1/938) - Controls Without gastrointestinal leak: 0.5% (623/132,540) - P = 0.05 - AOR = 0.14 (95% CI: 0.02-1.04, p = 0.05)   Other ethnicity   - Cases With gastrointestinal leak: 0.1% (1/938) - Controls Without gastrointestinal leak: 0.7% (894/132,540) | **Author’s conclusion:**  “The overall rate of gastrointestinal leak after LSG and LRYGB is low at 0.7%, with sleeve gastrectomy having a significantly lower leak rate compared with gastric bypass. Hypertension, diabetes mellitus, sleep apnea, oxygen dependency, and hypoalbuminemia were found to be independent risk factors for gastrointestinal leak. The use of intraoperative provocative testing and placement of a surgical drain were associated with a higher leak rate, but performance of a postoperative swallow evaluation had no impact on the leak rate.”  **Reviewer’s comments:**  This study shows that Asian ethnicity, but not older age, or male sex, is significantly associated with increased risk of gastrointestinal leak.  This study has clearly met 9/10 (90%) criteria in the critical appraisal tool. |
| **Authors:**  Al-Kurd, et al. [154]  **Year published:** 2018  **Study design:**  Cohort study  **Country:**  Israel | **Sample size:**  N=90  **Inclusion criteria:**   - Study group: All patients aged ≥70y who underwent LSG between January 1, 2012 and December 31, 2017 - Control group: A randomly selected cohort of LSG patients aged 18-50y was matched by gender, BMI, and date of operation (within 2 months’ time), by a ratio of 1:2   **Exclusion criteria:**   - Nil   **Setting and population:**   - Patient records identified from a prospectively maintained bariatric surgery database at the Hadassah-Hebrew University Medical Centre | - Age | 1. Postoperative complications group and separately (anastomotic leak, intra-abdominal bleeding, GI bleeding, PE, pneumonia, new arrhythmia, MI, UTI, re-operation within 1 month, emergency room visit within 1 month, re-hospitalisation within 1 month) | **Statistical analysis:**   - Descriptive statistics - “univariate analysis with t test and chi-square was utilized to compare between the various subgroups”   **Results/effect estimates:**  1. Postoperative complications (%)  Overall   - Age ≥70y: 10.0% (3/30) - Age 18-50y: 5.0% (3/60) - P = 0.38   Intra-abdominal bleeding   - Age ≥70y: 3.3% (1/30) - Age 18-50y: 0% (0/60) - P = 0.16   Pneumonia   - Age ≥70y: 3.3% (1/30) - Age 18-50y: 1.7% (1/60) - P = 0.62   Emergency room visit within 1 month   - Age ≥70y: 16.7% (5/30) - Age 18-50y: 3.3% (2/60) - P = 0.007   Re-hospitalisation within 1 month   - Age ≥70y: 10.0% (3/30) - Age 18-50y: 3.3% (2/60) - P = 0.48 | **Author’s conclusion:**  “In a carefully selected group of severely obese patients ≥ 70 years old, LSG may be safe, with acceptable postoperative complication rates, weight loss results, and improvement in comorbidities.”  **Reviewer’s comments:**  This study shows that older age is significantly associated with increased risk of emergency room visit within 1 month, but not overall postoperative complications, intra-abdominal bleeding, pneumonia, or re-hospitalisation within 1 month.  This study has clearly met 7/10 (70%) criteria in the critical appraisal tool. |
| **Authors:**  Spivak, et al. [155]  **Year published:** 2017  **Study design:**  Cohort study  **Country:**  Israel | **Sample size:**  N=394  **Inclusion criteria:**   - Patients who underwent primary (non-revisional) LSG performed by a single surgeon from January 1, 2014 to December 31, 2015 at a single institution   **Exclusion criteria:**   - Nil   **Setting and population:**   - Patient records identified from a single institution in Israel | - Age - Sex | 1. Postoperative haemorrhage within 90 days | **Statistical analysis:**   - Descriptive statistics - Univariate analyses were done to determine the relationship of bleeders and non-bleeders with age, BMI, and comorbidities - “The variables included in the logistic regression were chosen according to p values in univariate analyses, where p < 0.05 was considered statistically significant. The logistic regression model was used to estimate predictors for postoperative haemorrhage.”   **Results/effect estimates:**  1. Postoperative haemorrhage (%)  Female (%)   - Cases All postoperative haemorrhage: 65.8% (25/38) - Controls Non-bleeders: 67.3% (210/312) - P >0.05   Mean age (y)   - Cases All postoperative haemorrhage: 43.5(±13.1) - Controls Non-bleeders: 41.7(±11.5) - P = 0.05 | **Author’s conclusion:**  “In this study, acute and subclinical POH were primarily linked to T2D and not to surgical techniques. Special consideration is recommended for patients with T2D undergoing SG.”  **Reviewer’s comments:**  This study shows that older age, but not female sex, is significantly associated with increased risk of postoperative haemorrhage.  This study has clearly met 9/10 (90%) criteria in the critical appraisal tool. |
| **Authors:**  Navarrete, et al. [156]  **Year published:** 2017  **Study design:**  Cohort study  **Country:**  Spain | **Sample size:**  N=206  **Inclusion criteria:**   - All patients aged ≥60y who underwent LSG at Hospital Clinic of Barcelona between January 2006 and December 2012 - Patients aged <60y who also underwent LSG were selected for comparison, matched by sex, BMI, ASA classification group, and baseline comorbidities   **Exclusion criteria:**   - Nil   **Setting and population:**   - Patient records identified from a prospectively maintained database of all bariatric surgeries performed at Hospital Clinic of Barcelona | - Age | 1. 30-day postoperative complications group and separately (leak, haemorrhage, fluid collection, pulmonary thromboembolism, cardiac failure, others) 2. 30-day mortality | **Statistical analysis:**   - Descriptive statistics - “Parameters were analyzed and compared using … the χ2 test (or Fisher exact) test for categorical variables.”   **Results/effect estimates:**  1. Postoperative complications (%)  Overall   - Age ≥60y: 9.7% (10/103) - Age <60y: 15.5% (16/103) - P = 0.2   Leak   - Age ≥60y: 2.9% (3/103) - Age <60y: 1.9% (2/103) - P = 0.3   Haemorrhage   - Age ≥60y: 1.9% (2/103) - Age <60y: 8.7% (9/103) - P = 0.3   Fluid collection   - Age ≥60y: 1.9% (2/103) - Age <60y: 0% (0/103) - P = 0.3   Others   - Age ≥60y: 1.0% (1/103) - Age <60y: 2.9% (3/103)   2. Mortality (%)   - Age ≥60y: 0% (0/103) - Age <60y: 0% (0/103) | **Author’s conclusion:**  “SG is a safe and feasible procedure in the elderly, with  results comparable to those in the standard bariatric population. Although weight loss may be not as great as in younger patients, it still is acceptable and successful. Remission of co-morbidities displayed adequate results, similar to that of the younger group in the long-term follow-up. Therefore, age alone should not be an absolute contraindication for SG, and SG should be considered as an option in the elderly obese population.”  **Reviewer’s comments:**  This study shows that older age is not associated with increased risk of postoperative complications, or mortality.  This study has clearly met 7/10 (70%) criteria in the critical appraisal tool. |
| **Authors:**  Major, et al. [157]  **Year published:** 2017  **Study design:**  Cohort study  **Country:**  Poland | **Sample size:**  N=408  **Inclusion criteria:**   - Patients aged 18-65y who underwent LSG or LRYGB, meeting the criteria for morbid obesity surgical treatment i.e. BMI ≥35 kg/m^2^ with obesity comorbidities, or BMI ≥40 kg/m^2^   **Exclusion criteria:**   - Patients with a lack of necessary data - Patients who underwent previous operations for morbid obesity   **Setting and population:**   - Patient records identified from a prospectively collected data of patients operated for morbid obesity at the 2^nd^ Department of Surgery, Jagiellonian University Medical College, Cracow, Poland | - Sex - Age | 1. 30-day postoperative complications (PE, rhabdomyolysis, peritonitis, strangulated operation site hernia, jejunojejunal anastomosis leak, cardiorespiratory failure, pneumonia, ARDS, gastrointestinal leakage, bleeding from suture line, Petersen’s space hernia, pneumonia, fever of unknown origin, delayed gastric emptying, dehydration, prolonged drainage, biochemical rhabdomyolysis) | **Statistical analysis:**   - Descriptive statistics - “Univariate and multivariate logistic regression models included quantitative and qualitative data to assess the influence of selected parameters on outcomes and elicit independent risk factors influencing odds ratio of perioperative complications.”   **Results/effect estimates:**  1. Postoperative complications (%)   - Sex (male vs female): OR = 0.57 (95% CI: 0.26-1.26, p = 0.164) - Age (1y): OR = 1.01 (95% CI: 0.97-1.04, p = 0.644) | **Author’s conclusion:**  “Important risk factors for perioperative complications are related both to patient's characteristics and the procedure itself. Longer operative time and the increase in the number of stapler firings needed should alert a surgeon to an increased risk of perioperative complications following the laparoscopic sleeve gastrectomy. For patients submitted to LRYGB, particular attention should be paid to patients with higher BMI.”  **Reviewer’s comments:**  This study shows that male sex and older age are not associated with increased risk of postoperative complications.  This study has clearly met 8/10 (80%) criteria in the critical appraisal tool. |
| **Authors:**  Halawani, et al. [158]  **Year published:** 2017  **Study design:**  Cohort study  **Country:**  United States | **Sample size:**  N=662  **Inclusion criteria:**   - Patients who underwent laparoscopic or robotic BPD-DS between January 1, 2006 and December 31, 2016   **Exclusion criteria:**   - Patients who underwent revisions or prior weight loss procedures   **Setting and population:**   - Patient records identified from prospectively collected data at a nonprofit regional referral centre and teaching hospital in Pennsylvania | - Age | - VTE (DVT or PE) within 90 days of BPD-DS surgery | **Statistical analysis:**   - Descriptive statistics - “Analysis of variance and χ2 analyses were used to identify continuous and categoric variables, respectively, that were significantly associated with VTE occurrence. Those variables that were associated with VTE occurrence were included in a standard binomial logistic regression model.”   **Results/effect estimates:**  1. Age (y)   - Cases VTE: mean 45.9(±9.5)y - Controls No VTE: mean 44.6(±10.4)y - P = 0.616 | **Author’s conclusion:**  “BPD-DS is a complex bariatric procedure performed on theoretically higher risk patients due to increased weight and associated co-morbidities. With proper preoperative evaluation and an aggressive VTE prophylaxis protocol, the risk of VTE is comparable to that of other bariatric procedures. LOS is statistically associated with the development of VTE. Every effort should be taken to reduce VTE risk factors, including LOS.”  **Reviewer’s comments:**  This study shows that older age is not associated with increased risk of VTE.  This study has clearly met 10/10 (100%) criteria in the critical appraisal tool. |
| **Notes:**  ^a^ Results were extracted separately for RYGB and SG as published, rather than all procedures combined.  ^b^ Postoperative complications were also reported according to their Clavien-Dindo classification, however, these results were not extracted due to the large number of comparisons made in the analysis.  ^c^ Results were extracted separately for RYGB and LAGB, rather than all procedures combined.  ^d^ Results were extracted as published, and may contain errors.  **Abbreviations:**  AKI = Acute Kidney Injury; AOR = Adjusted Odds Ratio; AQ = Age-Qualified; ARF = Acute Renal Failure; ASA = American Society of Anaesthesiologists; BMI = Body Mass Index; BPD-DS = Biliopancreatic Diversion With Duodenal Switch; CDI = Clostridium Difficile Infection; CI = Confidence Interval; CVA = Cerebrovascular Accident; COE = Centre of Excellence; CVD = Cardiovascular Disease; DQ = Disability-Qualified; DVT = Deep Vein Thrombosis; HR = Hazards Ratio; GB = Gastric Bypass; GIT = Gastrointestinal Tract; GJ = Gastrojejunostomy; GORD = Gastroesophageal Reflux Disease; ICD = International Classification of Disease; ICU = Intensive Care Unit; IMD = Index of Multiple Deprivation; MBSAQIP = Metabolic and Bariatric Surgery Accreditation and Quality Improvement; MBSC = Michigan Bariatric Surgery Collaborative; LAGB = Laparoscopic Adjustable Gastric Banding; LRYGB = Laparoscopic Roux-en-Y Gastric Bypass; LSG = Laparoscopic Sleeve Gastrectomy; MI = Myocardial Infarction; NHS = National Health Services; NM = Non-Medicare; OAGB = One Anastomosis Gastric Bypass; OR = Odds Ratio; OS-SSI = Organ Space Surgical Site Infection; RD = Risk Difference; RYGB = Roux-en-Y Gastric Bypass; PE = Pulmonary Embolism; PMVT = Portomesenteric Vein Thrombosis; PONV = Postoperative Nausea and Vomiting; PPD = Proximal Pouch Distension; PUF = Participant Use File; SAE = Serious Adverse Event; SDH = Social Determinant of Health; SG = Sleeve Gastrectomy; SGJB = Sleeve Gastrectomy with Jejunal Bypass; SOReg = Scandinavian Obesity Surgery Registry; SSI = Surgical Site Infection; T2DM = Type 2 Diabetes Mellitus; UTI = Urinary Tract Infection; VSG = Vertical Sleeve Gastrectomy; VTE = Venous Thromboembolism | | | | | |

# Supplementary Table S5: Risk of bias assessment of studies reviewed

| **Cohort** | **1.     Were the two groups similar and recruited from the same population?** | **2.     Were the exposures measured similarly to assign people to both exposed and unexposed groups?** | **3.  Was the exposure measured in a valid and reliable way?** | **4.Were confounding factors identified?** | **5.     Were strategies to deal with confounding factors stated?** | **6.     Were the groups/participants free of the outcome at the start of the study (or at the moment of exposure)?** | **7.     Were the outcomes measured in a valid and reliable way?** | **8.     Was the follow up time reported and sufficient to be long enough for outcomes to occur?** | **9.     Was follow up complete, and if not, were the reasons to loss to follow up described and explored?** | **10.  Were strategies to address incomplete follow up utilized?** | **11.  Was appropriate statistical analysis used?** | **Overall Quality** | **Unclear** | **Proportion** |
| --- | --- | --- | --- | --- | --- | --- | --- | --- | --- | --- | --- | --- | --- | --- |
| Vallois et al. (2022) [88] | Unclear | Yes | Yes | Yes | Yes | Not applicable | Yes | Yes | No | No | Yes | 7/10 | 1 | 70% |
| Singhal et al. (2022) [89] | Unclear | Yes | Yes | No | No | Not applicable | Yes | Yes | Yes | Not applicable | Yes | 6/9 | 1 | 67% |
| Khalid et al. (2022) [90] | Unclear | Yes | Yes | Yes | Yes | Not applicable | Yes | Yes | Unclear | Not applicable | Yes | 7/9 | 2 | 78% |
| Iranmanesh et al. (2022) [91] | Unclear | Yes | Yes | No | No | Not applicable | Yes | Yes | Unclear | Not applicable | Yes | 5/9 | 2 | 56% |
| Nafiu et al. (2021) [92] | Unclear | Yes | Yes | Yes | Yes | Not applicable | Yes | Yes | Unclear | Not applicable | Yes | 7/9 | 2 | 78% |
| Mousapour et al. (2021) [93] | Unclear | Yes | Yes | Yes | Yes | Not applicable | Yes | Yes | Yes | Not applicable | Yes | 8/9 | 1 | 89% |
| Hui et al. (2021) [94] | Unclear | Yes | Yes | Yes | Yes | Not applicable | Yes | Yes | Yes | Not applicable | Yes | 8/9 | 1 | 89% |
| Di Palma et al. (2021) [95] | Unclear | Yes | Yes | Yes | Yes | Not applicable | Yes | Yes | Yes | Not applicable | Yes | 8/9 | 1 | 89% |
| Bal et al. (2021) [96] | Unclear | Yes | Yes | No | No | Not applicable | Yes | Yes | Unclear | Not applicable | Yes | 5/9 | 2 | 56% |
| Athanasiadis et al. (2021) [97] | Unclear | Yes | Yes | Yes | Yes | Not applicable | Yes | Yes | Unclear | Not applicable | Yes | 7/9 | 2 | 78% |
| Aryannezhad et al. (2021) [98] | Yes | Yes | Yes | Yes | Yes | Not applicable | Yes | Yes | Unclear | Not applicable | Yes | 8/9 | 1 | 89% |
| Welsh et al. (2020) [99] | Unclear | Yes | Yes | Yes | Yes | Not applicable | Yes | Yes | Unclear | Not applicable | Yes | 7/9 | 2 | 78% |
| Turchi et al. (2020) [100] | Yes | Yes | Yes | Yes | Yes | Not applicable | Yes | Yes | Unclear | Not applicable | Yes | 8/9 | 1 | 89% |
| Sundaresan et al. (2020) [101] | Unclear | Yes | Yes | Yes | Yes | Not applicable | Yes | Yes | Unclear | Not applicable | Yes | 7/9 | 2 | 78% |
| Molero et al. (2020) [102] | Yes | Yes | Yes | Yes | Yes | Not applicable | Yes | Yes | Unclear | Not applicable | Yes | 8/9 | 1 | 89% |
| Mocanu et al. (2020) [103] | Unclear | Yes | Yes | Yes | Yes | Not applicable | Yes | Yes | Unclear | Not applicable | Yes | 7/9 | 2 | 78% |
| Maloney et al. (2020) [104] | Unclear | Yes | Yes | Yes | Yes | Not applicable | Yes | Yes | Unclear | Not applicable | Yes | 7/9 | 2 | 78% |
| Hoffman et al. (2020) [105] | Unclear | Yes | Yes | Yes | Yes | Not applicable | Yes | Yes | Unclear | Not applicable | Yes | 7/9 | 2 | 78% |
| Gambhir et al. (2020) [107] | Unclear | Yes | Yes | Yes | Yes | Not applicable | Yes | Yes | Unclear | Not applicable | Yes | 7/9 | 2 | 78% |
| Dugan et al. (2020) [108] | Unclear | Yes | Yes | No | No | Not applicable | Yes | Yes | Unclear | Not applicable | Yes | 5/9 | 2 | 56% |
| Amirian et al. (2020) [109] | Unclear | Yes | Yes | Yes | Yes | Not applicable | Yes | Yes | Unclear | Not applicable | Yes | 7/9 | 2 | 78% |
| Alimogullari and Bulus (2020) [110] | Unclear | Yes | Yes | Yes | Yes | Not applicable | Yes | Yes | Yes | Not applicable | Yes | 8/9 | 1 | 89% |
| Wood et al. (2019) [111] | Unclear | Yes | Yes | Yes | Yes | Not applicable | Yes | Yes | Unclear | Not applicable | Yes | 7/9 | 2 | 78% |
| Walker et al. (2019) [112] | Unclear | Yes | Yes | No | No | Not applicable | Yes | Yes | Unclear | Not applicable | Yes | 5/9 | 2 | 56% |
| Vidarsson et al. (2019) [113] | Unclear | Yes | Yes | Yes | Yes | Not applicable | Yes | Yes | No | No | Yes | 6/9 | 1 | 67% |
| Sun et al. (2019) [114] | Yes | Yes | Yes | Yes | Yes | Not applicable | Yes | Yes | Unclear | Not applicable | Yes | 8/9 | 1 | 89% |
| Stenberg et al. (2019) [115] | Unclear | No | Yes | Yes | Yes | Not applicable | Yes | Yes | Unclear | Not applicable | Yes | 6/9 | 2 | 67% |
| Smith et al. (2019) [116] | Unclear | Yes | Yes | Yes | Yes | Not applicable | Yes | Yes | Unclear | Not applicable | Yes | 7/9 | 2 | 78% |
| Sheka et al. (2019) [117] | Unclear | Yes | Yes | Yes | Yes | Not applicable | Yes | Yes | Unclear | Not applicable | Yes | 7/9 | 2 | 78% |
| Ozdas and Bozkurt (2019) [118] | Yes | Yes | Yes | No | No | Not applicable | Yes | Yes | Yes | Yes | Yes | 8/10 | 0 | 80% |
| Nickel et al. (2019) [119] | Yes | Yes | Yes | Yes | Yes | Not applicable | Yes | Yes | Unclear | Not applicable | Yes | 8/9 | 1 | 89% |
| Nevo et al. (2019) [120] | Yes | Yes | Yes | No | No | Not applicable | Yes | Yes | Yes | Not applicable | Yes | 7/9 | 0 | 78% |
| Mocanu et al. (2019) [122] | Unclear | Yes | Yes | Yes | Yes | Not applicable | Yes | Yes | Unclear | Not applicable | Yes | 7/9 | 2 | 78% |
| Mocanu et al. (2019) [121] | Unclear | Yes | Yes | Yes | Yes | Not applicable | Yes | Yes | Unclear | Not applicable | Yes | 7/9 | 2 | 78% |
| Martin et al. (2019) [123] | Yes | Yes | Yes | No | No | Not applicable | Yes | Yes | Unclear | Not applicable | Yes | 6/9 | 1 | 67% |
| Ivanics et al. (2019) [124] | Unclear | Yes | Yes | Yes | Yes | Not applicable | Yes | Yes | Yes | Not applicable | Yes | 8/9 | 1 | 89% |
| Guzman et al. (2019) [125] | Yes | Yes | Yes | Yes | Yes | Not applicable | Yes | Yes | Yes | Yes | Yes | 10/10 | 0 | 100% |
| Goldberg et al. (2019) [126] | Unclear | Yes | Yes | Yes | Yes | Not applicable | Yes | Yes | Unclear | Not applicable | Yes | 7/9 | 2 | 78% |
| El Chaar et al. (2019) [127] | Unclear | Yes | Yes | Yes | Yes | Not applicable | Yes | Yes | Unclear | Not applicable | Yes | 7/9 | 2 | 78% |
| Doumouras et al. (2019) [128] | Unclear | Yes | Yes | Yes | Yes | Not applicable | Yes | Yes | Unclear | Not applicable | Yes | 7/9 | 2 | 78% |
| Dang et al. (2019) [129] | Unclear | Yes | Yes | Yes | Yes | Not applicable | Yes | Yes | Unclear | Not applicable | Yes | 7/9 | 2 | 78% |
| Dang et al. (2019) [130] | Unclear | Yes | Yes | Yes | Yes | Not applicable | Yes | Yes | Unclear | Not applicable | Yes | 7/9 | 2 | 78% |
| Clapp et al. (2019) [131] | Unclear | Yes | Yes | Yes | Yes | Not applicable | Yes | Yes | Unclear | Not applicable | Yes | 7/9 | 2 | 78% |
| Bhandari et al. (2019) [132] | Yes | Yes | Yes | No | No | Not applicable | Yes | Yes | No | No | Yes | 6/10 | 0 | 60% |
| Arnold et al. (2019) [133] | Unclear | Yes | Yes | Yes | Yes | Not applicable | Yes | Yes | Unclear | Not applicable | Yes | 7/9 | 2 | 78% |
| Almby and Edholm (2019) [134] | Unclear | Yes | Yes | Yes | Yes | Not applicable | Yes | Yes | Unclear | Not applicable | Yes | 7/9 | 2 | 78% |
| Tang et al. (2018) [135] | Yes | Yes | Yes | Yes | Yes | Not applicable | Yes | Yes | No | No | Yes | 8/10 | 0 | 80% |
| Sun et al. (2018) [136] | Yes | Yes | Yes | Yes | Yes | Not applicable | Yes | Yes | No | No | Yes | 8/10 | 0 | 80% |
| Schurner et al. (2018) [137] | Yes | No | Yes | Yes | Yes | Not applicable | Yes | Yes | Yes | No | Yes | 8/10 | 0 | 80% |
| McCracken et al. (2018) [139] | Yes | Yes | Yes | Yes | Yes | Not applicable | Yes | Yes | Unclear | Not applicable | Yes | 8/9 | 1 | 89% |
| Major et al. (2018) [140] | Unclear | Yes | Yes | Yes | Yes | Not applicable | Yes | Yes | Yes | No | Yes | 8/10 | 1 | 80% |
| Mackay et al. (2018) [141] | Yes | Yes | Yes | Yes | Yes | Not applicable | Yes | Yes | Yes | No | Yes | 9/10 | 0 | 90% |
| Koh et al. (2018) [142] | Unclear | Yes | Yes | Yes | Yes | Not applicable | Yes | Yes | Unclear | Not applicable | Yes | 7/9 | 2 | 78% |
| Kochkodan et al. (2018) [143] | Unclear | Yes | Yes | Yes | Yes | Not applicable | Yes | Yes | No | No | Yes | 7/10 | 1 | 70% |
| Kaplan et al. (2018) [144] | Unclear | Yes | Yes | Yes | No | Not applicable | Yes | Yes | Unclear | Not applicable | Yes | 6/9 | 2 | 67% |
| Hussan et al. (2018) [145] | Unclear | Yes | Yes | Yes | Yes | Not applicable | Yes | Yes | Unclear | Not applicable | Yes | 7/9 | 2 | 78% |
| Hennings et al. (2018) [146] | Unclear | Yes | Yes | Yes | Yes | Not applicable | No | No | Unclear | Not applicable | Yes | 5/9 | 2 | 56% |
| Haskins et al. (2018) [147] | Unclear | Yes | Yes | Yes | Yes | Not applicable | Yes | Yes | Unclear | Not applicable | Yes | 7/9 | 2 | 78% |
| Hajer et al. (2018) [148] | Unclear | Yes | Yes | No | No | Not applicable | Yes | Yes | Unclear | Not applicable | Yes | 5/9 | 2 | 56% |
| Gerber et al. (2018) [149] | Unclear | Yes | Yes | Yes | Yes | Not applicable | Yes | Yes | Yes | No | Yes | 8/10 | 1 | 80% |
| Dreber et al. (2018) [150] | Unclear | Yes | Yes | Yes | Yes | Not applicable | Yes | Yes | No | No | Yes | 7/10 | 1 | 70% |
| Cesana et al. (2018) [151] | No | Yes | Yes | Yes | Yes | Not applicable | Yes | Yes | Unclear | Not applicable | Yes | 7/9 | 1 | 78% |
| Altieri et al. (2018) [152] | Unclear | Yes | Yes | Yes | Yes | Not applicable | Yes | Yes | No | Yes | Yes | 8/10 | 1 | 80% |
| Alizadeh et al. (2018) [153] | Unclear | Yes | Yes | Yes | Yes | Not applicable | Yes | Yes | Unclear | Not applicable | Yes | 7/9 | 2 | 78% |
| Al-Kurd et al. (2018) [154] | Yes | Yes | Yes | No | No | Not applicable | Yes | Yes | Yes | No | Yes | 7/10 | 0 | 70% |
| Spivak et al. (2017) [155] | Yes | Yes | Yes | Yes | Yes | Not applicable | Yes | Yes | Unclear | Not applicable | Yes | 8/9 | 1 | 89% |
| Navarrete et al. (2017) [156] | Yes | Yes | Yes | No | No | Not applicable | Yes | Yes | Yes | No | Yes | 7/10 | 0 | 70% |
| Major et al. (2017) [157] | Yes | Yes | Yes | Yes | Yes | Not applicable | Yes | Yes | Unclear | Not applicable | Yes | 8/9 | 1 | 89% |
| Halawani et al. (2017) [158] | Yes | Yes | Yes | Yes | Yes | Not applicable | Yes | Yes | Unclear | Not applicable | Yes | 8/9 | 1 | 89% |
| **Case-control** | **1. Were the groups comparable other than the presence of disease in cases or the absence of disease in controls?** | **2. Were cases and controls matched appropriately?** | **3. Were the same criteria used for identification of cases and controls?** | **4. Was exposure measured in a standard, valid and reliable way?** | **5. Was exposure measured in the same way for cases and controls?** | **6. Were confounding factors identified?** | **7. Were strategies to deal with confounding factors stated?** | **8. Were outcomes assessed in a standard, valid and reliable way for cases and controls?** | **9. Was the exposure period of interest long enough to be meaningful?** | **10. Was appropriate statistical analysis used?** |  | **Overall Quality** | **Unclear** | **Proportion** |
| Haal et al. (2020) [106] | Unclear | Yes | Yes | Yes | Yes | Yes | Yes | Yes | Yes | Yes |  | 9/10 | 1 | 90% |
| Moon et al. (2018) [138] | Unclear | Yes | Yes | Yes | Yes | Yes | Yes | Yes | Yes | Yes |  | 9/10 | 1 | 90% |

# Supplementary Section S6: Sensitivity analyses

## S6.1: Mortality as a postoperative complication: sensitivity analysis excluding studies with different age groupings

A pooled analysis of five studies [89, 91, 104, 133, 149] demonstrated a significantly increased relative risk of postoperative mortality among older compared to younger patients (RR = 3.33, 95% CI: 2.55-4.34, Figure S6.1). The analysis did not detect any statistically significant evidence of heterogeneity between studies (I² = 0.00%, Q-statistic p-value = 0.4564).

Figure S6.1: Forest plot of relative risk for postoperative mortality associated with older relative to younger age excluding studies with different age groupings


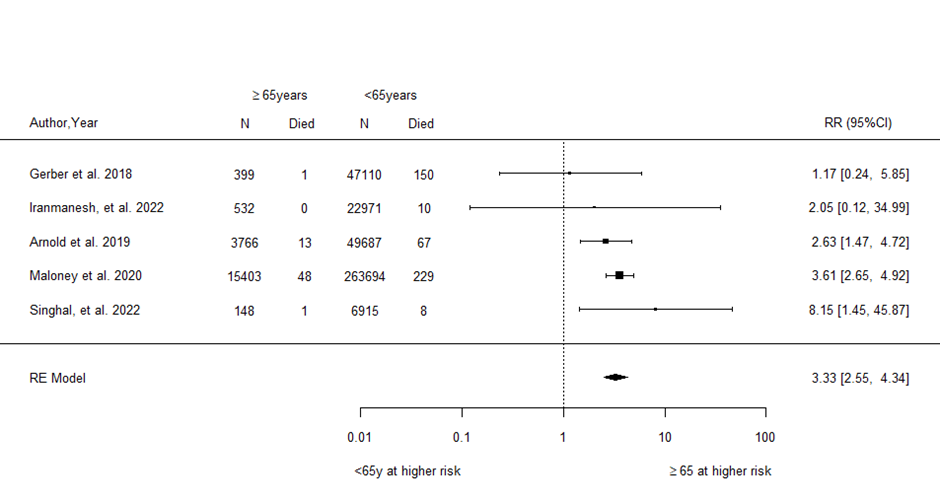


## S6.2: Anastomotic leak as a postoperative complication: sensitivity analysis excluding studies adjusted for comorbidities

A pooled analysis of six studies [100, 116, 121, 144, 148, 149] demonstrated a significantly increased relative risk of postoperative anastomotic leak among older compared to younger patients (RR = 1.46, 95% CI: 0.89-2.38, Figure S6.2). The analysis detected a moderate level of heterogeneity between studies (I² = 65.22%, Q-statistic p-value = 0.0433).

Figure S6.2: Forest plot of relative risk for postoperative anastomotic leak associated with older relative to younger age excluding studies adjusted for comorbidities

**
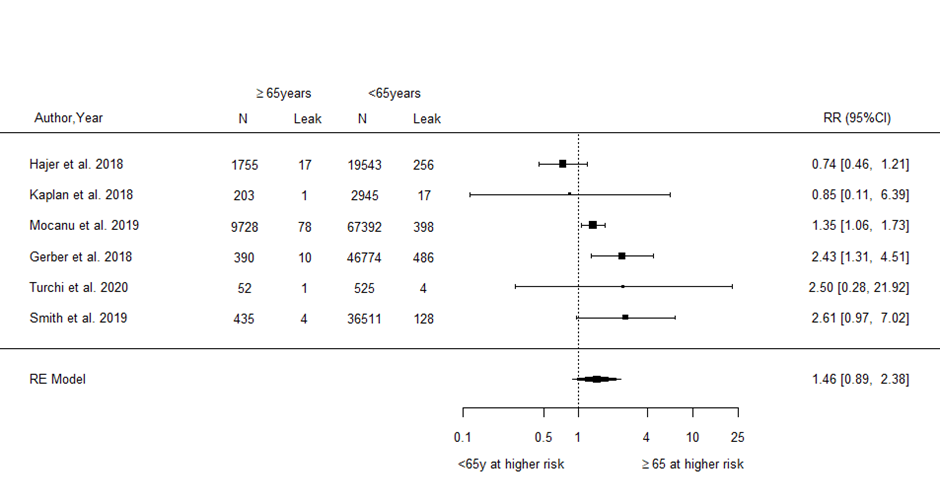
**

## S6.3: Anastomotic leak as a postoperative complication: sensitivity analysis excluding studies with different age groupings

A pooled analysis of three studies [116, 132, 149] demonstrated a significantly increased relative risk of postoperative anastomotic leak among older compared to younger patients (RR = 2.47, 95% CI: 1.50-4.07, Figure S6.3). The analysis did not detect any statistically significant evidence of heterogeneity between studies (I² = 0.00%, Q-statistic p-value = 0.2497).

Figure S6.3: Forest plot of relative risk for postoperative anastomotic leak associated with older relative to younger age excluding studies with different age groupings


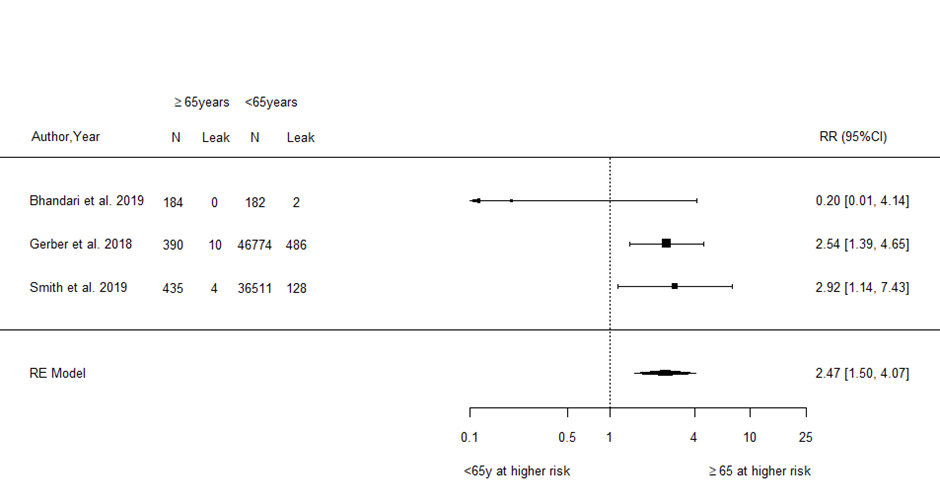


## S6.4: Haemorrhage as a postoperative complication: sensitivity analysis excluding studies adjusted for comorbidities

A pooled analysis of six studies[100, 116, 120, 122, 148, 149] demonstrated a significantly increased relative risk of postoperative haemorrhage among older compared to younger patients (RR = 1.46, 95% CI: 1.07-1.98, Figure S6.4). The analysis detected a moderate level of heterogeneity between studies (I² = 51.93%, Q-statistic p-value = 0.0796).

Figure S6.4: Forest plot of relative risk for postoperative haemorrhage associated with older relative to younger age excluding studies adjusted for comorbidities

**
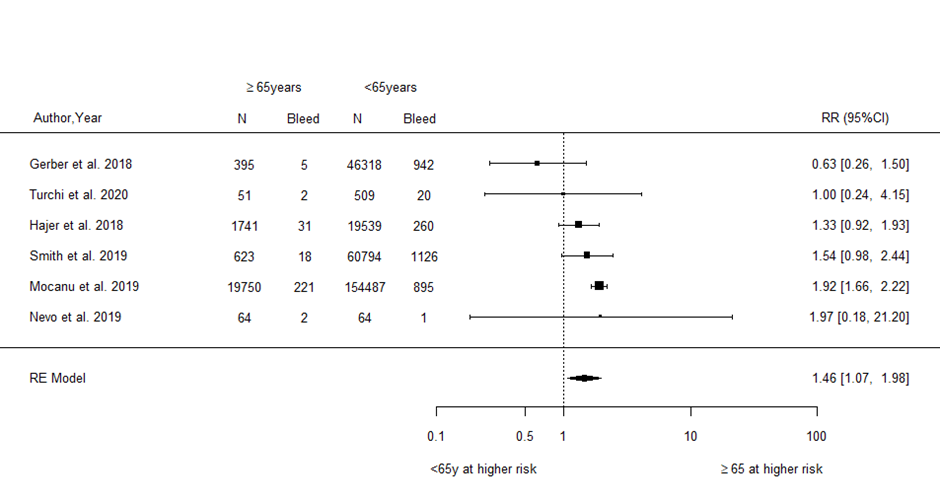
**

## S6.5: Haemorrhage as a postoperative complication: sensitivity analysis excluding studies with different age groupings

A pooled analysis of three studies[120, 132, 148] demonstrated a slightly decreased but non-significant relative risk of postoperative haemorrhage among older compared to younger patients (RR = 0.94, 95% CI: 0.39-2.26, Figure S6.5). The analysis detected a low level of heterogeneity between studies (I² = 9.39%, Q-statistic p-value = 0.3725).

Figure S6.5: Forest plot of relative risk for postoperative haemorrhage associated with older relative to younger age excluding studies with different age groupings


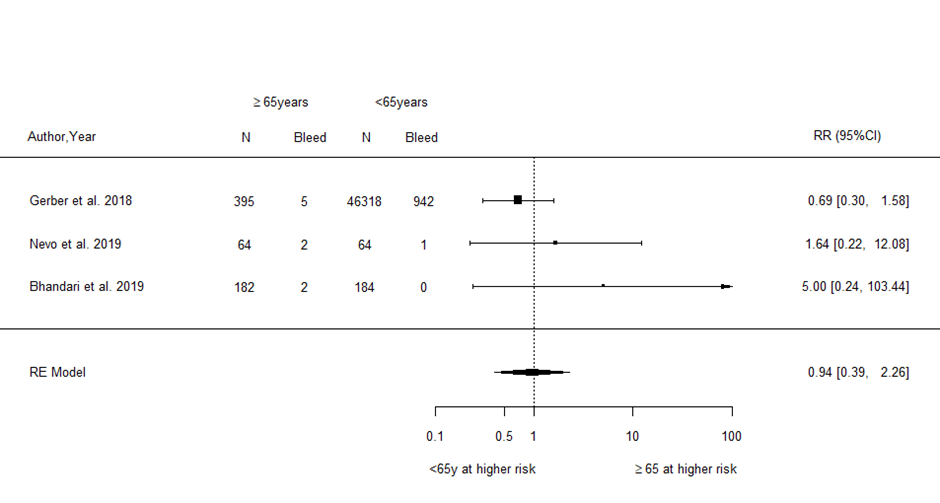


## S6.6: Serious complications as a postoperative complication: sensitivity analysis excluding one study adjusted for comorbidities and with different age groupings

A pooled analysis of three studies[91, 104, 133] demonstrated a significantly increased relative risk of postoperative serious complications among older compared to younger patients (RR = 1.65, 95% CI: 0.94-2.90, Figure S6.6). The analysis detected a very high level of heterogeneity between studies (I² = 96.10%, Q-statistic p-value < .0001).

Figure S6.6: Forest plot of relative risk for serious complications associated with older relative to younger age excluding one study adjusted for comorbidities and with different age groupings


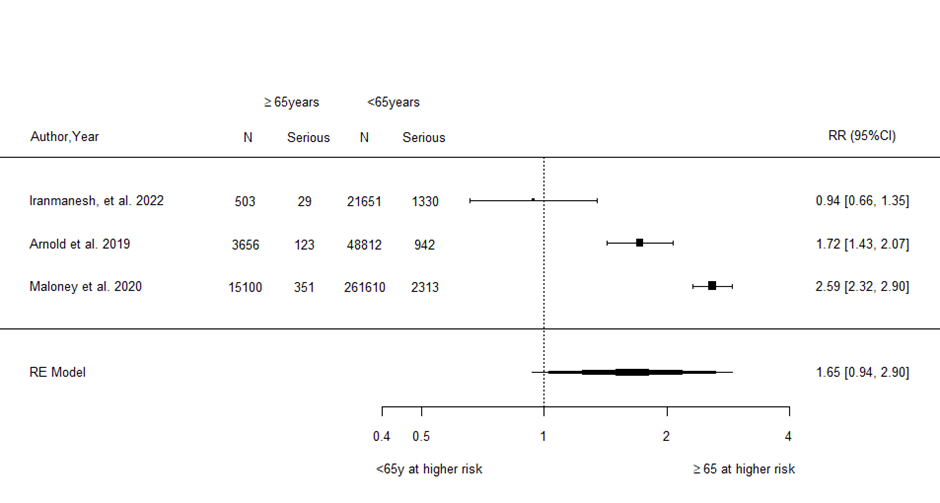


# Supplementary Section S7: References

1. Nedelcu M, Marx L, Lutfi RE, Vilallonga R, Diaconu V, Aboudi S, et al. Bariatric surgery in patients with previous COVID-19 infection. Surg Obes Relat Dis. 2021;17(7):1244-8.

2. Zafar SN, Miller K, Felton J, Wise ES, Kligman M. Postoperative bleeding after laparoscopic Roux en Y gastric bypass: predictors and consequences. Surg Endosc. 2019;33(1):272-80.

3. Pyke O, Yang J, Cohn T, Yin D, Docimo S, Talamini MA, et al. Marginal ulcer continues to be a major source of morbidity over time following gastric bypass. Surg Endosc. 2019;33(10):3451-6.

4. Stenberg E, Cao Y, Szabo E, Naslund E, Naslund I, Ottosson J. Risk Prediction Model for Severe Postoperative Complication in Bariatric Surgery. Obes Surg. 2018;28(7):1869-75.

5. Husain F, Jeong IH, Spight D, Wolfe B, Mattar SG. Risk factors for early postoperative complications after bariatric surgery. Ann Surg Treat Res. 2018;95(2):100-10.

6. Elbahrawy A, Bougie A, Loiselle SE, Demyttenaere S, Court O, Andalib A. Medium to long-term outcomes of bariatric surgery in older adults with super obesity. Surg Obes Relat Dis. 2018;14(4):470-6.

7. Dupree A, El Gammal AT, Wolter S, Urbanek S, Sauer N, Mann O, et al. Perioperative Short-Term Outcome in Super-Super-Obese Patients Undergoing Bariatric Surgery. Obes Surg. 2018;28(7):1895-901.

8. Dhar VK, Hanseman DJ, Watkins BM, Paquette IM, Shah SA, Thompson JR. What matters after sleeve gastrectomy: patient characteristics or surgical technique? Surgery. 2018;163(3):571-7.

9. Wolter S, Dupree A, Coelius C, El Gammal A, Kluwe J, Sauer N, et al. Influence of Liver Disease on Perioperative Outcome After Bariatric Surgery in a Northern German Cohort. Obes Surg. 2017;27(1):90-5.

10. Quirante FP, Montorfano L, Rammohan R, Dhanabalsamy N, Lee A, Szomstein S, et al. Is bariatric surgery safe in the elderly population? Surg Endosc. 2017;31(4):1538-43.

11. Morgan DJR, Ho KM. Incidence and outcomes after bariatric surgery in older patients: a state-wide data-linked cohort study. ANZ J Surg. 2017;87(6):471-6.

12. Coblijn UK, Karres J, de Raaff CAL, de Castro SMM, Lagarde SM, van Tets WF, et al. Predicting postoperative complications after bariatric surgery: the Bariatric Surgery Index for Complications, BASIC. Surg Endosc. 2017;31(11):4438-45.

13. Bergeat D, Lechaux D, Ghaina A, Thibault R, Bouygues V. Postoperative Outcomes of Laparoscopic Bariatric Surgery in Older Obese Patients: a Matched Case-Control Study. Obes Surg. 2017;27(6):1414-22.

14. Stenberg E, Cao Y, Jernberg T, Naslund E. Safety of bariatric surgery in patients with previous acute coronary events or heart failure: nationwide cohort study. Bjs Open. 2022;6(3):02.

15. Sebastian R, Ghanem OM, Cornejo J, Sparks A, Li C, Adrales G, et al. Validation of the cumulative deficit theory in bariatric surgery: new bariatric frailty score is associated with non-home discharge, prolonged hospital stay and mortality in the era of fast-track bariatric surgery. Surg Obes Relat Dis. 2022;18(6):779-88.

16. Samaan JS, Zhao J, Qian E, Hernandez A, Toubat O, Alicuben ET, et al. Preoperative Weight Loss as a Predictor of Bariatric Surgery Postoperative Weight Loss and Complications. J Gastrointest Surg. 2022;26(1):86-93.

17. Kaplan U, Zohdy W, Gmora S, Hong D, Anvari M. What patient factors influence bariatric surgery outcomes? A multiple regression analysis of Ontario Bariatric Registry data. Can J Surg. 2022;65(1):E66-E72.

18. Hart A, Sun Y, Titcomb TJ, Liu B, Smith JK, Correia MLG, et al. Association between preoperative serum albumin levels with risk of death and postoperative complications after bariatric surgery: a retrospective cohort study. Surg Obes Relat Dis. 2022;18(7):928-34.

19. Vosburg RW, Pratt JSA, Kindel T, Rogers AM, Kudav S, Banerjee A, et al. Bariatric Surgery is Safe for Patients After Recovery from COVID-19. Surg Obes Relat Dis. 2021;17(11):1884-9.

20. Susstrunk J, Wartmann L, Mattiello D, Kostler T, Zingg U. Incidence and Prognostic Factors for the Development of Symptomatic and Asymptomatic Marginal Ulcers After Roux-en-Y Gastric Bypass Procedures. Obes Surg. 2021;31(7):3005-14.

21. Skulsky SL, Dang JT, Switzer NJ, Sharma AM, Karmali S, Birch DW. Higher Edmonton Obesity Staging System scores are independently associated with postoperative complications and mortality following bariatric surgery: an analysis of the MBSAQIP. Surg Endosc. 2021;35(12):7163-73.

22. Shockcor N, Adnan SM, Siegel A, Wise E, Zafar SN, Kligman M. Marijuana use does not affect the outcomes of bariatric surgery. Surg Endosc. 2021;35(3):1264-8.

23. Pratt KJ, Kiser H, Ferber MF, Whiting R, Needleman B, Noria S. Impaired Family Functioning Affects 6-Month and 12-Month Postoperative Weight Loss. Obes Surg. 2021;31(8):3598-605.

24. Morrell DJ, Pauli EM, Lyn-Sue JR, Haluck RS, Rogers AM. Laparoscopic sleeve gastrectomy in patients with complex abdominal wall hernias. Surg Endosc. 2021;35(7):3881-9.

25. Morales-Marroquin E, Xie L, Uppuluri M, Almandoz JP, Cruz-Munoz N, Messiah SE. Immunosuppression and Clostridioides (Clostridium) difficile Infection Risk in Metabolic and Bariatric Surgery Patients. J Am Coll Surg. 2021;233(2):223-31.

26. Lee DU, Fan GH, Hastie DJ, Addonizio EA, Prakasam VN, Ahern RR, et al. The clinical impact of cirrhosis on the postoperative outcomes of patients undergoing bariatric surgery: propensity score-matched analysis of 2011-2017 US hospitals. Expert Rev Gastroenterol Hepatol. 2021;15(10):1191-200.

27. Iaroseski J, Harada G, Ramos R, Mottin C, Grossi J. Open Rygb Long-Term Complications: Ventral Hernia - Report on a 10-Year Single-Center Experience. Georgian Med News. 2021 (315):9-13.

28. Gomez Garcia de Las Heras S, Galindo Fernandez C, Ruiz Tovar J, Fernandez-Acenero MJ. Preoperative management of obese patients undergoing bariatric surgery: Role of endoscopy and Helicobacter eradication. Obes Res Clin Pract. 2021;15(3):289-90.

29. Bozan MB, Kutluer N, Aksu A, Bozan AA, Kanat BH, Boyuk A. Is Body Mass Index and Obesity Surgery Mortality Score Important in Perioperative Complications of Laparoscopic Sleeve Gastrectomy before Discharge? ABCD, Arquivos Brasileiros de Cirurgia Digestiva. 2021;34(2):e1602.

30. Yang J, Guan B, Huang S, Peng J, Chong TH, Wang C, et al. Different surgical techniques that influenced internal hernia prevalence rate after laparoscopic roux-en-Y gastric bypass: a retrospective analysis of 331 cases. BMC Surg. 2020;20(1):48.

31. Stenberg E, Chen R, Hilden K, Fall K. Pregnancy As a Risk Factor for Small Bowel Obstruction After Laparoscopic Gastric Bypass Surgery. Ann Surg. 2020;272(1):125-9.

32. Quezada N, Maturana G, Irarrazaval MJ, Munoz R, Morales S, Achurra P, et al. Bariatric Surgery in Cirrhotic Patients: a Matched Case-Control Study. Obes Surg. 2020;30(12):4724-31.

33. Montgomery JR, Cohen JA, Brown CS, Sheetz KH, Chao GF, Waits SA, et al. Perioperative risks of bariatric surgery among patients with and without history of solid organ transplant. Am J Transplant. 2020;20(9):2530-9.

34. Mavilia MG, Wakefield D, Karagozian R. Outcomes of Bariatric Surgery in Chronic Liver Disease: a National Inpatient Sample Analysis. Obes Surg. 2020;30(3):941-7.

35. Leonard-Murali S, Nasser H, Ivanics T, Shakaroun D, Genaw J. Perioperative Outcomes of Roux-en-Y Gastric Bypass and Sleeve Gastrectomy in Patients with Diabetes Mellitus: an Analysis of the Metabolic and Bariatric Surgery Accreditation and Quality Improvement Program (MBSAQIP) Database. Obes Surg. 2020;30(1):111-8.

36. Guggino J, Coumes S, Wion N, Reche F, Arvieux C, Borel AL. Effectiveness and Safety of Bariatric Surgery in Patients with End-Stage Chronic Kidney Disease or Kidney Transplant. Obesity. 2020;28(12):2290-304.

37. Fagenson AM, Mazzei M, Edwards MA. Does Steroid Use in Transplant Patients Undergoing Bariatric Surgery Independently Impact Outcomes? J Surg Res. 2020;254:294-9.

38. Du Y, Chen G, Zhang P, Gu Y. Effect of Previous Abdominal Surgery on Laparoscopic Roux-en-Y Gastric Bypass Surgery. J Surg Res. 2020;247:197-201.

39. Blumer V, Greene SJ, Ortiz M, Kittipibul V, Hernandez GA, Fudim M, et al. In-hospital outcomes after bariatric surgery in patients with heart failure. Am Heart J. 2020;230:59-62.

40. Are VS, Knapp SM, Banerjee A, Shamseddeen H, Ghabril M, Orman E, et al. Improving Outcomes of Bariatric Surgery in Patients With Cirrhosis in the United States: A Nationwide Assessment. Am J Gastroenterol. 2020;115(11):1849-56.

41. Altieri MS, Yang J, Zhu C, Konstantinos Spaniolas L, Talamini MA, Pryor AD. Preoperative anticoagulation in patients undergoing bariatric surgery is associated with worse outcomes. Surg Endosc. 2020;34(9):4177-84.

42. Wilkinson KH, Helm M, Lak K, Higgins RM, Gould JC, Kindel TL. The Risk of Post-operative Complications in Super-Super Obesity Compared to Super Obesity in Accredited Bariatric Surgery Centers. Obes Surg. 2019;29(9):2964-71.

43. Turk Y, Singh HK, van Huisstede A, Birnie E, Biter U, Hiemstra PS, et al. Prediction of Airflow Obstruction and the Risk of Complications in Morbidly Obese Patients Undergoing Bariatric Surgery. Obes Surg. 2019;29(9):3076-80.

44. Tewksbury C, Crowley N, Parrott JM, Andromalos L, Isom KA, Smith E, et al. Weight Loss Prior to Bariatric Surgery and 30-Day Mortality, Readmission, Reoperation, and Intervention: an MBSAQIP Analysis of 349,016 Cases. Obes Surg. 2019;29(11):3622-8.

45. Stefura T, Dros J, Kacprzyk A, Wierdak M, Proczko-Stepaniak M, Szymanski M, et al. Influence of Preoperative Weight Loss on Outcomes of Bariatric Surgery for Patients Under the Enhanced Recovery After Surgery Protocol. Obes Surg. 2019;29(4):1134-41.

46. Rottenstreich A, Kleinstern G, Levin G, Mintz Y, Elchalal U, Elazary R. Does pregnancy increase the risk of cholecystectomy following bariatric surgery? A cross-sectional cohort study. Surg Obes Relat Dis. 2019;15(10):1822-8.

47. Petrick AT, Kuhn JE, Parker DM, Prasad J, Still C, Wood GC. Bariatric surgery is safe and effective in Medicare patients regardless of age: an analysis of primary gastric bypass and sleeve gastrectomy outcomes. Surg Obes Relat Dis. 2019;15(10):1704-11.

48. Nasser H, Ivanics T, Leonard-Murali S, Shakaroun D, Genaw J. Perioperative outcomes of laparoscopic Roux-en-Y gastric bypass and sleeve gastrectomy in super-obese and super-super-obese patients: a national database analysis. Surg Obes Relat Dis. 2019;15(10):1696-703.

49. Modasi A, Dang JT, Afraz S, Hefler J, Switzer N, Birch DW, et al. Bariatric Surgery Outcomes in Patients on Preoperative Therapeutic Anticoagulation: an Analysis of the 2015 to 2017 MBSAQIP. Obes Surg. 2019;29(11):3432-42.

50. Mazzei M, Zhao H, Edwards MA. Perioperative outcomes of bariatric surgery in the setting of chronic steroid use: an MBSAQIP database analysis. Surg Obes Relat Dis. 2019;15(6):926-34.

51. Mazzei M, Zhao H, Edwards MA. The impact of chronic kidney disease on bariatric perioperative outcome: a MBSAQIP matched analysis. Surg Obes Relat Dis. 2019;15(12):2075-86.

52. Mavilia MG, Wakefield D, Karagozian R. Nonalcoholic fatty liver disease does not predict worse perioperative outcomes in bariatric surgery. Obes Res Clin Pract. 2019;13(4):416-8.

53. Lak KL, Helm MC, Higgins RM, Kindel TL, Gould JC. Preoperative functional health status is a predictor of short-term postoperative morbidity and mortality after bariatric surgery. Surg Obes Relat Dis. 2019;15(4):608-14.

54. Huang HH, Hsieh MS, Chen CY. Risk of cholecystectomy in morbidly obese patients after bariatric surgery in Taiwan. Obes Res Clin Pract. 2019;13(2):191-6.

55. Hefler J, Dang J, Modasi A, Switzer N, Birch DW, Karmali S. Effects of Chronic Corticosteroid and Immunosuppressant Use in Patients Undergoing Bariatric Surgery. Obes Surg. 2019;29(10):3309-15.

56. Han ML, Lee WJ, Chen JC, Ser KH, Chen SC, Lee YC. Clinical Characteristics and Outcome of Morbidly Obese Bariatric Patients with Concurrent Hepatitis C Viral Infection. Obes Surg. 2019;29(3):828-34.

57. Gamme G, Dang JT, Switzer N, Gill R, Birch DW, Karmali S. Evaluating the safety of bariatric surgery for weight loss in class I obesity: A propensity-matched analysis of North American data. Surg Obes Relat Dis. 2019;15(4):629-35.

58. Feng X, Andalib A, Brethauer SA, Schauer PR, Aminian A. How safe is bariatric surgery in patients with class I obesity (body mass index 30-35 kg/m<sup>2</sup>)? Surg Obes Relat Dis. 2019;15(2):253-60.

59. Chiappetta S, Stier C, Weiner RA, members of StuDo Q, Viszeralchirurgie/StuDoQ MBEoDGfA-u. The Edmonton Obesity Staging System Predicts Perioperative Complications and Procedure Choice in Obesity and Metabolic Surgery-a German Nationwide Register-Based Cohort Study (StuDoQ|MBE). Obes Surg. 2019;29(12):3791-9.

60. Chen JH, Tsai MS, Chen CY, Lee HM, Cheng CF, Chiu YT, et al. Bariatric Surgery Did Not Increase the Risk of Gallstone Disease in Obese Patients: a Comprehensive Cohort Study. Obes Surg. 2019;29(2):464-73.

61. Afraz S, Dang JT, Modasi A, Switzer N, Birch DW, Karmali S. Bariatric surgery outcomes in oxygen-dependent patients: analysis of the MBSAQIP database. Surg Obes Relat Dis. 2019;15(9):1571-80.

62. Tsamalaidze L, Stauffer JA, Arasi LC, Villacreses DE, Franco JSS, Bowers S, et al. Laparoscopic Sleeve Gastrectomy for Morbid Obesity in Patients After Orthotopic Liver Transplant: a Matched Case-Control Study. Obes Surg. 2018;28(2):444-50.

63. Strong AT, Sharma G, Tu C, Aminian A, Young JB, Rodriguez J, et al. A Population-Based Study of Early Postoperative Outcomes in Patients with Heart Failure Undergoing Bariatric Surgery. Obes Surg. 2018;28(8):2281-8.

64. Spaniolas K, Yang J, Crowley S, Yin D, Docimo S, Bates AT, et al. Association of Long-term Anastomotic Ulceration After Roux-en-Y Gastric Bypass With Tobacco Smoking. JAMA Surgery. 2018;153(9):862-4.

65. Sharma G, Nor-Hanipah Z, Haskins IN, Punchai S, Strong AT, Tu C, et al. Comparative Outcomes of Bariatric Surgery in Patients with Impaired Mobility and Ambulatory Population. Obes Surg. 2018;28(7):2014-24.

66. Serin KR, Akyuz U, Batman B, Uymaz DS, Altun H. Does Helicobacter pylori infection influence the major postoperative complication rate after sleeve gastrectomy? A retrospective cohort study in an endemic region. Turk J Gastroenterol. 2018;29(4):379-83.

67. Minhem MA, Safadi BY, Habib RH, Raad EPB, Alami RS. Increased adverse outcomes after laparoscopic sleeve gastrectomy in older super-obese patients: analysis of American College of Surgeons National Surgical Quality Improvement Program Database. Surg Obes Relat Dis. 2018;14(10):1463-70.

68. Major P, Dros J, Kacprzyk A, Pedziwiatr M, Malczak P, Wysocki M, et al. Does previous abdominal surgery affect the course and outcomes of laparoscopic bariatric surgery? Surg Obes Relat Dis. 2018;14(7):997-1004.

69. Inadomi M, Iyengar R, Fischer I, Chen X, Flagler E, Ghaferi AA. Effect of patient-reported smoking status on short-term bariatric surgery outcomes. Surg Endosc. 2018;32(2):720-6.

70. Higgins RM, Helm M, Gould JC, Kindel TL. Preoperative immobility significantly impacts the risk of postoperative complications in bariatric surgery patients. Surg Obes Relat Dis. 2018;14(6):842-8.

71. Gaillard M, Tranchart H, Maitre S, Perlemuter G, Lainas P, Dagher I. Preoperative Detection of Sarcopenic Obesity Helps to Predict the Occurrence of Gastric Leak After Sleeve Gastrectomy. Obes Surg. 2018;28(8):2379-85.

72. Fieber JH, Sharoky CE, Wirtalla C, Williams NN, Dempsey DT, Kelz RR. The Malnourished Patient With Obesity: A Unique Paradox in Bariatric Surgery. J Surg Res. 2018;232:456-63.

73. Ece I, Yilmaz H, Alptekin H, Yormaz S, Colak B, Yilmaz F, et al. Comparative Effectiveness of Laparoscopic Sleeve Gastrectomy on Morbidly Obese, Super-Obese, and Super-Super Obese Patients for the Treatment of Morbid Obesity. Obes Surg. 2018;28(6):1484-91.

74. Bazerbachi F, Sawas T, Vargas EJ, Haffar S, Deepak P, Kisiel JB, et al. Bariatric Surgery Is Acceptably Safe in Obese Inflammatory Bowel Disease Patients: Analysis of the Nationwide Inpatient Sample. Obes Surg. 2018;28(4):1007-14.

75. Watanabe A, Seki Y, Haruta H, Kikkawa E, Kasama K. Preoperative Weight Loss and Operative Outcome After Laparoscopic Sleeve Gastrectomy. Obes Surg. 2017;27(10):2515-21.

76. Stenberg E, Szabo E, Naslund I, Ottosson J. Bleeding during laparoscopic gastric bypass surgery as a risk factor for less favorable outcome. A cohort study from the Scandinavian Obesity Surgery Registry. Surg Obes Relat Dis. 2017;13(10):1735-40.

77. Singh T, Kochhar GS, Goh GB, Schauer P, Brethauer S, Kroh M, et al. Safety and efficacy of bariatric surgery in patients with advanced fibrosis. Int J Obes. 2017;41(3):443-9.

78. Shanti H, Almajali N, Al-Shamaileh T, Samarah W, Mismar A, Obeidat F. Helicobacter pylori Does not Affect Postoperative Outcomes After Sleeve Gastrectomy. Obes Surg. 2017;27(5):1298-301.

79. Schulman AR, Abougergi MS, Thompson CC. H. Pylori as a predictor of marginal ulceration: A nationwide analysis. Obesity. 2017;25(3):522-6.

80. Kaplan JA, Schecter SC, Rogers SJ, Lin MYC, Posselt AM, Carter JT. Expanded indications for bariatric surgery: should patients on chronic steroids be offered bariatric procedures? Surg Obes Relat Dis. 2017;13(1):35-40.

81. Janik MR, Waledziak M, Bragoszewski J, Kwiatkowski A, Pasnik K. Prediction Model for Hemorrhagic Complications after Laparoscopic Sleeve Gastrectomy: Development of SLEEVE BLEED Calculator. Obes Surg. 2017;27(4):968-72.

82. Haskins IN, Nowacki AS, Khorgami Z, Schulz K, Heinberg LJ, Schauer PR, et al. Should recent smoking be a contraindication for sleeve gastrectomy? Surg Obes Relat Dis. 2017;13(7):1130-5.

83. Creange C, Sethi M, Fielding G, Ren-Fielding C. The safety of laparoscopic sleeve gastrectomy among diabetic patients. Surg Endosc. 2017;31(2):907-11.

84. Lainas P, Triantafyllou E, Chague P, Dammaro C, Maitre S, Rocher L, et al. Routine Early Computed Tomography Scanner After Laparoscopic Sleeve Gastrectomy in High-Risk Severely Obese Patients Is Effective for Bleeding or Hematoma Diagnosis but not for Staple-Line Leak Detection: a Prospective Study. Obes Surg. 2022;32(5):1624-30.

85. Hogestol IK, Chahal-Kummen M, Eribe I, Brunborg C, Stubhaug A, Hewitt S, et al. Chronic Abdominal Pain and Symptoms 5 Years After Gastric Bypass for Morbid Obesity. Obes Surg. 2017;27(6):1438-45.

86. Aziz M, Haghbin H, Sharma S, Fatima R, Ishtiaq R, Chandan S, et al. Safety of bariatric surgery in patients with inflammatory bowel disease: A systematic review and meta-analysis. Clinical Obesity. 2020;10(6):e12405.

87. Sharma G, Hanipah ZN, Aminian A, Punchai S, Bucak E, Schauer PR, et al. Bariatric Surgery in Patients on Chronic Anticoagulation Therapy. Obes Surg. 2018;28(8):2225-32.

88. Vallois A, Menahem B, Le Roux Y, Meunier H, Lee Bion A, Marion Y, et al. Is laparoscopic bariatric surgery as safe and effective before and after age 60? Results from a propensity-score analysis. Surg Obes Relat Dis. 2022;18(4):520-9.

89. Singhal R, Omar I, Madhok B, Rajeev Y, Graham Y, Tahrani AA, et al. Safety of Bariatric Surgery in >= 65-Year-Old Patients During the COVID-19 Pandemic. Obes Surg. 2022;32(7):1-13.

90. Khalid SI, Maasarani S, Shanker RM, Becerra AZ, Omotosho P, Torquati A. Social determinants of health and their impact on rates of postoperative complications among patients undergoing vertical sleeve gastrectomy. Surgery. 2022;171(2):447-52.

91. Iranmanesh P, Boudreau V, Ramji K, Barlow K, Lovrics O, Anvari M. Outcomes of bariatric surgery in elderly patients: a registry-based cohort study with 3-year follow-up. Int J Obes. 2022;46(3):574-80.

92. Nafiu OO, Mpody C, Michalsky MP, Tobias JD. Unequal rates of postoperative complications in relatively healthy bariatric surgical patients of white and black race. Surg Obes Relat Dis. 2021;17(7):1249-55.

93. Mousapour P, Tasdighi E, Khalaj A, Mahdavi M, Valizadeh M, Taheri H, et al. Sex disparity in laparoscopic bariatric surgery outcomes: a matched-pair cohort analysis. Sci Rep. 2021;11(1):12809.

94. Hui BY, Khorgami Z, Puthoff JS, Kuwada TS, Lim RB, Chow GS. Postoperative sepsis after primary bariatric surgery: an analysis of MBSAQIP. Surg Obes Relat Dis. 2021;17(4):667-72.

95. Di Palma A, Liu B, Maeda A, Anvari M, Jackson T, Okrainec A. Marginal ulceration following Roux-en-Y gastric bypass: risk factors for ulcer development, recurrence and need for revisional surgery. Surg Endosc. 2021;35(5):2347-53.

96. Bal J, Ilonzo N, Adediji T, Leitman IM. Gender as a Deterministic Factor in Procedure Selection and Outcomes in Bariatric Surgery. Journal of the Society of Laparoendoscopic Surgeons. 2021;25(1):Jan-Mar.

97. Athanasiadis DI, Hernandez E, Dirks RC, Stefanidis D, Banerjee A. Postoperative 4-Year Outcomes in Septuagenarians Following Bariatric Surgery. Obes Surg. 2021;31(12):5127-31.

98. Aryannezhad S, Khalaj A, Hosseinpanah F, Mahdavi M, Valizadeh M, Barzin M. One-year outcomes of bariatric surgery in older adults: a case-matched analysis based on the Tehran Obesity Treatment Study. Surg Today. 2021;51(1):61-9.

99. Welsh LK, Luhrs AR, Davalos G, Diaz R, Narvaez A, Perez JE, et al. Racial Disparities in Bariatric Surgery Complications and Mortality Using the MBSAQIP Data Registry. Obes Surg. 2020;30(8):3099-110.

100. Turchi MJ, Kingma F, Laborda N, Montanelli A, Maldonado JM, Fiolo FE. Roux-en-Y gastric bypass in the elderly: is age a determining factor in our outcomes? Surg Obes Relat Dis. 2020;16(10):1514-20.

101. Sundaresan N, Roberts A, Thompson KJ, McKillop IH, Barbat S, Nimeri A. Examining the Hispanic paradox in bariatric surgery. Surg Obes Relat Dis. 2020;16(10):1392-400.

102. Molero J, Olbeyra R, Vidal J, Torres F, Canizares S, Andreu A, et al. A Propensity Score Cohort Study on the Long-Term Safety and Efficacy of Sleeve Gastrectomy in Patients Older Than Age 60. J Obes. 2020;2020:8783260.

103. Mocanu V, Dang JT, Switzer N, Madsen K, Birch DW, Karmali S. Sex and Race Predict Adverse Outcomes Following Bariatric Surgery: an MBSAQIP Analysis. Obes Surg. 2020;30(3):1093-101.

104. Maloney SR, Dugan N, Prasad T, Colavita PD, McKillop IH, Gersin KS, et al. Impact of age on morbidity and mortality following bariatric surgery. Surg Endosc. 2020;34(9):4185-92.

105. Hoffman AB, Myneni AA, Orom H, Schwaitzberg SD, Noyes K. Disparity in access to bariatric surgery among African-American men. Surg Endosc. 2020;34(6):2630-7.

106. Haal S, Rondagh D, Hutten BA, Acherman YIZ, van de Laar A, Huijgen R, et al. Risk Factors for Cholecystectomy After Laparoscopic Roux-En-Y Gastric Bypass. Obes Surg. 2020;30(2):507-14.

107. Gambhir S, Inaba CS, Alizadeh RF, Nahmias J, Hinojosa M, Smith BR, et al. Venous thromboembolism risk for the contemporary bariatric surgeon. Surg Endosc. 2020;34(8):3521-6.

108. Dugan N, Thompson KJ, Barbat S, Prasad T, McKillop IH, Maloney SR, et al. Male gender is an independent risk factor for patients undergoing laparoscopic sleeve gastrectomy or Roux-en-Y gastric bypass: an MBSAQIP R database analysis. Surg Endosc. 2020;34(8):3574-83.

109. Amirian H, Torquati A, Omotosho P. Racial Disparity in 30-Day Outcomes of Metabolic and Bariatric Surgery. Obes Surg. 2020;30(3):1011-20.

110. Alimogullari M, Bulus H. Predictive factors of gallstone formation after sleeve gastrectomy: a multivariate analysis of risk factors. Surg Today. 2020;50(9):1002-7.

111. Wood MH, Carlin AM, Ghaferi AA, Varban OA, Hawasli A, Bonham AJ, et al. Association of Race With Bariatric Surgery Outcomes. JAMA Surgery. 2019;154(5):e190029.

112. Walker E, Elman M, Takemoto EE, Fennern E, Mitchell JE, Pories WJ, et al. Bariatric Surgery Among Medicare Subgroups: Short- and Long-Term Outcomes. Obesity. 2019;27(11):1820-7.

113. Vidarsson B, Sundbom M, Edholm D. Incidence and treatment of leak at the gastrojejunostomy in Roux-en-Y gastric bypass: a cohort study of 40,844 patients. Surg Obes Relat Dis. 2019;15(7):1075-9.

114. Sun W, Zhang Y, Shen Q, Zhang W, Yao Q, Yang Y. Prevalence and risk factors for symptoms suggestive of hypoglycemia and early dumping syndrome after sleeve gastrectomy. Surg Obes Relat Dis. 2019;15(9):1439-46.

115. Stenberg E, Persson C, Naslund E, Ottosson J, Sundbom M, Szabo E, et al. The impact of socioeconomic factors on the early postoperative complication rate after laparoscopic gastric bypass surgery: A register-based cohort study. Surg Obes Relat Dis. 2019;15(4):575-81.

116. Smith ME, Bacal D, Bonham AJ, Varban OA, Carlin AM, Ghaferi AA, et al. Perioperative and 1-year outcomes of bariatric surgery in septuagenarians: implications for patient selection. Surg Obes Relat Dis. 2019;15(10):1805-11.

117. Sheka AC, Kizy S, Wirth K, Grams J, Leslie D, Ikramuddin S. Racial disparities in perioperative outcomes after bariatric surgery. Surg Obes Relat Dis. 2019;15(5):786-93.

118. Ozdas S, Bozkurt H. Factors Affecting the Development of Gallstones Following Laparoscopic Sleeve Gastrectomy. Obes Surg. 2019;29(10):3174-8.

119. Nickel F, de la Garza JR, Werthmann FS, Benner L, Tapking C, Karadza E, et al. Predictors of Risk and Success of Obesity Surgery. Obesity Facts. 2019;12(4):427-39.

120. Nevo N, Eldar SM, Lessing Y, Sabo E, Nachmany I, Hazzan D. Sleeve Gastrectomy in the Elderly. Obesity Facts. 2019;12(5):502-8.

121. Mocanu V, Dang J, Ladak F, Switzer N, Birch DW, Karmali S. Predictors and outcomes of leak after Roux-en-Y gastric bypass: an analysis of the MBSAQIP data registry. Surg Obes Relat Dis. 2019;15(3):396-403.

122. Mocanu V, Dang J, Ladak F, Switzer N, Birch DW, Karmali S. Predictors and outcomes of bleed after sleeve gastrectomy: an analysis of the MBSAQIP data registry. Surg Obes Relat Dis. 2019;15(10):1675-81.

123. Martin AS, Sepulveda M, Guzman F, Guzman H, Patino F, Preiss Y. Surgical Morbidity in the Elderly Bariatric Patient: Does Age Matter? Obes Surg. 2019;29(8):2548-52.

124. Ivanics T, Nasser H, Leonard-Murali S, Genaw J. Dehydration risk factors and impact after bariatric surgery: an analysis using a national database. Surg Obes Relat Dis. 2019;15(12):2066-74.

125. Guzman HM, Sepulveda M, Rosso N, San Martin A, Guzman F, Guzman HC. Incidence and Risk Factors for Cholelithiasis After Bariatric Surgery. Obes Surg. 2019;29(7):2110-4.

126. Goldberg I, Yang J, Nie L, Bates AT, Docimo S, Jr., Pryor AD, et al. Safety of bariatric surgery in patients older than 65 years. Surg Obes Relat Dis. 2019;15(8):1380-7.

127. El Chaar M, Stoltzfus J, Gersin K, Thompson K. A novel risk prediction model for 30-day severe adverse events and readmissions following bariatric surgery based on the MBSAQIP database. Surg Obes Relat Dis. 2019;15(7):1138-45.

128. Doumouras AG, Saleh F, Hong D. The effect of distance on short-term outcomes in a regionalized, publicly funded bariatric surgery model. Surg Endosc. 2019;33(4):1167-73.

129. Dang TT, Dang JT, Moolla M, Switzer N, Madsen K, Birch DW, et al. Clostridium difficile and Laparoscopic Bariatric Surgery: an Analysis of the Metabolic and Bariatric Surgery Accreditation and Quality Improvement Program Database. Obes Surg. 2019;29(6):1881-8.

130. Dang JT, Switzer N, Delisle M, Laffin M, Gill R, Birch DW, et al. Predicting venous thromboembolism following laparoscopic bariatric surgery: development of the BariClot tool using the MBSAQIP database. Surg Endosc. 2019;33(3):821-31.

131. Clapp B, Hahn J, Dodoo C, Guerra A, de la Rosa E, Tyroch A. Evaluation of the rate of marginal ulcer formation after bariatric surgery using the MBSAQIP database. Surg Endosc. 2019;33(6):1890-7.

132. Bhandari M, Mathur W, Fobi M, Kosta S. Outcomes of bariatric surgery in geriatric patients >= 65 years: single institution study. Obes Surg. 2019;29(5):1470-6.

133. Arnold MR, Schlosser KA, Otero J, Prasad T, Lincourt AE, Gersin KS, et al. Laparoscopic Weight Loss Surgery in the Elderly: An ACS NSQIP Study on the Effect of Age on Outcomes. Am Surg. 2019;85(3):273-9.

134. Almby K, Edholm D. Anastomotic Strictures After Roux-en-Y Gastric Bypass: a Cohort Study from the Scandinavian Obesity Surgery Registry. Obes Surg. 2019;29(1):172-7.

135. Tang L, Alsulaim HA, Canner JK, Prokopowicz GP, Steele KE. Prevalence and predictors of postoperative thiamine deficiency after vertical sleeve gastrectomy. Surg Obes Relat Dis. 2018;14(7):943-50.

136. Sun S, Borisenko O, Spelman T, Ahmed AR. Patient Characteristics, Procedural and Safety Outcomes of Bariatric Surgery in England: a Retrospective Cohort Study-2006-2012. Obes Surg. 2018;28(4):1098-108.

137. Schurner AM, Manzini G, Bueter M, Schadde E, Beck-Schimmer B, Schlapfer M. Perioperative surgery- and anaesthesia-related risks of laparoscopic Roux-en-Y gastric bypass - a single centre, retrospective data analysis. BMC Anesthesiol. 2018;18(1):190.

138. Moon RC, Ghanem M, Teixeira AF, De La Cruz-Munoz N, Young MK, Domkowski P, et al. Assessing risk factors, presentation, and management of portomesenteric vein thrombosis after sleeve gastrectomy: a multicenter case-control study. Surg Obes Relat Dis. 2018;14(4):478-83.

139. McCracken E, Wood GC, Prichard W, Bistrian B, Still C, Gerhard G, et al. Severe anemia after Roux-en-Y gastric bypass: a cause for concern. Surg Obes Relat Dis. 2018;14(7):902-9.

140. Major P, Wysocki M, Janik M, Stefura T, Waledziak M, Pedziwiatr M, et al. Impact of age on postoperative outcomes in bariatric surgery. Acta Chir Belg. 2018;118(5):307-14.

141. Mackay B, Zhou L, Schroeder D. Laparoscopic gastric bypass surgery: a safe and effective operation for the >=60s? ANZ J Surg. 2018;88(4):296-300.

142. Koh CY, Inaba CS, Sujatha-Bhaskar S, Nguyen NT. Outcomes of Laparoscopic Bariatric Surgery in the Elderly Population. Am Surg. 2018;84(10):1600-3.

143. Kochkodan J, Telem DA, Ghaferi AA. Physiologic and psychological gender differences in bariatric surgery. Surg Endosc. 2018;32(3):1382-8.

144. Kaplan U, Penner S, Farrokhyar F, Andruszkiewicz N, Breau R, Gmora S, et al. Bariatric Surgery in the Elderly Is Associated with Similar Surgical Risks and Significant Long-Term Health Benefits. Obes Surg. 2018;28(8):2165-70.

145. Hussan H, Ugbarugba E, Porter K, Noria S, Needleman B, Clinton SK, et al. The Type of Bariatric Surgery Impacts the Risk of Acute Pancreatitis: A Nationwide Study. Clinical and Translational Gastroenterology. 2018;9(9):179.

146. Hennings DL, Baimas-George M, Al-Quarayshi Z, Moore R, Kandil E, DuCoin CG. The Inequity of Bariatric Surgery: Publicly Insured Patients Undergo Lower Rates of Bariatric Surgery with Worse Outcomes. Obes Surg. 2018;28(1):44-51.

147. Haskins IN, Ju T, Whitlock AE, Rivas L, Amdur RL, Lin PP, et al. Older Age Confers a Higher Risk of 30-Day Morbidity and Mortality Following Laparoscopic Bariatric Surgery: an Analysis of the Metabolic and Bariatric Surgery Quality Improvement Program. Obes Surg. 2018;28(9):2745-52.

148. Hajer AA, Wolff S, Benedix F, Hukauf M, Manger T, Stroh C, et al. Trends in Early Morbidity and Mortality after Sleeve Gastrectomy in Patients over 60 Years : Retrospective Review and Data Analysis of the German Bariatric Surgery Registry. Obes Surg. 2018;28(7):1831-7.

149. Gerber P, Anderin C, Szabo E, Naslund I, Thorell A. Impact of age on risk of complications after gastric bypass: A cohort study from the Scandinavian Obesity Surgery Registry (SOReg). Surg Obes Relat Dis. 2018;14(4):437-42.

150. Dreber H, Thorell A, Torgerson J, Reynisdottir S, Hemmingsson E. Weight loss, adverse events, and loss to follow-up after gastric bypass in young versus older adults: A Scandinavian Obesity Surgery Registry study. Surg Obes Relat Dis. 2018;14(9):1319-26.

151. Cesana G, Cioffi S, Giorgi R, Villa R, Uccelli M, Ciccarese F, et al. Proximal Leakage After Laparoscopic Sleeve Gastrectomy: an Analysis of Preoperative and Operative Predictors on 1738 Consecutive Procedures. Obes Surg. 2018;28(3):627-35.

152. Altieri MS, Yang J, Nie L, Docimo S, Talamini M, Pryor AD. Incidence of cholecystectomy after bariatric surgery. Surg Obes Relat Dis. 2018;14(7):992-6.

153. Alizadeh RF, Li S, Inaba C, Penalosa P, Hinojosa MW, Smith BR, et al. Risk Factors for Gastrointestinal Leak after Bariatric Surgery: MBASQIP Analysis. J Am Coll Surg. 2018;227(1):135-41.

154. Al-Kurd A, Grinbaum R, Mordechay-Heyn T, Asli S, Abubeih A, Mizrahi I, et al. Outcomes of Sleeve Gastrectomy in Septuagenarians. Obes Surg. 2018;28(12):3895-901.

155. Spivak H, Azran C, Spectre G, Lidermann G, Blumenfeld O. Sleeve Gastrectomy Postoperative Hemorrhage is Linked to Type-2 Diabetes and Not to Surgical Technique. Obes Surg. 2017;27(11):2927-32.

156. Navarrete A, Corcelles R, Del Gobbo GD, Perez S, Vidal J, Lacy A. Sleeve gastrectomy in the elderly: A case-control study with long-term follow-up of 3 years. Surg Obes Relat Dis. 2017;13(4):575-80.

157. Major P, Wysocki M, Pedziwiatr M, Pisarska M, Dworak J, Malczak P, et al. Risk factors for complications of laparoscopic sleeve gastrectomy and laparoscopic Roux-en-Y gastric bypass. International Journal Of Surgery. 2017;37:71-8.

158. Halawani HM, Ripley-Hager CF, Naglak MC, Bonanni F, Antanavicius G. Venous thromboembolism after laparoscopic or robotic biliopancreatic diversion with duodenal switch. Ninety-days outcome of a 10 years' experience. Surg Obes Relat Dis. 2017;13(12):1984-9.
